# Dissimilar Diffusion Mechanisms of Li^+^, Na^+^, and K^+^ Ions in Anhydrous Fe-Based Prussian Blue Cathode

**DOI:** 10.1021/jacs.5c05274

**Published:** 2025-06-30

**Authors:** Dan Ito, Seong-Hoon Jang, Hideo Ando, Toshiyuki Momma, Yoshitaka Tateyama

**Affiliations:** † Graduate School of Advanced Science and Engineering, 13148 Waseda University, 3-4-1, Okubo, Shinjuku-ku, Tokyo 169-8555, Japan; ‡ Research Center for Energy and Environmental Materials (GREEN), National Institute for Materials Science (NIMS), 1-1 Namiki, Tsukuba, Ibaraki 305-0044, Japan; § Laboratory for Chemistry and Life Science, 13290 Institute of Science Tokyo, 4259 Nagatsuta-cho, Midori-ku, Yokohama, Kanagawa 226-8501, Japan; ∥ Institute for Materials Research, Tohoku University, 2-1-1 Katahira, Aoba-ku, Sendai, Miyagi 980-8577, Japan; ⊥ Faculty of Science, 13149 Yamagata University, 1-4-12 Kojirakawa-machi, Yamagata-shi, Yamagata 990-8560, Japan

## Abstract

Prussian Blue (PB, AFe­[Fe­(CN)_6_], where A = Li, Na, K, *etc.*), a three-dimensional (3D) metal–organic framework (MOF), emerges as a promising cathode material, particularly for next-generation Na- and K-ion batteries. However, the microscopic occupation positions and diffusion behaviors of A^+^ ions in the unit cell have been inadequately elucidated. This study systematically compares the diffusion mechanisms of multiple Li^+^, Na^+^, and K^+^ ions using density functional theory calculations. We clarified the new stable occupation sites for Li^+^ and Na^+^ ions: the face-centered (FC) 24d and off-FC 48g sites, respectively. The smaller ionic radii of Li^+^ and Na^+^ ions contribute to their enhanced Coulombic attractions from CN^–^ anions. Li^+^ ions are more self-diffusive than Na^+^ at high temperatures; however, at room temperature, Na^+^ ions have comparable self-diffusivities and lower activation energies than Li^+^ ions. This is attributed to the smaller tilting of [Fe­(CN)_6_]-octahedra induced by Na^+^ ions’ transfers, resulting in a shallower potential energy landscape than for Li^+^ ions. These results demonstrated that the anhydrous Fe-based pristine PB crystal is an excellent Na^+^-ion conductor. Meanwhile, K^+^ ions prefer the conventional body center (8c site) and exhibit negligible self-diffusivities without anionic defects. Surprisingly, they show anisotropic diffusion along anion vacancy channels in the defective crystal, in contrast with the isotropic pathways for Li^+^ and Na^+^ ions. These findings update the fundamental chemistry of the diffusivity correlation with the electronic orbital interactions and framework distortion within general MOF materials.

## Graphical abstract

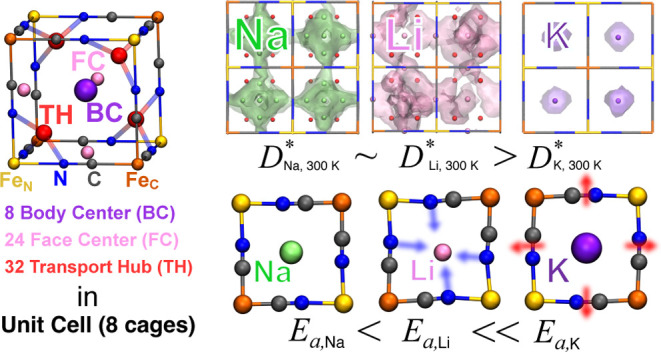

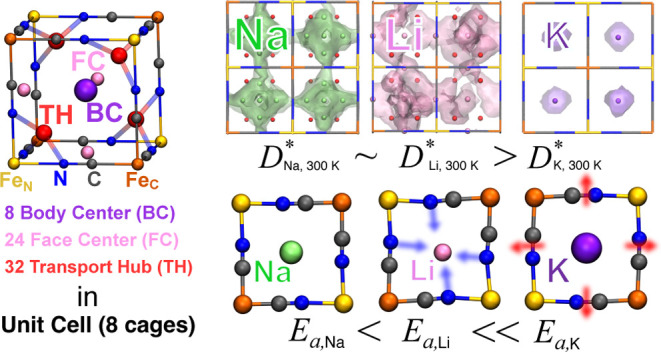

## 1 Introduction

Rechargeable batteries can contribute significantly to social sustainability.^1^ While Li-ion batteries (LIB) have been instrumental in advancing rechargeable battery technology, attention has shifted to alternative metal resources, especially due to the uneven distribution of Li in the Earth’s crust.^2^ As the diversification of rechargeable battery materials becomes increasingly important, progress in sodium-ion (NIB) and potassium-ion batteries (KIB) attracts significant attention as post-LIB^3–5^ . Three-dimensional metal–organic frameworks (MOFs) are not limited to Li-ion storage.^6^ For example, Prussian Blue (PB, *A*Fe­[Fe­(CN)] (its single cage shown in Figure 1)) and its analogues (PBA, *AM*[*M*′(CN)], where *A* denotes the ion, such as Li, Na, K, Mg, Ca, Zn, and Al,^7–16^ and the transition metals *M* and *M*′ (e.g., Fe, Cu, V, Mn, Co, Zn, Ni, Cr, Cd, *etc.* ^17–21^ )) have emerged as promising cathode materials in NIB and KIB.^22^ PB and PBAs have demonstrated experimentally long-cycle lives at high rates and impressive energy efficiency with the insertion of Na and K ions.^7–11^

**Figure 1:**
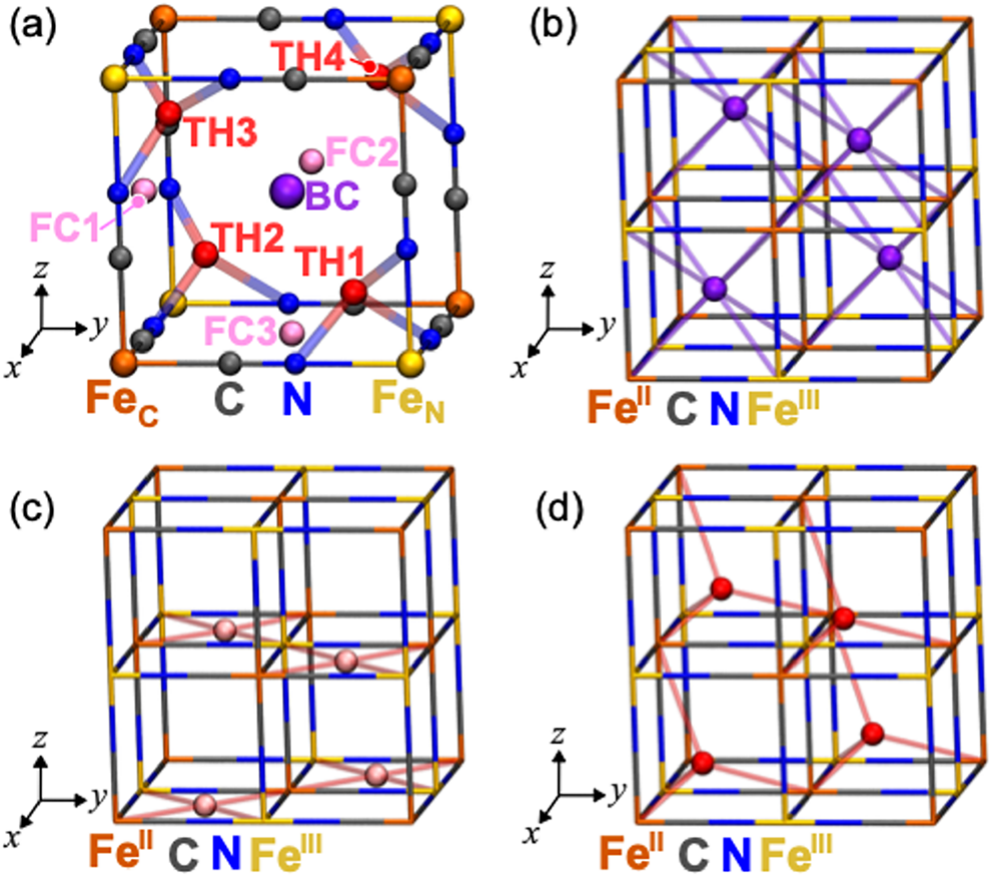
(a) Single Prussian Blue (PB) cage and its possible occupation sites. The occupation sites are body center (BC, 8c Wyckoff site, purple spheres), face-center (FC, 24d Wyckoff site, pink spheres), and transport-hub (TH, 32f Wyckoff site, red spheres). In the 2 × 2 × 2 cages, the stable site arrangements are the (b) BC sites, (c) FC sites, and (d) TH sites. The blue, gray, and yellow (orange) spheres indicate N, C, and the Fe-coordinating with N (C), respectively.

PBA has a generic formula *A*_*x*_ *M^n^* [*M*′*^m^* (CN)_6_] _1–*y*_<_*y*_ ·*z*H_2_O (0 < *x* < 2, 0 < *y* < 1), where vacancy < represents [*M*′(CN)_6_]^*m*−6^ vacancy, and the transition metal *M* (*M*′) with valences *n* (*m*), which has 6-fold coordination with N and C atoms, and *z* is the number of water molecules.^23,24^ This flexible framework design facilitates the diffusion of A^+^ ions and the transport of electrons/holes *via M* and *M*′, potentially leading to excellent battery performance.^22^ There has been a lot of research focusing on the impurity and defect effects (i.e., the densities and distribution of water molecules and anion vacancy in the crystal) to improve the performance of the PB/PBA cathode,^8–11,25,26^ but the microscopic diffusion mechanisms of A^+^ ions, even in the perfect (pristine) PB crystal, have not been well established yet. For example, as a conventional view, the pristine PB-type cathode was expected to be a good K^+^ ion conductor due to the smaller Stokes’ ionic radius of solvated K^+^ ions.^27,28^ Meanwhile, preparing defect-free PB crystals and direct experimental observation of the microscopic diffusion of A^+^ ions in the practical PB cathodes are extremely challenging,^16,29^ hindering further development of these promising materials. Therefore, a systematic theoretical approach is essential to understand the diffusivities of A^+^ ions in the pristine PB crystal.

Focusing on the local single-cage model to incorporate a single A^+^ ion, our recent theoretical investigation, employing quantum-chemical density functional theory (DFT) calculations with local (atom-centered Gaussian) basis sets, has uncovered barrierless three-dimensional pathways for a single Li^+^ ion and high-energy one-dimensional pathways for a single K^+^ ion.^29^ Despite the resulting difference in energetic and topological features of Li^+^ and K^+^ ions’ diffusion pathways, this model ignores not only the framework distortions induced by A^+^ ions but also the mutual A^+^ ion interactions. Therefore, a theoretical understanding of the A^+^ ion diffusion mechanisms within the PB/PBA unit cell with 2 × 2 × 2 cages or more is still needed. When considering the complex spin-electronic states, DFT molecular dynamics (DFT-MD) calculations with multiple A^+^ ions in the unit cell are crucially desirable.

This study investigates the stable occupation positions and diffusion mechanisms of Li^+^, Na^+^, and K^+^ in A-PB by using A_4_Fe_4_[Fe­(CN)_6_]_4_ model systems (A = Li, Na, K), prototypical PB models with cubic-shaped cages. We employed DFT with plane-wave basis-based calculations and periodic boundary conditions. We utilized the Nudged Elastic Band (NEB) method^30^ and MD sampling for the dynamics analysis. In the stable configuration search, an in-house DFT-surrogate technique, “EwaldSolidSolution”, was used.^31^ By analyzing mean-squared displacement (MSD), trajectory densities, probability densities of (dihedral) angles, and radial distribution functions (RDFs), we demonstrate the differences in the diffusion mechanisms among the A^+^ ions. Numerous past studies have examined a single A^+^ ion’s hopping scheme using the NEB method,^11,25,26,32–35^ but this study is the first to employ DFT-MD to elucidate the microscopic diffusion associated with stochastically multiple A^+^ ions’ hopping events as well as the electronic states change in the pristine PB crystals. The results significantly update the understanding of A^+^ ions’ diffusion in not only PB/PBA but also general MOF materials.

## 2 Computational Model and Method

### 2.1 Structure Model of Prussian Blue

Fe­[Fe­(CN)_6_] contains Fe ions hexa-coordinated by C atoms (Fe_C_) and N atoms (Fe_N_), both with a charge of +3 in a 1:1 stoichiometric ratio. The Fe_C_ and Fe_N_ centers are alternately arranged at the corners of each cage (brown and yellow in Figure 1a). These complexes form a free space and provide three-dimensional diffusion pathways for A^+^ ions or molecules.^17^ When the Fe ions in the framework are electrochemically reduced (oxidized), A^+^ ions are inserted from (released into) the electrolyte solution for charge compensation in a battery. AFe­[Fe­(CN)_6_], contains Fe_C_ and Fe_N_ with charges of +2 and +3.

Single A^+^ ions, even large ones (e.g., Na^+^ or K^+^ ions), can occupy a free space in the single cage.^22^ The cage contains three main categories of symmetry sites: body center positions (BC in Figure 1a), face-centers (FC in Figure 1a), and a transport-hub (TH in Figure 1a, which is displaced from BC positions toward the *N*-coordinated corner), as positions for A^+^ ion occupation.^29,36^ Within one cage, there are BC, three FCs, and four THs symmetrically equivalent sites, which are labeled in Figure 1a. Note that the BC, FC, and TH sites correspond to 8c, 24d, and 32f Wyckoff sites, respectively, in the ideal cubic-symmetry case. Previous theoretical studies have shown the relative stabilities of different interstitial occupation positions for A^+^ ions.^36^ While larger cations like K^+^ ions take BC sites as stable occupation positions, smaller cations, such as Li^+^ and Na^+^ ions, take FC and TH sites as energetically favorable positions.

The unit cell, A_4_Fe_4_[Fe­(CN)_6_]_4_, consists of four A^+^ ions, eight Fe ions (the four Fe_N_ and the four Fe_C_), and 24 CN^–^ groups, which create the four Fe­(CN)_6_ and four Fe­(NC)_6_ octahedra (Figure 1b). An Fe­(CN)_6_ octahedron and an Fe­(NC)_6_ octahedron appear alternately, interconnected through the CN bonds.^37^ The Fe ion hexa-coordinated by N atoms has a weaker ligand field and longer Fe–N bond than the Fe ion hexa-coordinated by C atoms. This causes the Fe_N_ and Fe_C_ ions to favor high spin state (*t*_2g_^3^ *e*_g_^2^ in sextet) and low spin state (*t*_2g_^6^ *e*_g_^0^ in singlet), respectively. The two cages in the *x*-, *y*-, and *z*-directions are aligned, respectively (Figure 1b–d). Focusing on three main categories of occupation sites, in total, the unit cell contains eight BC sites (i.e., double perovskite structure if all eight BC positions are occupied by A^+^ ions (Figure S1a)),^38^ 24 FC sites (Figure S1b), and 32 TH sites (Figure S1c). For A_4_Fe_4_[Fe­(CN)_6_]_4_, four of eight cages are occupied by A^+^ ions. In the following sections, we designate the 2 × 2 × 2 cages (Figure 1b–d) as the “cell”.

### 2.2 Computational Details

We used the EwaldSolidSolution method^31^ to determine the most energetically favorable arrangements of four A^+^ ions among BC, FC, and TH sites as well as charges of Fe ions. We adopted an experimental cubic structure of Fe_4_[Fe­(CN)_6_]_4_ with *Fm*3̅*m* symmetry, where the cell lengths are given as *a* = *b* = *c* = 10.191 Å.^39^ The point charge of Fe ion (*q*_Fe_), C atom (*q*_C_), N atom (*q*_N_), and the occupation sites (*q*_site_) for A^+^ ion were +2.5, −0.411, −0.589, and +1, respectively (*q*_C_ and *q*_N_ are estimated based on CN ligand’s dipole moments^40^). Further details of the point charge are found in Section S1. For A_4_Fe_4_^II/III^[Fe^II/III^(CN)_6_]_4_, the possible 3,265,920 random site arrangements for A^+^ ions and the charges of Fe positions were generated (_8_C_4_ × _8_C_4_ (4,900), _24_C_4_ × _8_C_4_ (743,820), and _32_C_4_ × _8_C_4_ (2,517,200) for BC, FC, and TH occupation sites, respectively). From those site arrangements, we selected the three arrangements with the lowest Ewald Coulombic energies for the following DFT geometry optimization.

For all DFT and DFT-MD calculations, we used the Vienna ab initio simulation package (VASP) version 6.3.2.^41,42^ Applying collinear spin polarization, we kept the total magnetization per 2 × 2 × 2 cages constant throughout the relaxation and every MD step. In this study, we assumed the ferromagnetic configuration.^43^ We used the exchange-correlation functional parametrized by the Perdew–Burke–Ernzerhof (PBE) generalized gradient approximation (GGA).^44^ In order to accurately characterize the localization of 3*d*-electrons in Fe atoms, we employed the GGA+*U* approach.^45^ Specifically, we utilized an effective on-site Coulomb repulsion of 5.0 eV for Fe ions (*U*_Fe_), a value obtained from the existing literature.^46^ van der Waals (vdW) interaction is essential to describe the microporous coordination of PB;^47^ thereof, we employed Grimme’s dispersion correction D3.^48^ We also replaced inner electrons with plane-wave projector augmented wave (PAW) representations.^49^ The valence electrons are 2s^1^ for Li, 2p^6^3s^1^ for Na, 3p^6^4s^1^ for K, 3pd^12^4s^2^ for Fe, 2s^2^2p^2^ for C, and 2s^2^2p^3^ for N, respectively. To keep accuracy consistent for DFT and DFT-MD, we used a cutoff energy of 520 eV.

In current static DFT calculations, we adopted 4 × 4 × 4 Monkhorst–Pack *k*-point grids and highly dense meshes for fast-Fourier transform (FFT) grids. We performed the geometry optimizations until the electronic total energy convergence and Hellman-Feynman force on each atom were below 10^–7^ eV and 0.3 × 10^–3^ eV/Å, respectively. To keep the framework as a cubic cell, we manually optimized the cell length and relaxed all coordinates of A^+^ ions and the framework’s atoms (C/N/Fe). We calculated the Hessians with regard to the coordinates of the A^+^ ions to confirm if there were no imaginary modes at the occupation positions (FC/off-FC/TH/BC, off-FC displaced from FC positions along the *x*-direction, as discussed in Section 3.1), with an energy convergence criterion of less than 10^–7^ eV for each ion. If imaginary modes existed, we reoptimized them to remove any imaginary frequencies.^50^

We employed the NEB method to estimate the activation energies (*E*_a_) of possible diffusion pathways for A^+^ ions between the occupation positions. During the NEB calculation, we relaxed all coordinates of A^+^ ions and the framework’s atoms (C/N/Fe). In this study, we compared the *E*_a_ of two different diffusion modes, concerted diffusion of A^+^ ions and single hopping of A^+^ ions, between the occupation positions. The single ion hopping mode was expressed by displacing an A^+^ ion between the occupation positions, while the concerted mode was generated by simultaneously displacing four A^+^ ions between the occupation positions. Note that the distance between A^+^ ions in the concerted mode is constant.

In the present DFT-MD calculations, we took the initial geometries from stable structures obtained through the DFT geometry optimization. To balance the accuracy and the computational costs, we used an energy convergence of less than 10^–5^ eV, the Γ point, and dense meshes for FFT grids. Our systems were first thermally equilibrated in a canonical (NVT) ensemble with a time step of 2 fs for 10 ps with temperature control using a Nosé–Hoover thermostat.^51,52^ We then performed a time step of 2 fs for a 100 ps MD production run for *T* = 300, 400, 500, 600, 700, and 1000 K. During DFT-MD calculations, we did not fix the positions of atoms of PB frameworks.

We used visual molecular dynamics (VMD) software^53^ for the visualization of structures and MD trajectories. For the postprocess, we utilized MDTraj,^54^ pymatgen,^55^ and VASPKIT.^56^

## 3 Results and Discussion

### 3.1 Stable Cage Occupations and Stability of Occupation Positions

With the EwaldSolidSolution method, we screen the energetically probable site arrangements for four A^+^ ions and valence states of 8 Fe ions in A_4_Fe_4_^II/III^[Fe^III/II^(CN)_6_]_4_. Through the screening, we assume that the A^+^ ions occupy the three categories of the Wyckoff sites (8c (BC), 24d (FC), and 32f (TH)) in the 2 × 2 × 2 cages (see Figure S1). We seek site arrangements with the lowest Ewald sum (energy) among Wyckoff sites in the same category as well as across three different site categories.

In the search within the same site category for the BC, FC, and TH sites, we identify that the site arrangements with the lowest Ewald energy are those where the cages occupied by the A^+^ ions are arranged adjacent to empty cages. This arrangement, referred to as “single edge-sharing”, is illustrated in Figure 1b–1d. Notably, for the single edge-sharing pattern of the BC and FC sites, the A^+^ ions are located on σ_d_ and σ_d_^′^ mirror planes. For three different site categories, the four A^+^ ions maintain a minimum separation of 7.212 Å from one another. This result can be rationalized by minimizing Coulombic repulsions among A^+^ ions. Furthermore, the valence states of four Fe_C_ and four Fe_N_ ions are +2 and +3, respectively, which are the same as the experimental valence states of the Fe ions in the PB crystal.^17^

When site arrangements across three different Wyckoff site categories are allowed, the minimum-energy site arrangements are those in which all four A^+^ ions occupy 24d (FC) Wyckoff sites. Hence, the site arrangement of A^+^ ions occupying different site categories is not energetically stable in the EwaldSolidSolution analysis. Therefore, we select three energetically different site arrangements from the lowest Ewald energies and use them as the initial geometries for DFT geometry optimizations. Supporting results confirming the success of our site arrangement screening using EwaldSolidSolution analysis are found in Section S1. We discuss the results in the following DFT sections based on the geometries for the BC, the FC, and the TH sites with the lowest DFT energy among the three different site arrangements.

Using the preliminary geometries obtained via the EwaldSolidSolution method, we perform geometry and cell optimizations at the DFT level. For the BC positions occupied by A^+^ ions, the optimized frameworks display *F*4̅3m symmetry and 10.3, 10.35, and 10.4 Å cell lengths for four Li^+^, Na^+^, and K^+^ ions, respectively (Table S1 and Figure S2d,h,l). The result indicates that the cell lengths are mainly determined by the framework components and not the size of the inserted A^+^ ions. As we expected, four K^+^ ions take the BC positions as the most stable positions, while four Li^+^ and Na^+^ ions prefer to occupy the FC positions, keeping their mutual separations (Table 1). The difference from the K^+^ ions can be explained by the ionic radius. In the cage, a larger ionic radius of K^+^ ion is expected to generate stronger electronic orbital repulsions with the framework than that of Li^+^ and Na^+^ ions. In Section 3.5, we discuss the repulsions between K^+^ ions and the framework when K^+^ ions occupy the FC positions.

**Table 1:** Relative DFT Energies (meV cell^–1^) of the Geometries of the A^+^ Ions (A = Li, Na, K) at the Four Different Occupation Positions ((off-)­FC/TH/BC)^a^

occupation positions	Li^+^	Na^+^	K^+^
FC	0.0^b^	150	3912
off-FC	not converged	0.0^b^	not converged
TH	216^b^	462^b^	not converged
BC	1338	432^b^	0.0^b^

a The energy scale is relative to the most stable occupation position for each A^+^ ion. We designate the label “not converge” for instances where the geometry optimization of the initial positions of the A^+^ ions, as specified in the first column, does not converge. In such cases, the optimized positions are found to be insufficiently close to the corresponding initial positions.

b No imaginary vibrational modes for A^+^ ions.

Hessian matrices regarding the four Na^+^ ions at the FC positions are found to have the imaginary-vibration modes. Note that the TH and the BC positions do not possess the imaginary modes. Therefore, we reoptimize the coordinates of four Na^+^ ions at the FC positions along the imaginary modes and confirm that the Na^+^ ions positions in the most stable geometry are displaced by 1.04 Å along the *x*-axis from the FC positions, named as “off-FC” positions (ideally 48g Wyckoff sites in Figures S3f and S4f). DFT cell optimizations for four Na^+^ ions at off-FC positions found 10.35 Å as the optimized cell length (Figure S2f), and the geometry is more stable than the FC positions by 150 meV/cell (Table 1). Similarly, with respect to the energy of four Na^+^ ions occupying the off-FC positions, the relative energies for the TH and the BC positions are 462 and 432 meV/cell (Table 1), respectively.

Furthermore, employing the DFT geometry optimization of four Li^+^ ions from the off-FC positions, we confirm that Li^+^ ions do not take the off-FC positions as a stationary geometry. By calculating the Hessians regarding the coordinates of four Li^+^ ions at the FC, TH, and BC positions, we confirm that no imaginary-vibration modes exist for the FC and TH positions, while they appear at the BC positions. Hence, taking the geometry of four Li^+^ ions at the FC positions as the energy reference, we find that the relative energies of four Li^+^ ions occupying the TH and the BC positions are 216 and 1338 meV/cell (Table 1), respectively. Thus, the TH occupation positions are stationary arrangements for four Li^+^ ions, in addition to the lowest energy FC occupations.

Using the geometry of four K^+^ ions at the BC positions as the energy reference, we find that the relative energy of four K^+^ ions occupying the FC positions is 3912 meV/cell (Table 1). Additionally, DFT geometry optimizations of the FC positions reveal that the optimized framework exhibits outward distortion of the Fe–N bonds surrounding K^+^ ions (Figures S3i, S4i, and Table S1). Consequently, as K^+^ ions approach the framework, they exhibit stronger electronic orbital repulsions with CN ligands due to their larger ionic radius compared to Li^+^ and Na^+^ ions.

Previous DFT (PBE + U) investigation reported that in the primitive cell (i.e., a single cage with 15 atoms), Li^+^ and Na^+^ ions preferentially occupy the off-FC and the FC positions, respectively,^36^ which is opposite to the current results. To ascertain whether the FC and off-FC positions represent the most stable configurations for Li^+^ and Na^+^ ions in the 2 × 2 × 2 cages, we evaluate the stability using various DFT functionals, including PBE + U and r2SCAN + rVV10.

For Na^+^ ions, to examine the contribution of vdW interactions, we compared the DFT energies obtained from single-point calculations for optimized structures generated from PBE + U + D3 calculations. Our calculations derived from both functional settings indicate that the energies, accounting for vdW interactions and exhibiting shorter cell lengths that are close to the experimental values, are lower for the off-FC positions than for the FC positions (see Table S2 and Figure S5). Considering the PBE functional’s propensity to overestimate cell lengths, the current results incorporating vdW interactions suggest that the off-FC position is more plausible for the anhydrous pristine Fe-based PB crystal.

We further examine the contributions from cell optimizations to the positional stability of the (off-)­FC positions for Na^+^ ions. Geometry optimization results derived from both functional settings also reveal off-FC to be more stable than FC positions (Table S3). Additionally, for both functional settings, Li^+^ ions consistently prefer the FC position (Table S4).

In Section S2, we discuss the valence states of Fe ions, such as the on-site local magnetic moments, and the comparison of geometric parameters for the A^+^ ions occupying the stable occupation positions.

### 3.2 Self-Diffusivities and Diffusion Pathways via DFT-MD Calculations

DFT-NEB method estimates the *E*_a_ (*E*_a_^NEB^) of a predefined diffusion pathway, thereof generally leaving questions regarding the validity of the *E*_a_^NEB^. Conversely, MD is a powerful method to estimate statistically appropriate *E*_a_ and enable us to analyze the diffusion characteristics of A^+^ ions, such as self-diffusion coefficients (D*) and diffusion pathways. Employing the DFT-MD method, we analyze the MSDs over a temperature range of 300–700 K, the *E*_a_ derived from the Arrhenius plot (*E*_a_^MD^), and the A^+^ ions’ trajectory densities at various temperatures to elucidate the diffusion mechanisms of A^+^ ions.

Figure 2a,b illustrates the MSDs of the Li^+^ and Na^+^ ions. At 300 K, the D* for Li^+^ and Na^+^ ions are 5.83 × 10^–6^ and 7.98 × 10^–6^ cm^2^ s^–1^, respectively, while, at 700 K, those for Li^+^ and Na^+^ ions are 1.05 × 10^–4^ and 3.81 × 10^–5^ cm^2^ s^–1^, respectively. Thus, Na^+^ ions at room temperature and Li^+^ ions at high temperature exhibit superior self-diffusivities. In fact, the Arrhenius plot exhibits the *E*_a_^MD^ = 140 and 70 meV for Li^+^ and Na^+^ ions. (Figure S6). As a primitive estimation of the statistical error associated with the D* of Li^+^ and Na^+^ ions, we estimate the Arrhenius plot of their averaged D* (⟨D*⟩) among three 50 ps production runs (Figure 2c) and exhibit *E*_a_^MD^ = 109 and 70 meV for Li^+^ and Na^+^ ions. At 300 K, that for Li^+^ ions is a comparable order to that of Na^+^ ions, demonstrating that Li^+^ and Na^+^ ions have comparable self-diffusivity at room temperature. The *E*_a_^MD^ of Li^+^ ions is higher than that of Na^+^ ions. Based on the above analysis, we conclude that the anhydrous pristine Fe-based PB crystal is an excellent Na^+^-ion conductor at room temperature.

**Figure 2:**
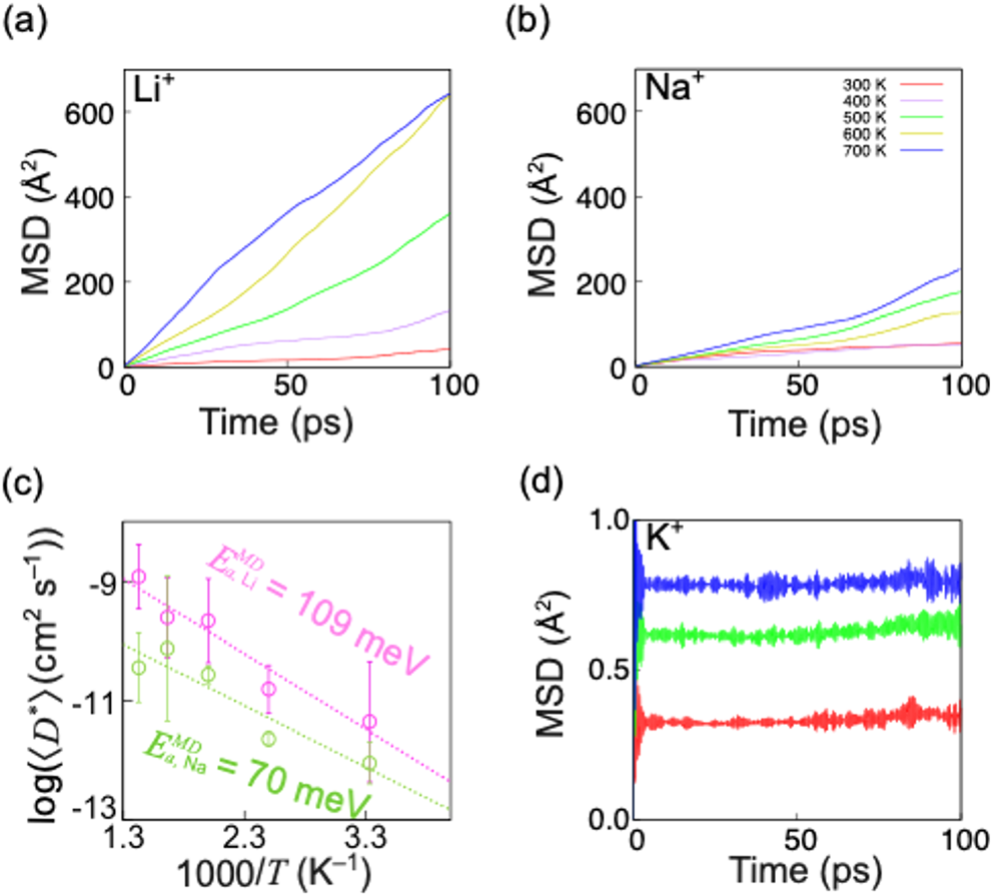
Mean-squared displacements (MSDs) for (a) Li^+^, (b) Na^+^ ions, and (c) the Arrhenius plot of the self-diffusivities over a temperature range of 300–700 K for Li^+^ and Na^+^ ions. (d) MSD for K^+^ ions. For statistical error of the Arrhenius plot for Li^+^ and Na^+^ ions, we took a 95% confidence interval of three 50 ps simulations with different initial velocities as their error bars. We excluded the first 10 ps of the MD simulations because the system is equilibrated. Good regression lines (*R* ^2^ as 0.94 and 0.76 for Li^+^ and Na^+^, respectively) were used to estimate the *E*_a_^MD^ for Li^+^ and Na^+^ ions.

Meanwhile, K^+^ ions have small MSDs (<1.2 Å^2^) at 300, 500, and 700 K, which do not show the effective slopes (Figure 2d). Therefore, K^+^ ions’ diffusion events might require large amounts of thermal energy in the anhydrous pristine Fe-based PB crystal. In Section 3.5, using DFT-NEB analysis, we discuss the energetic and geometric reasons why K^+^ ions have significantly small MSDs at either temperature.

To understand the mechanism of why Na^+^ ions at room temperature and Li^+^ ions at high temperature exhibit superior self-diffusivities than Li^+^ ions, we visualized trajectories of Li^+^ and Na^+^ ions at 300 and 700 K. At 300 K, the trajectory densities of Li^+^ ions are confined to a limited number of FC and TH positions (Figure 3a), whereas those of Na^+^ ions display extensive distributions across all off-FC and TH positions (Figure 3b). At 700 K, the trajectories for Li^+^ ions display extensive distributions across all FC and TH positions, compared to those at 300 K (Figure S7a), while Na^+^ ions do not exhibit significant changes in comparison to 300 K (Figure S7b).

**Figure 3:**
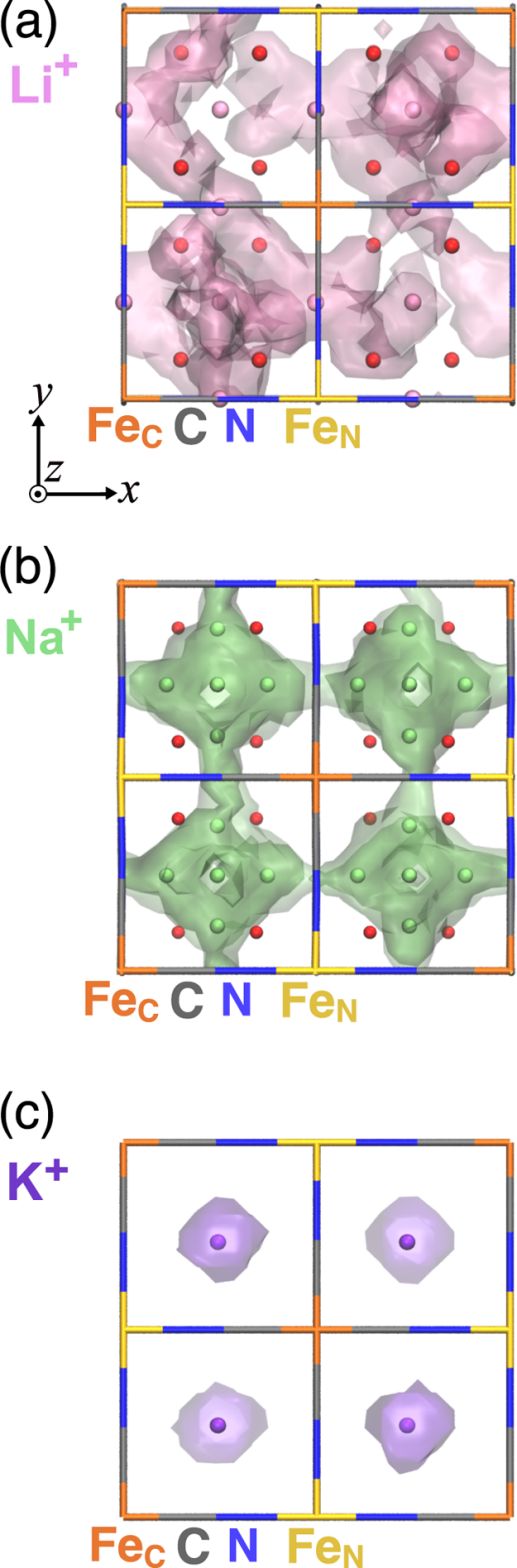
Trajectory densities at 300 K and Wyckoff occupation sites (8c (BC, purple sphere), 24d (FC, pink sphere), 32f (TH, red sphere), and 48g (off-FC, lime sphere) sphere) for all A^+^ (A *=* Li^+^ (a), Na^+^ (b), and K^+^ (c) ions) accumulated for 100 ps simulations. Each panel displays the isosurfaces with an isovalue of 5.0 × 10^–3^ Å^–3^. We excluded the first 10 ps of the MD simulations because the system is equilibrated. The blue, gray, yellow, and orange lines indicate N, C, Fe-coordinating with N, and Fe-coordinating with C, respectively.

At either temperature, Na^+^ ions take the diffusion pathways closer to the BC positions than Li^+^ ions, despite their overall three-dimensional distribution being similar to that of Li^+^ ions. This is further supported by the RDF peaks, which appear at longer distances between Na^+^ ions and framework atoms (C atoms, N atoms, and Fe ions) compared to those of Li^+^ ions (Figure S8a–f). The superior self-diffusivities of Na^+^ ions at room temperature can be attributed to their further distances from the framework, leading to weaker Coulombic attractions from CN^–^ anions than Li^+^ ions. Hence, the larger ionic radius of Na^+^ ions compared to that of Li^+^ ions results in weaker attractions between the Na^+^ ions and the framework. At high temperatures, Li^+^ ions acquire larger amounts of kinetic energy, thereby experiencing lowered trapping by CN^–^ anions.

To better understand the difference of the A^+^ ions migration behavior between the occupation positions, we analyze the occupancy of the occupation positions. Li^+^ and Na^+^ ions migrate between the FC and the off-FC positions, respectively, while K^+^ ions stay around the BC positions (Figures S9, S10, and Table S5). Supporting discussion of the occupancy of the occupation positions is described in Section S5.

Furthermore, K^+^ ions’ trajectory densities at 300 and 700 K exhibit localized solely around the original BC positions (Figures 3c and S7c), displaying no diffusion events between the BC positions. In fact, the localized RDF peaks between K^+^ ions and framework’s atoms appear at further distances (3.23, 3.18, and 3.94 Å for C, N, and Fe, respectively; Figure S8g–i) than Li^+^ and Na^+^ ions. Hence, K^+^ ions are kept around the BC positions of the framework and do not exhibit effective self-diffusion at either temperature. Our present result suggests that the cage face size is too small for K^+^ ions to diffuse between the BC positions, which revises the conventional view that the pristine PB-type cathode was expected to be a good conductor of K^+^ ions, due to the small Stokes’ ionic radius of solvated K^+^ ions.^26–28^ By using NEB analysis, we discuss the reason why the pristine PB-type cathode is not a good K^+^ ion conductor in Section 3.6.

Finally, we point out the critical importance of the DFT conditions for MD calculations to accurately evaluate the self-diffusion characteristics of A^+^ ions. This point may similarly impact studies of other MOF-type materials. We systematically screen the parameters of DFT calculations on the MSDs of Li^+^ and Na^+^ ions to identify feasible DFT calculation settings for the reliable comparison of their self-diffusivities (Table S6 and Figures S11–S24). Our selection factors include Hamiltonian conditions—such as spin polarization, the effective *U*_Fe_, and Grimme’s D3 dispersion correction—alongside self-consistent field (SCF) conditions, such as cutoff energy, energy convergence threshold, FFT grid density, and the PAW treatment of Li^+^ and Na^+^ ions. Additionally, we also examine the different methods for integrating electron occupancies, specifically the Gaussian and Fermi smearing methods.

We diagnose the MSDs during 10 ps at 300, 500, and 700 K using each set of the DFT parameters. We require that the MSDs demonstrate (i) effective D* for Li^+^ and Na^+^ ions at room temperature (300 K), (ii) a strong correlation (*R* ^2^ > 0.9) between effective D* and temperature, and (iii) effective D* increase with temperature. Our findings highlight the essential roles of Grimme’s D3 dispersion correction, spin polarization, and the effective *U*_Fe_ in accurately modeling *E*_a_ (Figures S11–S13, S24a, and S24b). The consistent employment of Grimme’s dispersion correction, spin polarization, and the effective *U*_Fe_ reveals that the choice of electron occupancy integration method significantly influences MSDs, particularly for Li^+^ ions at 300 K (Figure S14).

Furthermore, our investigations under computationally cheap SCF conditions—specifically, a 400 eV cutoff energy, 10^–4^ eV energy convergence, and sparse FFT grid density—reveal that Na^+^ ions have the MSDs at low temperature exceed the one at high temperature (see Figures S15–S17, S19–S23, S24c, Tables S7 and S8). These findings underscore that Na^+^ ions require more computationally expensive SCF conditions for enhanced Arrhenius compliance, especially for a long (100 ps) production run (refer to Figures S22 and S24c). To compare the effective D* of Li^+^ and Na^+^ ions, we adopt a computationally demanding SCF condition, including a 520 eV cutoff energy, 10^–5^ eV energy convergence, and dense FFT density.

The accuracy of DFT calculations is crucial for a detailed shape of the potential energy landscape; notably, Na^+^ ions are associated with a shallower potential energy landscape. Consequently, the precision of the Hellmann–Feynman forces acting on each atom at every MD step should be improved by employing computationally expensive SCF and Hamiltonian calculations. Thus, we emphasize the effect of the Hamiltonian and SCF conditions on the self-diffusivities of the A^+^ ions in the PB crystal as well as other MOF-type materials. Further validation of our DFT-MD methodology is discussed in Section S6.

### 3.3 Potential Energy Landscapes and Dynamical Framework Distortions for the Li^+^ (Na^+^) Ions

We find that Na^+^ ions at room temperature and Li^+^ ions at elevated temperatures exhibit superior self-diffusivity, based on DFT-MD calculations. This conclusion is impossible to find by using DFT-NEB calculations alone. The objective of this subsection is to understand the difference between D* and *E*_a_^MD^ for Li^+^ and Na^+^ ions in relation to the ordered arrangements of A^+^ ions and the framework dynamics.

To examine the probability of the ordered arrangements of A^+^ ions and the framework distortion’s modes, we analyze the RDFs between A^+^ ions and the probability density for octahedral tilting angles of the framework. For the RDF at 300 K, Li^+^ ions display two sharper peaks, compared to those of Na^+^ ions (Figure 4a, reordered arrangements than Na^+^ ions). For the RDF at 700 K, Li^+^ ions display a wider broadening peak than that of Na^+^ ions; thereof, Na^+^ ions take less disordered arrangements than that of Li^+^ ions at high temperature.

**Figure 4:**
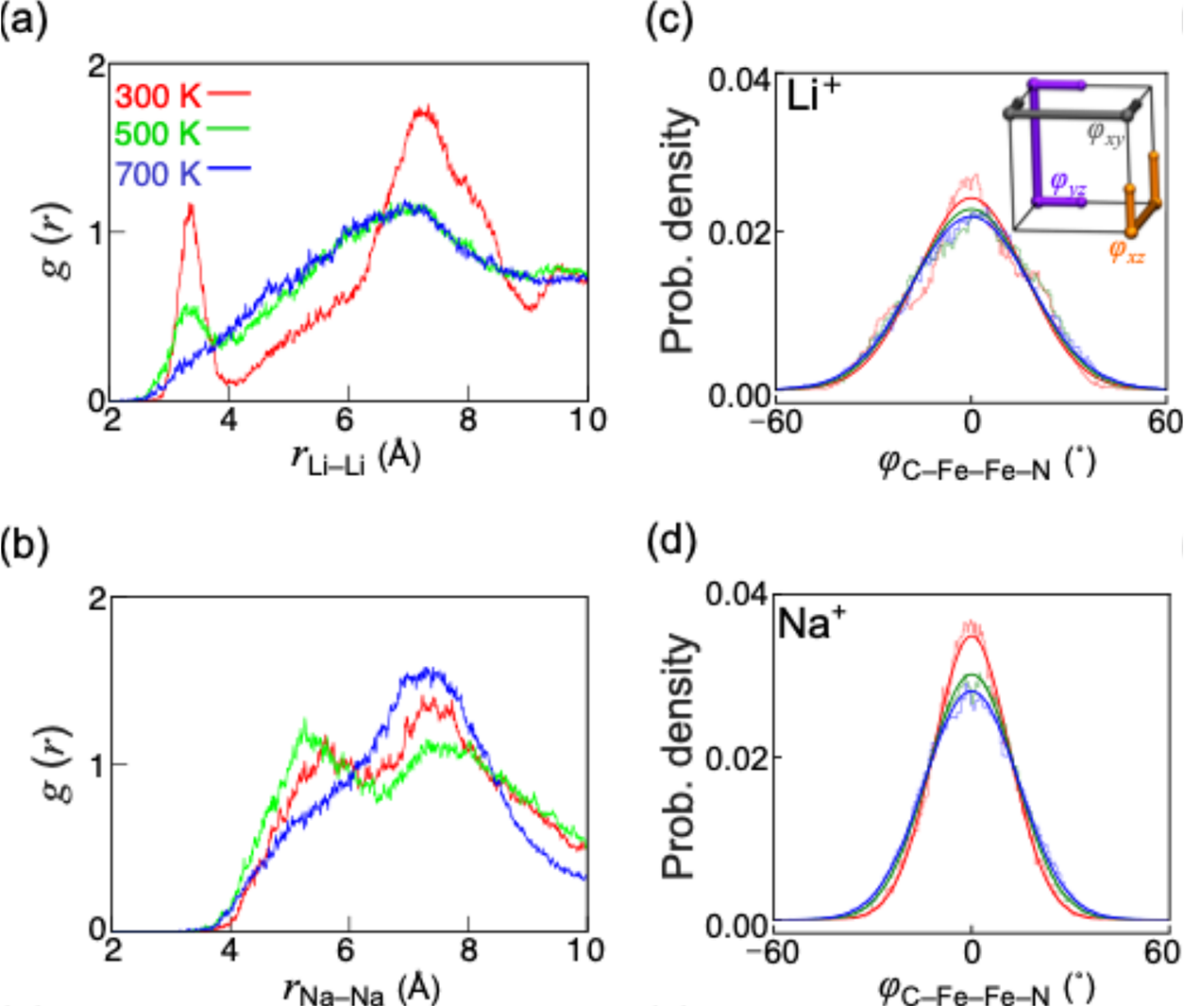
Radial distribution functions (RDF, *g*(r)) between A^+^ ions (A *=* Li^+^ and Na^+^) and probability densities for the C–Fe_C_–Fe_N_–N dihedral angles (φ_C–Fe–Fe–N_) as the tilt angle. The red, green, and blue lines correspond to the results at 300, 500, and 700 K. The RDFs are obtained by calculating each distance for four A^+^ ions (200,000 in total) and then dividing the probability by a 0.01 Å bin width. The probability densities of φ_C–Fe–Fe–N_ for the (c) Li^+^ and (d) Na^+^ ions. The probability densities of φ_C–Fe–Fe–N_ are presented for (c) Li^+^ and (d) Na^+^ ions, where dashed and solid lines correspond to histograms and probability density functions, respectively. To mitigate directional dependence, we selected three angles (φ_*j*_, *j*∈{*xy*, *xz*, *yz*}) for a single cage, as illustrated in the inset of panel (c), and averaged the probabilities across these angles. We excluded the first 10 ps of the MD simulations to ensure the system reached equilibrium before analysis. The probability densities were calculated with a bin width of 0.7°.

For instance, at 300 K, the peaks for Li^+^ ions at 3.39 and 7.17 Å correspond to configurations where multiple Li^+^ ions occupy the same cage and where the occupied cages share a single edge, respectively (Figure 1c). For Na^+^ ions, the peaks at 5.6 and 7.23 Å correspond to the geometries of the adjacent cages occupied by Na^+^ ions sharing a face and an edge (Figure 1c), respectively. As the temperature increases (green and blue lines in Figure 4a,b), for Li^+^ ions, no distinct peak at 3.39 Å appears. For Na^+^ ions, the heights of the two peaks at 500 K are comparable, and the RDF at 700 K takes a single less broadened peak at 7.23 Å (blue line in Figure 4b), compared to that of Li^+^ ions, suggesting their comparable energetic preference (green line in Figure 4b). Hence, Li^+^ ions at high temperature and Na^+^ ions at low temperature are mainly associated with less ordered arrangements. Consequently, Li^+^ ions at an elevated temperature and Na^+^ ions at a low temperature exhibit high D*s.

At low temperatures, Li^+^ ions prefer ordered arrangements due to their small ionic size and the Coulombic attractions from the CN^–^ anions, resulting in a potential energy landscape with deep basins. In contrast, disordered arrangements for Na^+^ ions arise from their larger ionic size and the weaker Coulombic interactions with CN^–^ anions, which is supported by the RDF between Li^+^ (Na^+^) and C and N atoms (Figure S8a,b,d,e). As the temperature increases, Li^+^ ions adopt more disordered arrangements because their smaller ionic size allows them to occupy more diverse positions than Na^+^ ions.

To understand the difference between the *E*_a_^MD^ of Li^+^ and Na^+^ ions in relation to the framework dynamics, φ_C–Fe–Fe–N_ dihedral angles in the *xy*- (φ_*xy*_), *xz*- (φ_*xz*_), and *yz*- (φ_*yz*_) planes can be used as the main octahedral tilting angles. The specific locations of φ_*xy*_, φ_*xz*_, and φ_*yz*_ are shown in the inset in Figure 4c. Across 300, 500, and 700 K, the probability densities of the φ_C–Fe–Fe–N_ angles are calculated by averaging the values of φ_*xy*_, φ_*xz*_, and φ_*yz*_. Additionally, we analyze the θ_N–Fe–N_ angles formed from two perpendicular Fe_N_–N, which can be used to classify the distortions within the framework.

Across 300, 500, and 700 K, the averaged probability densities of the φ_C–Fe–Fe–N_ dihedral angles for Li^+^ ions demonstrate broadened distributions (Figure 4c), illustrating larger octahedral tilting angles and distorted Fe–N bonds. In contrast, Na^+^ ions exhibit narrower distributions of probability densities for the averaged φ_C–Fe–Fe–N_ dihedral angles from φ_*xy*_, φ_*xz*_, and φ_*yz*_ angles (Figure 4d). In comparison to Li^+^ ions, for Na^+^ ions, the framework demonstrates octahedral tilting with smaller angles and less distorted character. For instance, at 300 K, the probability density of the θ_N–Fe–N_ angle for Li^+^ ions shows wider distributions toward smaller angles, compared with those for Na^+^ and K^+^ ions (Figure S25). This result highlights the narrowed cage faces due to Fe–N bond distortions induced by Li^+^ ions, resulting in a potential energy landscape with deep basins, while rigid square cage faces for Na^+^ ions provide a shallower potential energy landscape (smaller *E*_a_^MD^).

A large octahedral tilting leads to significant distortions of crystal structures, resulting in a potential energy landscape with deep basins. This can be attributed to the delicate balance between the Coulombic attractions from CN^–^ anions and the ionic radius of A^+^ ions, which induces the differences in dynamical octahedral tilting angles. Notably, this trend resembles the correlation between the tilting angle and the *E*_a_ observed in solid electrolytes with perovskite structures, such as Li_*x*_La_2/3‑*x*/3_TiO_3_.^57,58^

### 3.4 Framework’s Distortions for Barrier TH Positions via DFT Geometry Optimizations

For Li^+^ and Na^+^ ions, we find that the φ_C–Fe–Fe–N_ dihedral angles and θ_N–Fe–N_ angles formed two perpendicular Fe_N_–N bonds as the main descriptors of framework dynamics. Taking the TH positions as the main barrier occupation position (Table 1), we elucidate the barrier positions in relation to the framework’s distortions. We analyze the minimum sizes for the θ_N–Fe–N_ angles and three φ_*xy*_, φ_*xz*_, and φ_*yz*_ (specific location shown in the inset figure in Figure 4c) as octahedral tilting angles.

When four Li^+^ and Na^+^ ions occupy the TH positions, distinct distortions of the nearest Fe–N bonds and octahedral tilting occur, despite the different variations induced by Li^+^ and Na^+^ ions. Figure 5 shows that, for Li^+^ ions, the closest Fe–N bonds exhibit significant distortions directed toward the Li^+^ ions, whereas, for Na^+^ ions, these distortions are smaller and oriented toward the Na^+^ ions (min­(θ_N–Fe–N_) = 82 and 87° for Li^+^ and Na^+^ ions, respectively, as shown in Table S1). Li^+^ ions are more likely to induce local distortions in the closest Fe–N bonds, whereas the less distorted Fe–N bonds for Na^+^ ions can be contributed to Pauli repulsions.

**Figure 5:**
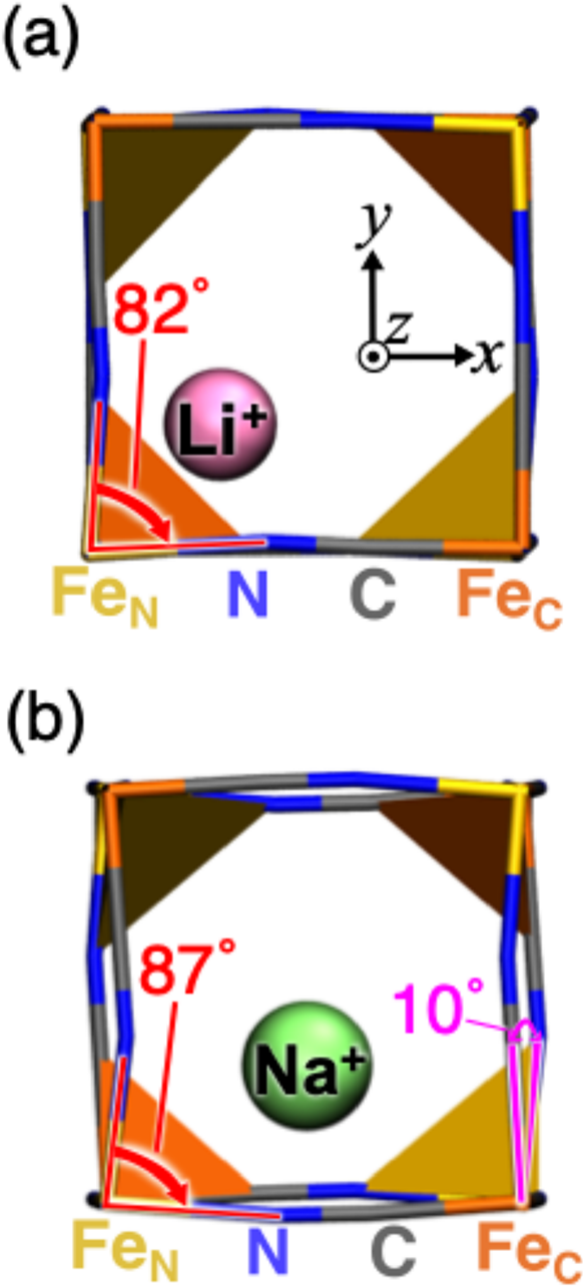
Side views of 1 × 1 × 2 cages from the *z*-direction of the barrier TH positions occupied by Li^+^ ions (pink sphere) and Na^+^ ions (lime sphere). Red lines and arrows show the angles that formed two perpendicular Fe_N_–N bonds. Pink lines and arrows show the C–Fe_C_–Fe_N_–N dihedral angle (φ_C–Fe–Fe–N_) defined in the *xy*- (φ_*xz*_, defined in gray lines in the inset figure in Figure 4c). The blue, gray, and yellow (orange) lines represent N atoms, C atoms, and the Fe ions coordinating with N (C) atoms, respectively. The Fe ions coordinating with the N (C) atoms are in a sextet (singlet) spin state with +3 (+2) valence. Note that, to clearly display the framework’s distortions and the occupation positions of the Li^+^ and the Na^+^ ions, we show the 1 × 1 × 2 cages. To clearly show the octahedral tilting, we added the polyhedra of Fe ions.

Additionally, both Li^+^ and Na^+^ ions exhibit a framework characterized by *a* ^–^ *a* ^–^ *a* ^–^ octahedral tilting, as denoted by Glazer (see Figure 5a,b; φ_*yz*_, φ_*xz*_, and φ_*xy*_ in Table S1). Notably, for Na^+^ ions, the framework undergoes substantial octahedral tilting (Figures S3g and S4g). In fact, for Na^+^ ions, the φ_*yz*_ angle is significantly smaller than the φ_*xz*_ and the φ_*xy*_ angles (φ_*yz*_, φ_*xz*_, and φ_*xy*_ = 1.1, 7.7, and 10̊, respectively, as listed in Table S1). These findings suggest that Li^+^ ions primarily induce local distortions in the closest Fe–N bonds, whereas Na^+^ ions are more likely to induce octahedral tilting. Further discussion on the relationship between (off-)­FC and BC occupation positions and framework distortion is provided in Section S8.

### 3.5 Diffusion Pathways, Activation Energies, and Framework Distortions via DFT-NEB Analysis

It has previously been demonstrated that Na^+^ ions have smaller *E*_a_^MD^ than Li^+^ ions and K^+^ ions have negligible D*. To investigate the framework distortions induced by the cooperative transport of A^+^ ions in relation to their impact on the characteristics of *E*_a_, we utilize the NEB method to analyze the migration characteristics of the A^+^ ions. We compare the *E*_a_^NEB^ of the predefined diffusion pathway of the concerted mode (Figure 6) as well as the conventional single ion hopping mode (Figure S26). We take the θ_N–Fe–N_ angles as the main framework distortion by A^+^ ion positions for either diffusion mode. We calculate the θ_N–Fe–N_ angles on the *xy-*, and *yz-*planes, using the first- and second closest N atoms and the first closest Fe_N_ ion from A^+^ ions. To the best of our knowledge, this study is the first to accurately estimate *E*_a_ by incorporating cooperative diffusion effects within NEB calculations through the creation of a concerted mode.

**Figure 6:**
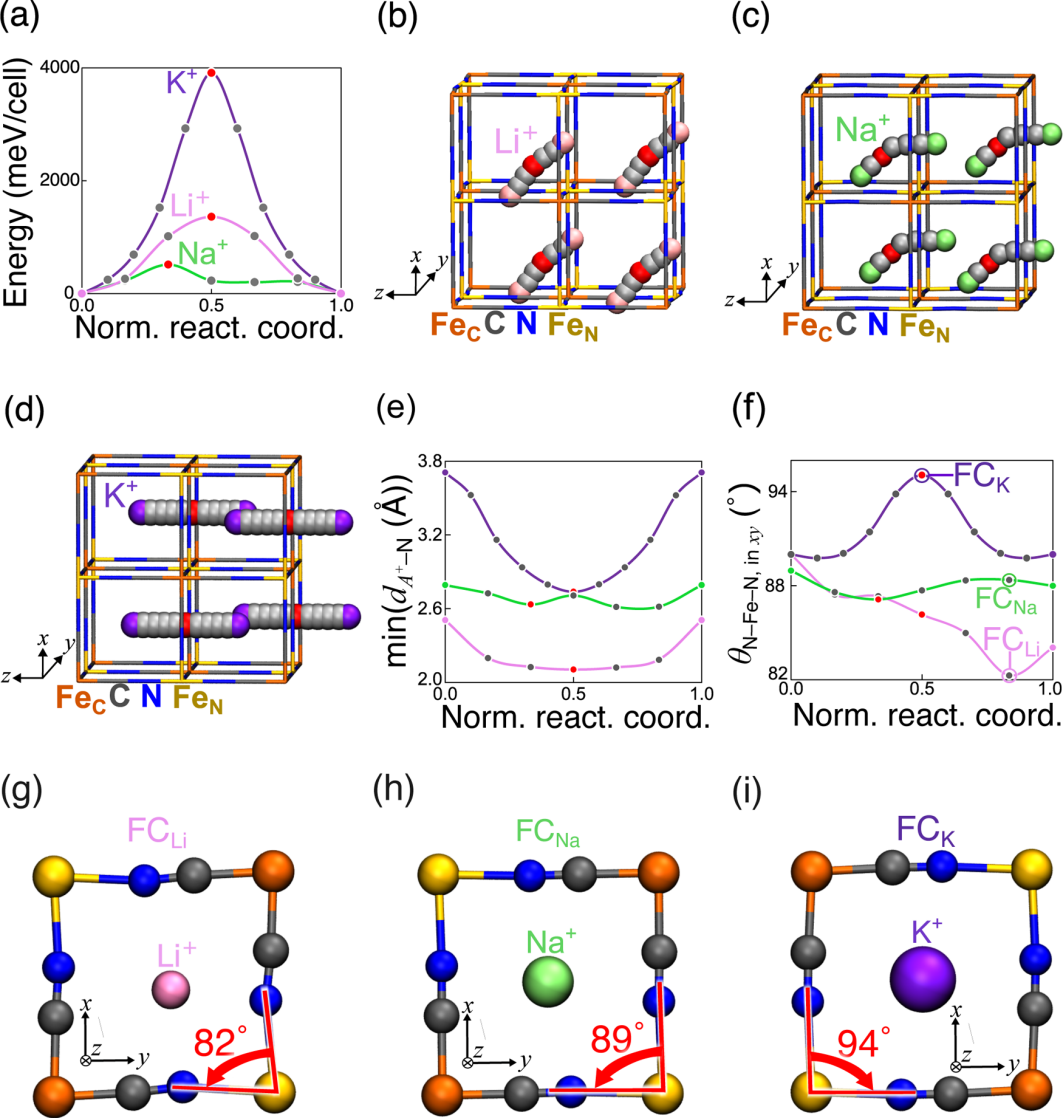
Concerted mode diffusion pathways between the most stable occupation positions for A^+^ ions. (a) The potential energy profiles and (b–d) their diffusion pathways of (b) Li^+^, (c) Na^+^, and (d) K^+^ ions (the pink, green, and purple spheres correspond to the FC, the off-FC, and the BC positions, respectively). (e) Minimum distance between the A^+^ ion and the N atoms (min­(*d*_A^+^–N_)). In (b–d) panels, the red spheres correspond to barrier geometries (corresponding energies represented as the red points in panel (a)). (f) The first and second closest N atoms and the first closest Fe_N_ ion from A^+^ ions on the *xy*-plane (θ_N–Fe–N, in *xy*_). In (g–i), the local framework distortions of the occupied FC positions by the A^+^ ion (corresponding geometries are marked with an empty cycle with ″FC_*A*_″ labels). Note that in parts (b–d), we visualize the blue, gray, yellow, and orange lines indicate N atoms, C atoms, the Fe ions coordinating with the N atom, and the Fe ions coordinating with the C atom to clearly display the pathways. We designated the label “Norm. react. coord.” meaning normalized reaction coordinate.

For the concerted mode, Li^+^ and Na^+^ ions require significantly smaller net *E*_a_ per A^+^ ion than that of the single-ion hopping mode. In fact, Li^+^ and Na^+^ ions exhibit *E*_a_^NEB^ values of 1329 meV/cell (332 meV per ion) and 514 meV/cell (129 meV per ion), respectively (Figure 6a). In contrast, for a single ion hopping mode, a Li^+^ and a Na^+^ ion exhibit *E*_a_^NEB^ of 761 meV/ion and 414 meV/ion, respectively (Figure S26a). These high *E*_a_^NEB^ can be attributed to the Coulombic repulsions from the two other closest A^+^ ions, where the A^+^–A^+^ distance is 4.74 Å, particularly, in the case of the A^+^ ion at the least stable geometry (red spheres in Figure S26b,d). Note that we intentionally modeled an asymmetric migration pathway^59^ to better reflect the realistic energy landscape. This is because, based on our structural optimization, both the TH and FC positions act as energy barriers.

During the migration of Li^+^ ions, they induce more pronounced distortions in the Fe–N bonds, resulting in a higher *E*_a_ compared to Na^+^ ions. For both modes, Li^+^ (Na^+^) ions have a curved pathway connecting from the FC (off-FC) on the *yz*-plane to the FC (off-FC) positions on the *xy*-plane (Figures 6b,c and S26b,c). Notably, the presence of Li^+^ ions leads to a significant decrease in the θ_N–Fe–N_ angle in the *xy*-plane (pink line in Figure 6f), compared to that of Na^+^ ions (green line in Figure 6f). Hence, the cage faces are distorted due to the proximity of Li^+^ ions (Figure 6g), while Na^+^ ions induce a less distorted framework (Figure 6h), resulting in Na^+^ ions having smaller *E*_a_^NEB^ than Li^+^ ions. As we expected, Li^+^ ions take shorter Li^+^–N distances than those of Na^+^ ions (pink and green line in Figure 6e), leading to the weakening of the Fe_N_–N bonds due to the closer proximity of A^+^ ions to N atoms.

Meanwhile, for both modes, K^+^ ions take a linear pathway connecting the BC positions (Figures 6d and S26d), with high *E*_a_^NEB^ of 3910 meV/cell (978 meV per ion, Figure 6a) and 874 meV/ion (Figure S26a) for concerted and single-ion hopping modes, respectively. The present result supports the conclusion derived from our DFT-MD results, which shows that K^+^ diffusion does not occur within anhydrous pristine Fe-based PB crystals. This can be rationalized by the fact that the larger K^+^ ions induce a positive variation in the θ_N–Fe–N_ angles (red point in Figures 6f and S27b), when K^+^ ions approach the FC positions (red spheres in Figures 6d and S26d). Hence, in both modes, the larger energy from the distorted Fe–N bonds contributes to higher *E*_a_^NEB^ values than those of Li^+^ and Na^+^ ions. This finding demonstrates that K^+^ ions’ occupancy at FC positions leads to distorted Fe–N bonds outward. These distortions are clearly attributed to electronic orbital repulsions between K^+^ ions and CN^–^ ligands (Figure 6i), due to a larger radius than that of Li^+^ and Na^+^ ions.

Our previous quantum-chemical DFT calculations, utilizing local (atom-centered Gaussian) basis sets, reveal the *E*_a_ of a single Li^+^ ion and a single K^+^ ion in a single Prussian White (reduced PB) cage. Our earlier work demonstrates that a single Li^+^ ion takes diffusion pathways between the nearest neighboring FC positions with a small *E*_a_ of 169 meV, whereas a single K^+^ ion follows pathways connecting between the BC positions with a high *E*_a_ of 1131 meV.^29^ The difference of *E*_a_ in our earlier work is attributed to neglecting distortions of Fe–N bonds induced by A^+^ ions and A^+^–A^+^ Coulombic repulsions. Our DFT-NEB results demonstrate that the *E*_a_^NEB^s rank in the order Na^+^ < Li^+^ ≪ K^+^. Additionally, for Li^+^ and Na^+^ ions, the net *E*_a_^NEB^s for concerted modes are lower than those of the single ion hopping modes. Importantly, for Li^+^ and Na^+^ ions, the *E*_a_^MD^ values are substantially lower than the *E*_a_^NEB^s for concerted modes. Hence, our DFT-NEB results strengthen the reliability of our DFT-MD results.

Electrochemical impedance spectroscopy (EIS) measurements on *M*Fe­(CN)_6_ crystals reported that the *E*_a_ for Na^+^ diffusion is lower than that for Li^+^ ions. For instance, in MnFe­(CN)_6_ films with a lattice length of 10.56 Å, the *E*_a_ values are 480 and 220 meV for Li^+^ and Na^+^ ions, respectively. In CdFe­(CN)_6_ films, with a lattice length of 10.7 Å, the *E*_a_ values are 250 and 120 meV for Li^+^ and Na^+^ ions.^60^ Hence, the balance between the lattice size and the size of the inserted A^+^-ion is a critical factor influencing *E*_a_. Consequently, PBA with a lattice length longer than that of PB, such as Mn_4_[Mn­(CN)_6_]_4_, may exhibit significantly lower *E*_a_ for Na^+^.

### 3.6 K^+^ Ions’ Self-Diffusion Coefficients in Defective Framework via DFT-MD Calculations

The previous EIS study reported that the *E*_a_ for K^+^ ions is 647 meV in a PBA film containing Mn and anion vacancies, with an elemental ratio of K/Mn/Fe = 1.81:1:0.96:0.04.^61^ Notably, previous DFT-NEB studies reveal that K^+^ ions exhibit a lower *E*_a_^NEB^ in anion-defective frameworks (800 meV), compared to pristine frameworks (1200 meV).^11,25^ These experimental and computational results, along with our DFT calculations for the pristine crystal, indicate that anion vacancies significantly enhance the K^+^ ion diffusion in PB crystals.

By expanding the 4 × 2 × 2 cages and incorporating two anion defects (i.e., [Fe­(NC)_5_]^3–^ and [Fe­(CN)_5_]^3–^), we generated a one-dimensional vacancy channel along the *z*-direction (Figure 7a). We analyze the D* over a 20 ps production run at temperatures of 700, 800, and 1000 K. At 700 K, K^+^ ions exhibit the effective D* exclusively along the *z*-direction, demonstrating anisotropic diffusion behavior along the anion vacancy channels (Figure 7b). As the temperature increased to 800 K, K^+^ ions demonstrate the effective D* in both the *x*- and *z*-directions, while diffusion in the *y*-direction remains negligible. These results indicate that K^+^ ions exhibit anisotropic diffusions at relatively low temperatures, transitioning to pseudoisotropic behavior at elevated temperatures. Therefore, regulating the anion defect density can optimize K^+^ ion self-diffusion in PB/PBA crystals.

**Figure 7:**
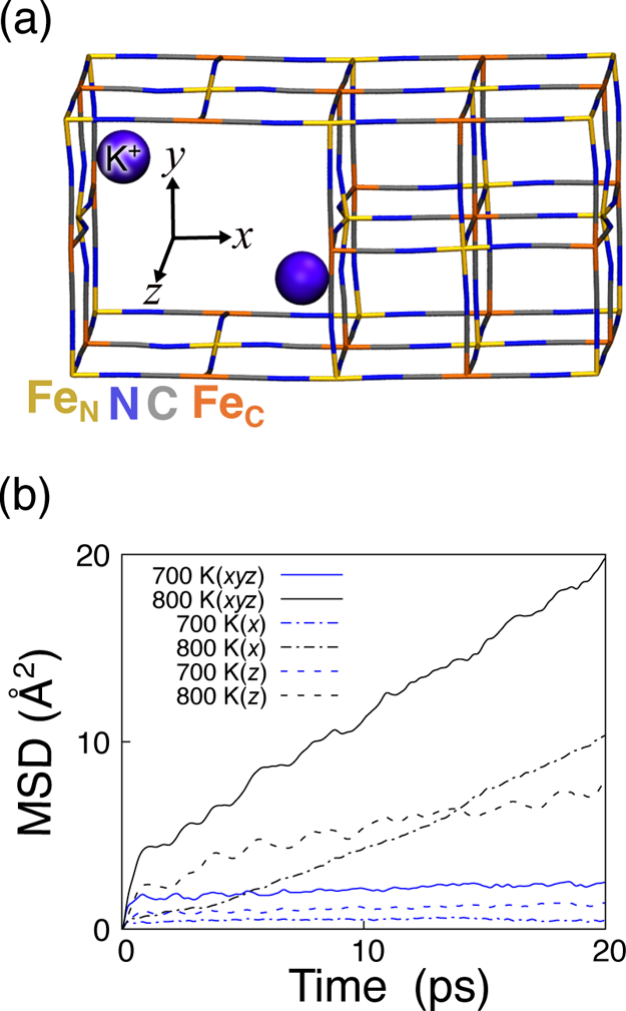
(a) Side view of 4 × 2 × 2 cages depicting K^+^ ions occupying stable positions within the anion vacancy channel. (b) MSDs at temperatures of 700 K (blue lines) and 800 K (black lines). The subscript letters associated with the Fe ions indicate the corresponding sides of coordination with the CN^–^ ligands. The purple, blue, gray, and yellow (orange) spheres represent K^+^ ions, N atoms, C atoms, and Fe ions, coordinated with N (C) atoms, respectively. For our analysis, we have excluded the first 10 ps of the MD simulations to ensure the system has reached equilibrium.

We emphasize that not only anionic defect density but also spin polarization influences the stability of electronic structures at high temperatures and the decomposition temperature of PB crystals. At high temperatures, the increase in anionic vacancy density may concurrently compromise the thermal stability, posing a risk to the safe application of KIBs. Specifically, at 1000 K in our simulations, the defective cages begin to decompose, generating nitrile compounds such as cyanogen, while the pristine framework K^+^ ions remain stable.

Furthermore, for A^+^ ions, the frameworks undergo decomposition alongside unsuccessful SCF convergence during DFT-MD calculations without spin polarization at 1000 K. This temperature can be regarded as the decomposition temperature of PB crystals in our calculation condition, though the corresponding experimental value is 503 K.^62,63^ Nonetheless, the demonstration underscores the importance of safety considerations when employing PB cathodes in NIB and KIB applications, particularly under high-temperature conditions. Thereof, to ensure reliable comparisons of A^+^ ion self-diffusivities in PB systems, DFT-MD simulations should be employed at *T* < 1000 K.

At high temperature, the D* values for Li^+^ and Na^+^ ions, generated by DFT-MD calculations with and without spin polarization, converge to similar values. However, at room temperature, they display significant differences (Figure S24a). This finding indicates that extrapolating D* at room temperature from high-temperature DFT-MD calculations is not reliable, particularly for cathode materials. Therefore, DFT-MD calculations with spin polarization at 300 K should be employed to accurately estimate D* at room temperature.

Our DFT-MD simulations reveal surface-like diffusion pathways for Li^+^ and Na^+^ ions, which persist even under framework dynamics.^63^ In fully ordered PBAs, both ions do not follow migration pathways connecting the conventional BC positions. While prior studies often explain site stability and activation barriers based on coordination numbers, they tend to overlook ion–ion interactions. Our results and recent findings show that Li^+^ ions prefer TH positions over FC ones in the Li_1_Fe_4_[Fe­(CN)_6_]_4_ model, emphasizing the role of A^+^–A^+^ interactions in stabilizing occupation positions.^64^ In addition, A^+^–CN^–^ ligand interactions—such as Coulomb attraction and steric repulsions—strongly influence both site stability and *E*_a_.^65^ Together, our results highlight that fast ion diffusion in porous frameworks depends not only on coordination environments but also on interionic and ion–framework interactions. Moreover, consistent with earlier reports,^59,66^ we note that cage size and ionic size govern the topology of surface-like diffusion pathway and the height of *E*_a_. Hence, in PB-type materials, static framework distortion (e.g., via Jahn–Teller-active metals) can be expected to promote anisotropic, fast diffusion along larger cage faces.

## 4 Conclusions

This study comprehensively compares the diffusion mechanisms of multiple Li^+^, Na^+^, and K^+^ ions, using DFT calculations. Our key findings are as follows: (1) Na^+^ ions have high self-diffusion coefficients at room temperature with small activation energies, concluding that anhydrous pristine Fe-based PB crystals serve as excellent Na^+^ conductors at room temperature. (2) Contrary to conventional BC positions and previous theoretical findings, Li^+^ and Na^+^ ions preferentially occupy FC and off-FC positions, respectively. (3) At high temperatures, Li^+^ diffusivity is significantly higher than that of Na^+^ ions. Additionally, (4) Na^+^ diffusivity becomes comparable to Li^+^ around room temperature, owing to the lower level of octahedral tilting of the PB framework in the Na^+^ case. This can be rationalized by the larger ionic radius of Na^+^ ions, resulting in weaker Coulombic attractions from the framework, compared to that of Li^+^ ions.

In the K^+^ case, (5) K^+^ ions’ diffusivity in pristine crystal remains nearly zero, while the presence of anionic defects within the PB framework is essential for achieving nonzero diffusivity. (6) K^+^ ions follow anisotropic diffusion pathways in the defective crystal, while Li^+^ and Na^+^ ions take isotropic pathways in the pristine crystal. This finding revises the conventional assumption based on Stokes’ ionic radius; the pristine PB crystal was expected to be a good conductor of K^+^ ions.

This study establishes a correlation between diffusivities and factors, such as ionic radius, electronic orbital interactions between A^+^ ions and the CN ligands, and framework distortion within MOF materials. These findings facilitate the understanding of fundamental chemistry in MOFs and advance the development of next-generation batteries and catalysts.

## Supplementary Material



## Notes

Detail of the point charge of EwaldSolidSolution; all probable occupation sites in the unit cell; the total energy versus cell length; the optimized structures for the occupation ((off-)­FC, TH, and BC) positions for Li^+^ and Na^+^ ions; the comparison of the electronic and geometric parameters; validation of the stability of occupation positions; statistical validation of Arrhenius plot; the diffusion pathway at 700 K; the RDFs between Li^+^ and Na^+^; and K^+^ ions and framework’s atoms (C, N, and Fe), the hopping mechanism of A^+^ ions; validation of MSD and Arrhenius plots for Li^+^ and Na^+^ ions under different DFT calculation conditions; probability densities for the N–Fe_N_–N dihedral angles; and DFT-NEB for the single ion hopping modes (PDF)
