# Supplementary material for: Dissimilar Diffusion Mechanisms of Li+, Na+, and K+ Ions in Anhydrous Fe-Based Prussian Blue Cathode
Source: J Am Chem Soc. 2025 Jun 30;147(29):25441–53. doi: 10.1021/jacs.5c05274 (PMC12291462; doi:10.1021/jacs.5c05274)
Supplement: Supplementary file 1 [file ja5c05274_si_001.pdf]

Supporting Information for

Dissimilar Diffusion Mechanisms of Li^+, Na^+, and K^+ Ions in Anhydrous Fe-Based Prussian Blue Cathode

Dan Ito[†,¶,§,*], *Seong-Hoon Jang*[¶,fl], *Hideo Ando*[$], *Toshiyuki Momma*[†],

and Yoshitaka Tateyama[†,¶,§,*]

[†]Graduate School of Advanced Science and Engineering, Waseda University, 3-4-1, Okubo, Shinjuku-ku, Tokyo 169-8555, Japan

[¶]Research Center for Energy and Environmental Materials (GREEN), National Institute for Materials Science (NIMS), 1-1 Namiki, Tsukuba, Ibaraki 305-0044, Japan

[§]Laboratory for Chemistry and Life Science, Institute of Science Tokyo, 4259 Nagatsuta-cho, Midori-ku, Yokohama, Kanagawa 226-8501, Japan

[fl]Institute for Materials Research, Tohoku University, 2-1-1 Katahira, Aoba-ku, Sendai, Miyagi 980-8577, Japan

[$]Faculty of Science, Yamagata University, 1-4-12 Kojirakawa-machi, Yamagata-shi, Yamagata 990-8560, Japan

E-mail: danito@fuji.waseda.jp; tateyama@cls.iir.isct.ac.jp

S1. All Possible Occupation Sites for A^+ Ions and the Detail Local Charges for EwaldSolidSolution

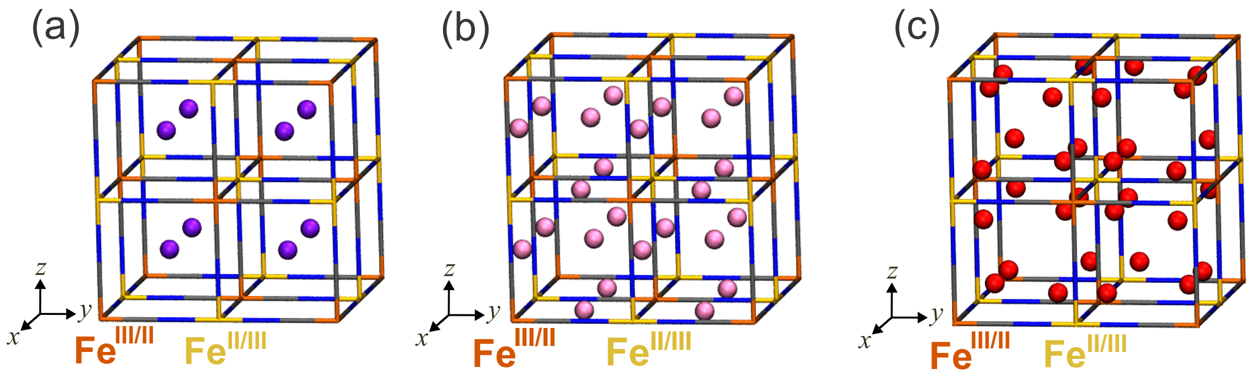

Figure S1. All possible occupation sites in the PB unit cell for the Wyckoff (a) 8c (BC), (b) 24d (FC), and (c) 32f (TH) sites. The spheres representing the framework's atoms (C, N, and Fe) are not shown for the clarification. The blue, gray, and yellow (orange) lines indicate the N atoms, the C atoms, the Fe ions coordinating with the N (C) atoms, respectively.

The dipole moments of CN were reported as 0.5 Debye, [S1] then we determined the charge of the dipole as $\delta q = 0.0887\ e$. By distributing the charge –0.5 e to C and N atoms to reproduce CN^- anion, we determined that $q_c = -0.5 + \delta q = -0.4113\ e$, and $q_N = -(0.5 + \delta q) = -0.5889\ e$.

To confirm the success of our site arrangement screening, we check the correlation between Ewald energy and DFT energies -- in the DFT calculations, four A^+ ions are replaced by four Li^+ ions and compared with the energies explored for 8c (BC), 24d (FC), and 32f (TH) Wyckoff sites. Using the 8c (BC) Wyckoff site as the representative case, we randomly select six different site arrangements with different Ewald energy (not energetically identical) and we confirm a good positive correlation (We find $R^2 = 0.7$).

S2. Stability of Occupation Positions and their Geometric and Electronic Structures via DFT Structural Optimizations

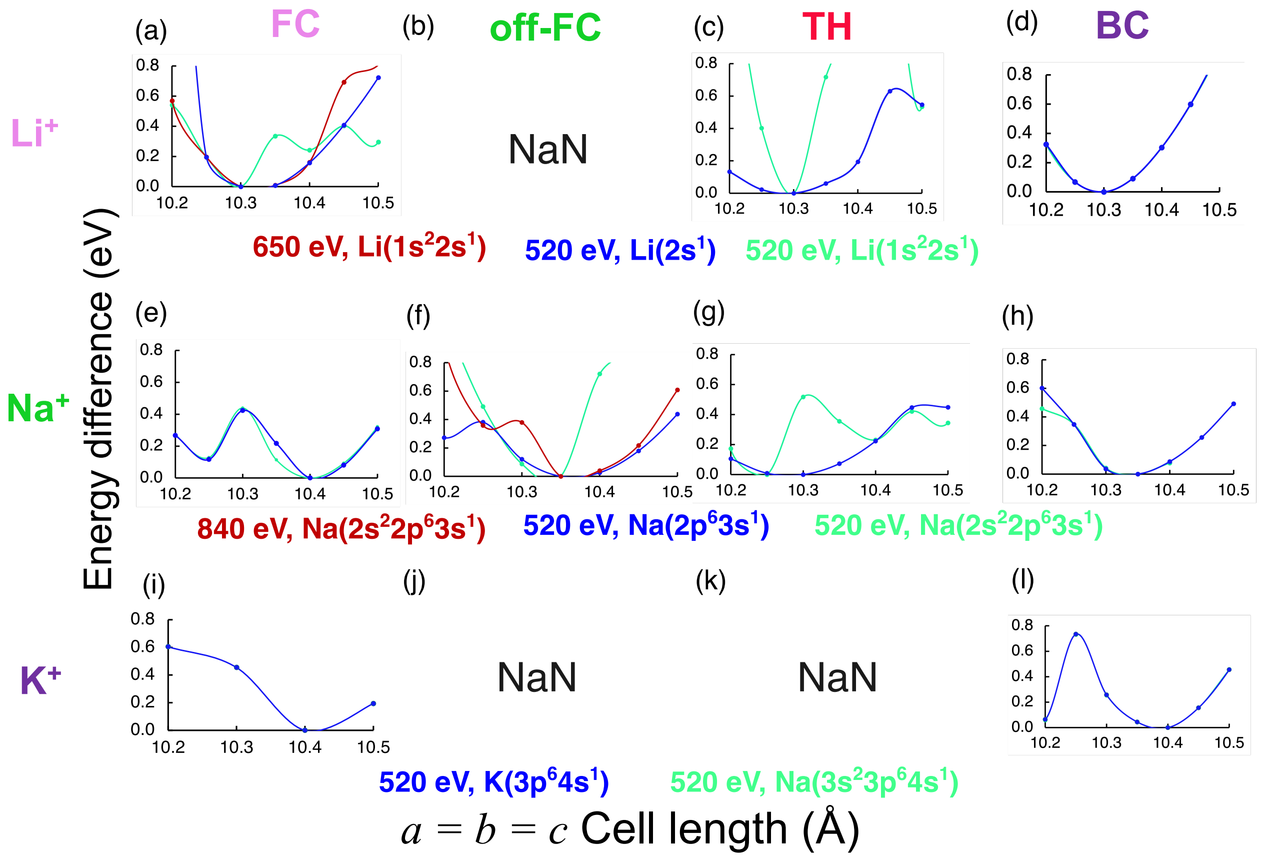

Figure S2. Comparison of the total energy *vs.* cell length of cubic framework with the A^+ ions (A^+ = Li^+, Na^+, and K^+) occupying the occupations ((off-)FC, TH, and BC) positions, by employing the different DFT calculation conditions. We change the PAW for the A^+ ions, and cutoff energy. The red lines correspond the results obtained with high cutoff energy and more considered electrons ($1s^22s^1$ and 650 eV cutoff energy for Li^+ ions, $2s^22p^63s^1$ and 840 eV cutoff energy for Na^+ ions, and $3s^23p^64s^1$ and 520 eV cutoff energy for K^+ ions). The green lines correspond the results obtained with 520 eV cutoff energy and considered more electrons for Li^+ and Na^+ ions. The blue lines correspond that the results with 520 eV cutoff energy and less considered electrons ($2s^1$ for Li^+ ions, $2p^63s^1$ for Na^+ ions, and $3p^64s^1$ for K^+ ions).

Supporting Discussion

The cell parameter and voltage profiles of experimental values are typical parameters to the compare computational results. We calculated the voltage profile, V, using the following formula:

$$V = -\frac{[E(A_4\mathrm{Fe}_4[\mathrm{Fe(CN)}_6]_4) - E(\mathrm{Fe}_4[\mathrm{Fe(CN)}_6]_4) - 4E(A)]}{4} \qquad \text{(S1)}$$

, where $E(A_4\mathrm{Fe}_4[\mathrm{Fe(CN)}_6]_4)$ is the total energy of the system containing the four A^+ ions, $E(\mathrm{Fe}_4[\mathrm{Fe(CN)}_6]_4)$ is the total energy of the system without four A^+ ions, and $E(A)$ is the energy per A^+ ion in the bulk bcc A (A = Li, Na, and K) metal.

We find that our calculations overestimate the cell lengths within 0.22 Å range (Table S1). These errors can be explained by that PBE functional generally overestimate the cell lengths. Our estimated voltage for the Li^+ ion is good agreements with the experimental value, while the values for Na^+, and K^+ ions underestimate the 0.33, and 0.54 V, respectively. These differences can be explained by the impurities of crystal, such as water molecules and anions defects.

It is worth examining the electronic states such as the on-site local magnetic moments, μ, for better understanding of valence states of the Fe ions, depending on the location of the occupation position, and the size of the inserted A^+ ions. For any A^+ ion and occupation position, the on-site local magnetic moments of the Fe ions (μ_{Fe}) coordinating with N ($\mu_{\mathrm{Fe_N}}$) and with C ($\mu_{\mathrm{Fe_C}}$) atoms are 4.33(5), and 0.16(7) μ_B, respectively (Tables S1). Given the delocalized nature of the electrons of the Fe ions, the charges of Fe_N and Fe_C approximately correspond +3 in the sextet spin configuration, and +2 in the singlet spin configuration, respectively. Therefore, the valence state of the Fe ions does not depend on the size of the inserted A^+ ions and the locations of the occupation positions.

Table S1. The electronic and geometric parameters of the geometries for the A^+ ions occupying the stable occupation ((off-)FC, TH, and BC) positions. μ represents the local magnetic moment. Three C–Fe–Fe–N dihedral angles (specific location of φ_{xy}, φ_{xz}, and φ_{yz} in the inset figure in Figure 4c) are used to obtain octahedral tilting mode, as denoted by Glazer. We took the experimental reference for the voltage profiles, and cell parameters from the stoichiometric compounds as $Li_{1.05}Fe[Fe(CN)_6]_{0.97}\square_{0.03}\cdot 2.6H_2O$,[S2] $Na_{0.61}Fe[Fe(CN)_6]_{0.94}\square_{0.06}$,[S3] and $KFeFe(CN)_6$.[S4]

A^+	Li^+			Na^+				K^+	
Occupation position	FC	BC	TH	FC	BC	TH	off-FC	FC	BC
C–N (Å)	1.176	1.176	1.177	1.176	1.177	1.177	1.176	1.178	1.177
Ref. C–N (Å)				1.13[S5]					
C–Fe_C (Å)	1.883	1.890	1.897	1.903	1.907	1.899	1.893	1.922	1.906
Ref. C–Fe_C (Å)				1.92[S5]					
N–Fe_N (Å)	2.096	2.085	2.084	2.121	2.091	2.076	2.106	2.105	2.117
Ref. N–Fe_N (Å)				2.03[S5]					
A–*A* (Å)	7.283	7.283	7.283	7.354	7.319	7.283	7.314	7.354	7.354
A–Fe_C (Å)	3.642	4.46	4.206	3.677	4.482	4.195	3.807	3.677	4.503
A–Fe_N (Å)	3.642	4.46	2.708	3.677	4.482	3.395	3.812	3.67	4.503
A–C (Å)	2.649	3.706	2.732	2.713	3.722	3.031	2.888	2.783	3.742
A–N (Å)	2.506	3.674	2.09	2.608	3.693	2.686	2.785	2.744	3.709
min(N–Fe_N–N) (˚)	84	90	82	88	90	87	88	95	90
min(C–Fe_C–C) (˚)	89	90	90	89	90	90	89	96	90
φ_{yz} (˚)	3.6	0	3.6	0.4	0	1.1	0.7	5.6	0
φ_{xz} (˚)	0	0	0.9	0	0	7.7	1.1	0	0
φ_{xy} (˚)	0	0	4	0	0	10	1.1	0	0
Glazer Notatiton	$a^-a^0a^0$	$a^0a^0a^0$	$a^-a^-a^-$	$a^-a^0a^0$	$a^0a^0a^0$	$a^-a^-a^-$	$a^-a^-a^-$	$a^-a^0a^0$	$a^0a^0a^0$
Space group	*I*mmm	$F\bar{4}3$m	*C*m	*I*mmm	$F\bar{4}3$m	*C*m	*P*1	*I*mmm	$F\bar{4}3$m
Ref. Space group				$Fm\bar{3}m$[S5]					
μ_{Fe_N}(μ_B)	4.35	4.35	4.33	4.35	4.35	4.34	4.35	4.34	4.35
μ_{Fe_C} (μ_B)	0.16	0.16	0.17	0.17	0.17	0.16	0.17	0.17	0.17
Ref. μ_{Fe_N} (μ_B)				5.02[S6]					
Voltage (V)	3.14	-	-	-	-	-	2.57	3.26	4.24
Ref. Voltage (V)		3.1[S2]			2.9[S3]			3.7[S4]	
Cell Length (Å)	10.3	10.3	10.3	10.4	10.35	10.3	10.35	10.4	10.4
Ref. Length (Å)		10.23[S3]			10.22[S4]			-	

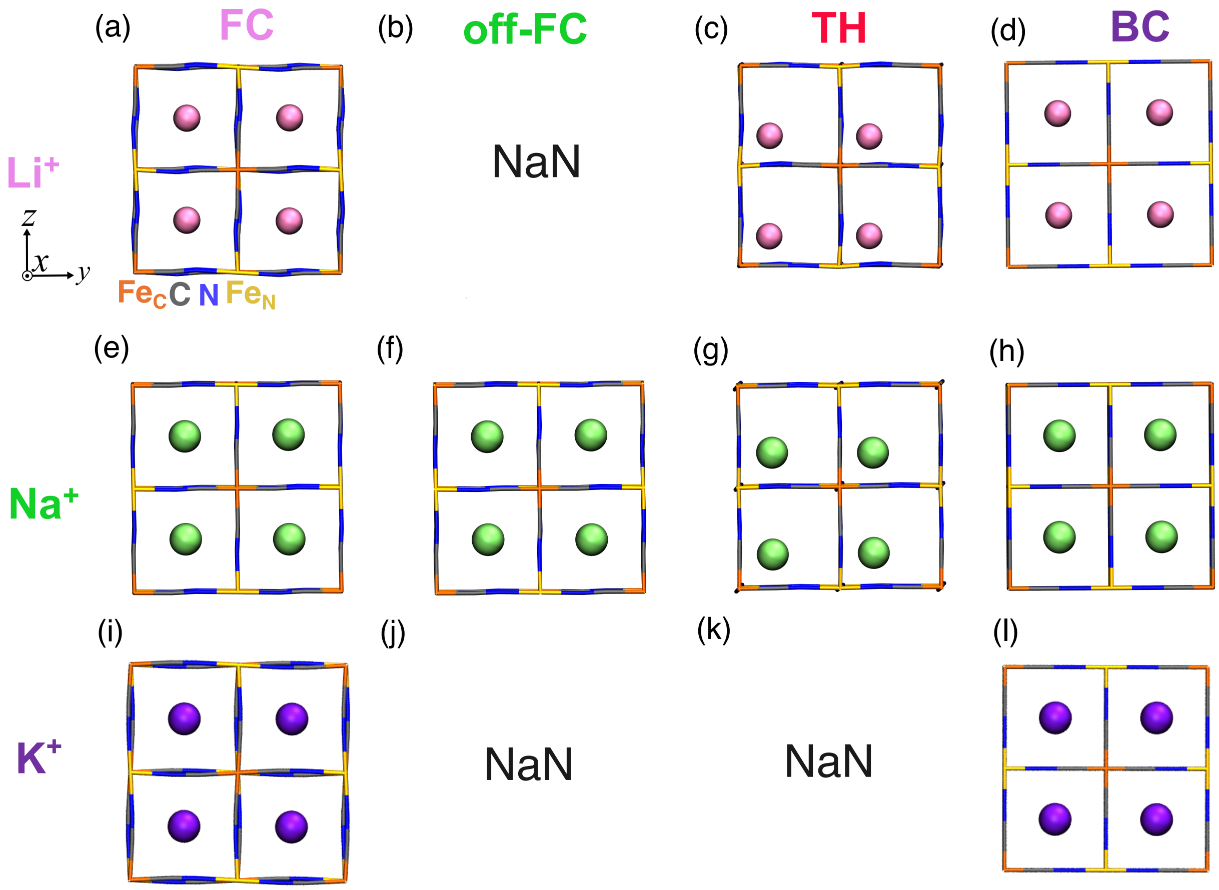

Figure S3. Side view from *x*-direction of Li^+, Na^+, and K^+ ions occupying the stable occupation ((off-)FC, TH, and BC) positions. The blue, gray, yellow (orange), lines represent the N atom, C atom, the Fe ion-coordinating with N (C) atom, respectively. The Fe ions coordinating with the N (C) are in sextet (singlet) spin state with +3 (+2) valence. The pink, lime, and purple spheres illustrate Li^+, Na^+, and K^+ ions, respectively.

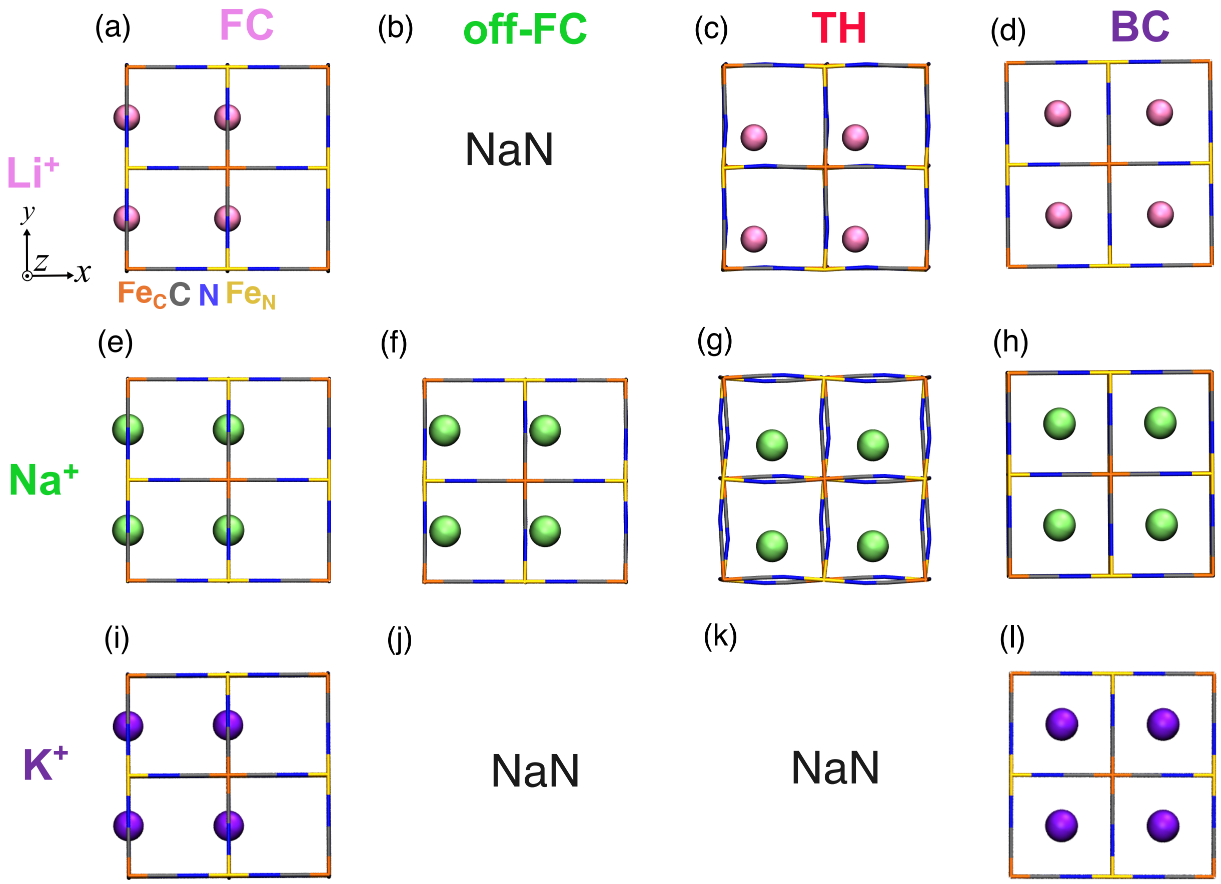

Figure S4. Side view from *z*-direction of Li^+, Na^+, and K^+ ions occupying the stable occupation ((off-)FC, TH, and BC) positions. The blue, gray, yellow (orange), lines represent the N atom, C atom, the Fe ion-coordinating with N (C) atom, respectively. The Fe ions coordinating with the N (C) are in sextet (singlet) spin state with +3 (+2) valence. The pink, lime, and purple spheres illustrate Li^+, Na^+, and K^+ ions, respectively.

S3. Validation of the Stability of Occupation Positions for A^+ Ions via DFT Calculations

To find the computationally reasonable SCF condition for our DFT-MD calculations, we evaluated the potential energy landscape (PEL) versus cell length by using different SCF conditions, including the PAW for A^+ ions and cutoff energies.

For either A^+ ions occupying the BC positions, their PELs are almost identical for different choice of the PAW for the A^+ ion and cutoff energy. It can be explained by that the weakest Coulombic interaction between the A^+ ion and the framework in the cage because the BC position is the further position from the framework in the cage.

Li^+ and Na^+ ions occupying the (off-)FC and TH positions have the different PELs depending on the selection of the PAW for the A^+ ion and cutoff energy (Figure S2). The PELs obtained with a smaller number of considered electrons for the A^+ ions and 520 eV cutoff energy show the parabolic shapes (blue lines in Figure S2). In contrast, the PELs obtained with a larger number of considered electrons for the A^+ ions and 520 eV cutoff energy show non-parabolic shapes (light green lines in Figure S2). Hence, when Li^+ and Na^+ ions occupy the (off-)FC and TH positions, the PELs strongly depend on the selection of the PAW for the A^+ ion.

As the reference results for the non-parabolic PELs obtained with a larger number of considered electrons for Li^+ and Na^+ ions, we compare the PELs obtained with high cutoff energy (red lines in Figure S2). We used 650 and 840 eV cutoff energy for Li^+ and Na^+ ions, respectively. Compared the non-parabolic green lines in Figure S2, the red lines show parabolic shapes, and these local minima take the same minima with the blue lines (a smaller number of considered electrons for the A^+ ions and 520 eV cutoff energy). Therefore, when Li^+ and Na^+ ions occupy the (off-)FC and

TH positions, the PELs strongly depend on the selection of the PAW for the A^+ ion and cutoff energy.

These results illustrate that the localized inner electrons of Li^+ and Na^+ ions relate the PELs when Li^+ and Na^+ ions approach to the framework (*i.e.,* the (off-)FC and TH positions). It can be explained by an electronic orbital distribution. The ionic radius can be related to the localized inner electrons. When we use high cutoff energy, the accurate modeling of the localized nature of the inner electrons of Li^+ and Na^+ ions requires the appropriate combination of the PAW for the A^+ ion and the cutoff energy. Considering the balance of the computational cost and the accuracy of the PELs for our DFT-NEB, and -MD calculations, we selected the PAW for A^+ ions with a smaller number of electrons considered, and a cutoff energy of 520 eV.

Table S2. Comparison of the stability (meV cell^{-1}) of the four different occupation positions ((off-)FC/TH/BC) by using single point calculation for Na^+ ions between different DFT functional. The energy scale is relative to the most stable occupation positions for Na^+ ion. We optimized the cell length and the coordinates of all atoms. We used the structure of the optimized structures from the PBE+U+D3 calculations.

Occupation positions	Na^+ (PBE+U+D3)	Na^+ (PBE+U)	Na^+ (r2SCAN+rVV10)
**FC**	150	0.0	18
**off-FC**	0.0	138	0.0
**TH**	462	378	126
**BC**	432	1464	1566

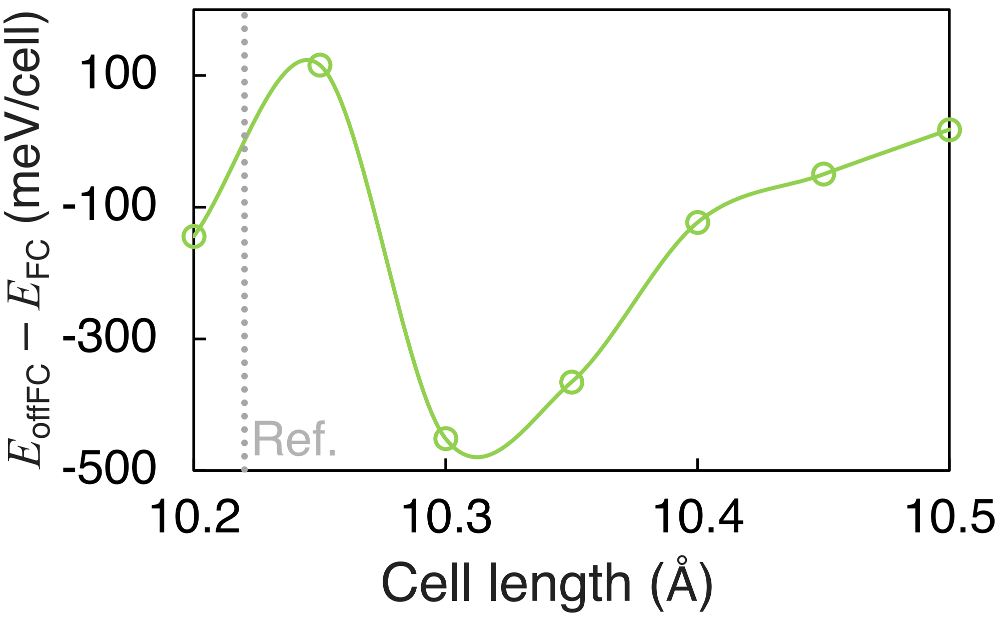

Figure S5. Comparison of the stability energy with Na^+ ions occupying the occupations (off-)FC positions. *vs.* cell length of cubic framework. We used the results with 520 eV cutoff energy and less considered electrons ($2p^6 3s^1$ for Na^+ ions). We took the results from the blue lines in Figure S2e, and S2f. The gray dashed line corresponds the experimental value. [S3]

Table S3. Comparison of the stability (meV $cell^{-1}$) of the four different occupation positions ((off-)FC/TH/BC) for Na^+ ions between different DFT functionals. The energy scale is relative to the most stable occupation positions for each Na^+ ion. We optimized the cell length and the coordinates of all atoms.

Occupation positions	Na^+ (PBE+*U*+D3)	Na^+ (PBE+*U*)	Na^+ (r2SCAN+rVV10)
**FC**	150	6	18
**off-FC**	0.0	0.0	0.0
**TH**	462	576	564
**BC**	432	1686	1752

Table S4. Comparison of the stability (meV cell^{-1}) of the three different occupation positions (FC/TH/BC) for Li^+ ions between different DFT functionals. The energy scale is relative to the most stable occupation positions for each Li^+ ion. We optimized the cell length and the coordinates of all atoms. "Not converged" means that the optimized structure was not the off-FC positions.

Occupation positions	Li^+ (PBE+*U*+D3)	Li^+ (PBE+*U*)	Li^+ (r2SCAN+rVV10)
**FC**	0.0	0.0	0.0
**off-FC**	Not Converged	492	1200
**TH**	216	288	438
**BC**	1338	4098	4014

S4. Arrhenius Plot, Trajectory Densities at 700 K and Radial Distribution Functions

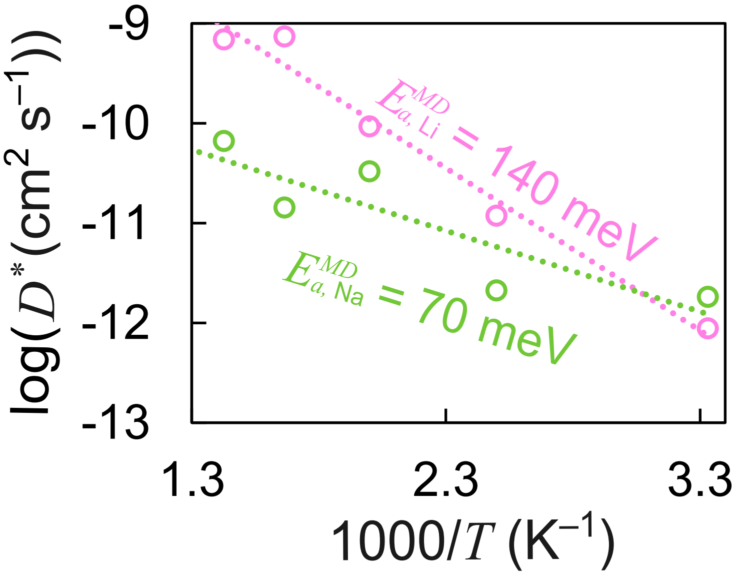

Figure S6. Arrhenius plot for Li^+ and Na^+ ions for a single 100 ps long production run. Good regression lines (R^2 as 0.96 and 0.76 for Li^+ and Na^+, respectively) are used to estimate the E_a^{MD} for Li^+ and Na^+ ions.

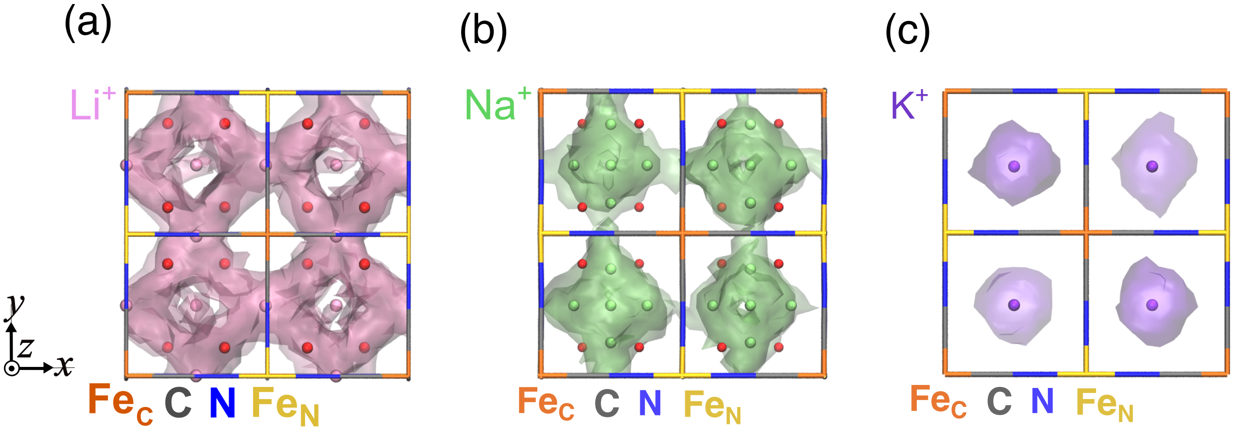

Figure S7. Trajectory densities at 700 K and ideal Wyckoff occultation sites (8c (BC, purple sphere), 24d (FC, pink sphere), 32f (TH, red sphere), and 48g (off-FC, lime sphere) sphere) for all A^+ (A = Li^+ (a), Na^+ (b), and K^+ (c) ions) accumulated for 100 ps simulations. Each panel displays the isosurfaces with an isovalue of 5.0×10^{-3} Å^{-3}. We excluded the first 10 ps of the MD simulations for that the system is equilibrated from our analysis. The blue, gray, yellow, and orange lines indicate N, C, the Fe-coordinating with N, and the Fe-coordinating with C, respectively.

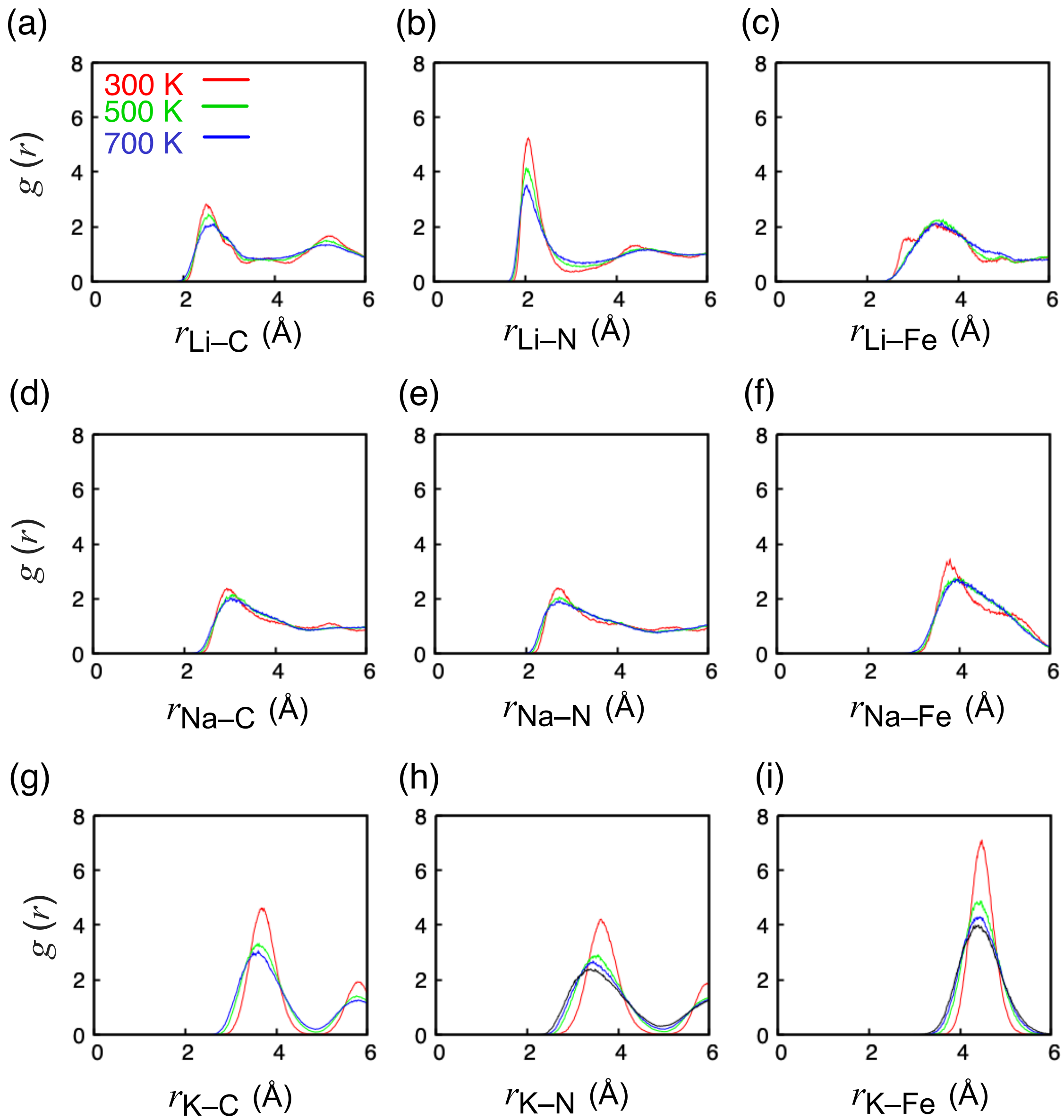

Figure S8. Radial distribution functions (RDFs) between A^+ (Li^+, Na^+, and K^+) ions and framework atoms (C, N, and Fe). The red, green, and blue lines correspond the RDFs at 300, 500, and 700 K. We use 0.01 Å bin width. We excluded the first 10 ps of the MD simulations for that the system is equilibrated from our analysis.

S5. Hopping Mechanism between Occupation Positions

Supporting Discussion

To better understand the hopping behavior of the A^+ ions between the occupation positions, we analyzed the probability densities of the dihedral angles of N_1–N_2–N_3–A^+ (the geometrical position of the labeled three N atoms in. the inset figure of Figures S8d) for our production runs.

Li^+ ions have the distributed probability densities at 300 K with the first peaked at 44° (black peak in Figure S8a), corresponding to the Wyckoff 24d sites (Table S2). The other peaks locate at 85, 95 and 170° (green, blue, and red lines in Figure S8a), considered as the occupancy of the FC positions. The time series data of N_1–N_2–N_3–Li^+ dihedral angles at 300 K shows four Li^+ ions take different dihedral degrees (Figure S9a). These distributions have second highest peaks at the dihedral angles for the Wyckoff 24d sites, illustrating the fingerprints of the Li^+ ions hop between the FC positions. At 700 K, the probability densities have wider distributions (Figure S8d). At 500 K and 700 K, the time series data of N_1–N_2–N_3–Li^+ dihedral angles shows that the four Li^+ ions more frequently take different dihedral degrees than the results at 300 K (Figures S9d and S9g). Hence, the Li^+ ions show more active hopping behavior at high temperature.

Na^+ ions have broader peaks of the probability densities of the dihedral angles, compared to Li^+ ions (Figure S8b). At 300 K, the first peaks appear at 13 ° (blue line in Figure S8b), corresponding to the Wyckoff 48f sites. The Time series data of N_1–N_2–N_3–Na^+ dihedral angles at 300 K (Figure S9b) shows all four Na^+ ions frequently take different dihedral degrees, which is the fingerprint of Na^+ ions actively hopping between the off-FC positions. Presumably, Na^+ ions primarily hop between the off-FC positions and take the FC and the TH positions as the barrier positions of their inter-cage and intra-cage hopping events. Compared to Li^+ ions, at 700 K, Na^+

ions have more localized peaks (Figures S8d and S8e). The changes of the probability densities between 300 K and 700 K are limited, indicating that Na^+ ions have the shallower potential landscape within in the cages.

K^+ ions also exhibit more localized peaks in the probability densities at 300 K, compared to Li^+ and Na^+ ions (Figure S8c). The peaks are located at –60, 25, and 132 °, corresponding to the Wyckoff 8c sites (Table S5). At 700 K, K^+ ions have the localized peaks (Figure S8f) and the time series data of N_1–N_2–N_3–K dihedral angles take the small fluctuations around the dihedral angels of the Wyckoff 8c sites in the unit cell (Figures S9f and S9i). These results confirm that K^+ ions do not hop between the BC positions.

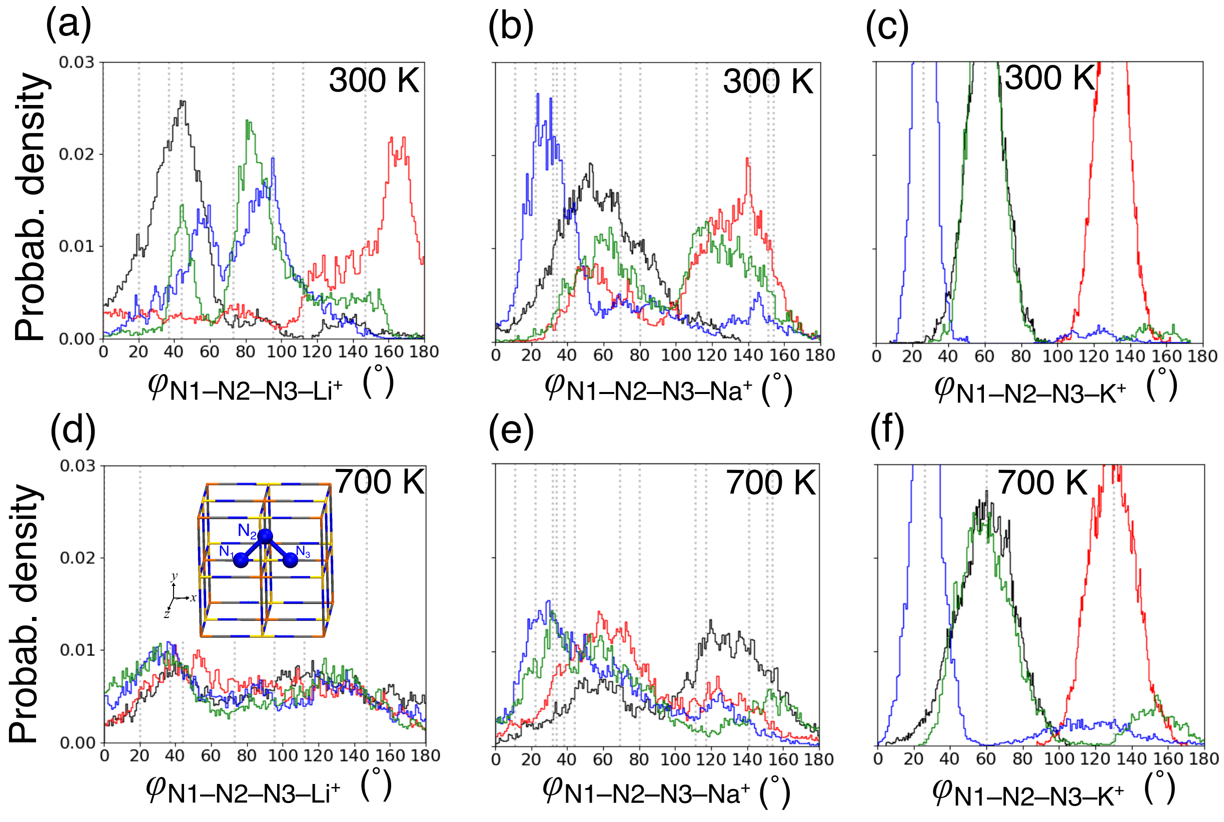

Figure S9. Probability densities for the N_1–N_2–N_3–A_i ($A \in$ Li, Na, and K) dihedral angles. To distinguish four A^+ ions in the unit cell, we used the red, green, blue, and black lines for different A^+ ions. The trajectories of accumulated for 100 ps simulations time at T = 300 and 700 K. We

discard the first 10 ps of the MD simulations from our analysis for equilibration. The probability densities are dividing probability with 1º bin width. In panel (d), the positions of the N_1, N_2, and N_3 atoms are shown in the inset figure. Gray lines correspond to the dihedral angels of the important Wyckoff sites, including the 24d sites in (a), and (d) panels, the 48g sites in (b), and (e) panels, and the 8c sites in (c), and (f) panels (detail values in Table S5).

Table S5. N_1–N_2–N_3–A^+ (A^+ = Li^+, Na^+, and K^+) dihedral angles of the Wyckoff sites.

Wyckoff site	Dihedral angles (º)
8c (BC)	26, 60, 130
24d (FC)	0, 20, 37, 44, 73, 95, 112, 147
48g (off-FC)	11, 22, 32, 34, 38, 44, 69, 80, 111, 117, 141, 151, 154

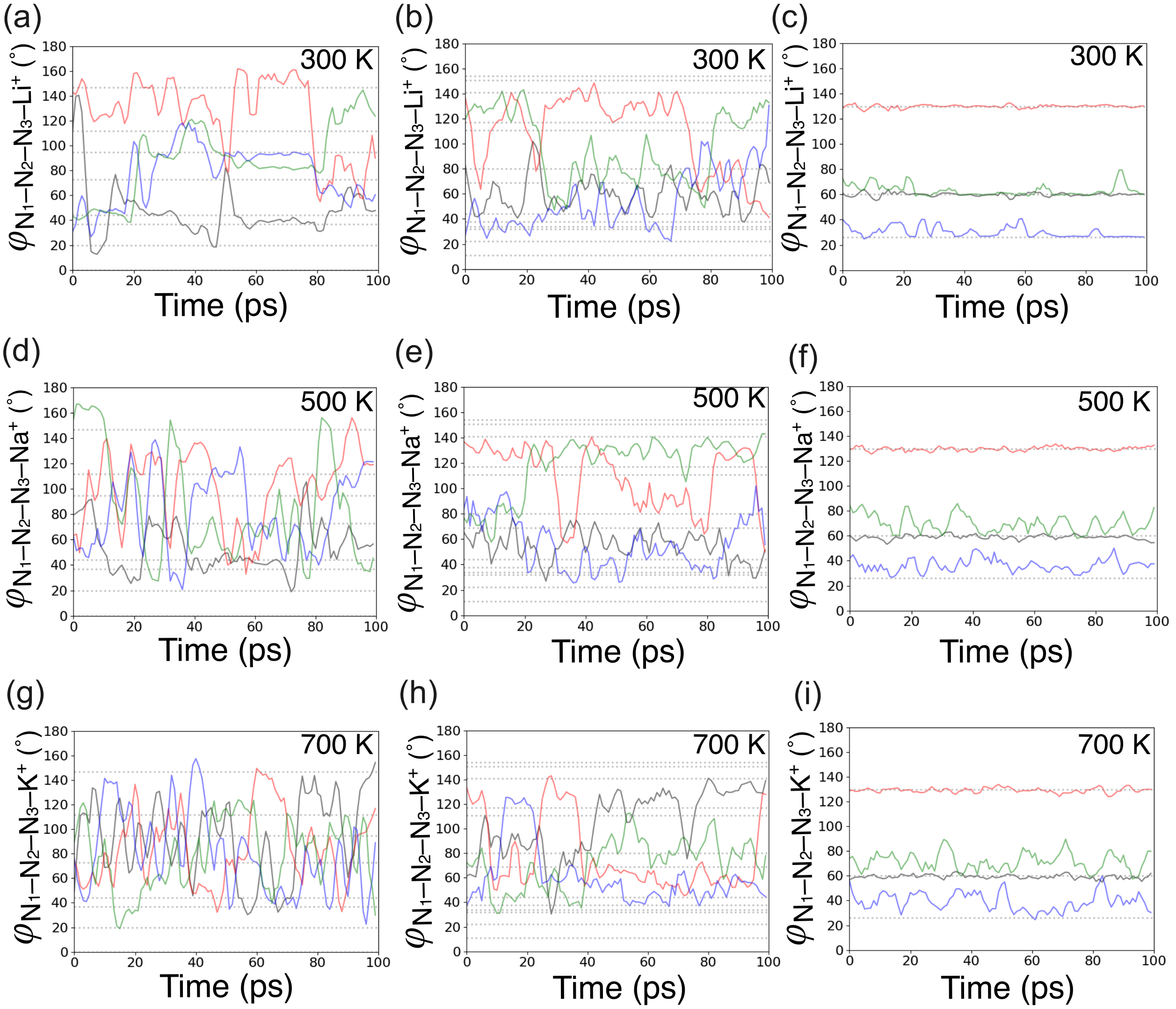

Figure S10. Time evolution of the averaged N_1–N_2–N_3–A^+ dihedral angles. Each dihedral angle for the A^+ ion is colored for clearly illustration. The positions of the N_1, N_2, and N_3 atoms are shown in the inset figure of Figure S9d. We excluded the first 10 ps of the MD simulations for that the system is equilibrated from our analysis. Each data point represents the averaged value in the range of the 100 MD steps (0.2 ps). Gray lines correspond to the dihedral angels of ideal occupation sites for A^+ ions. Gray lines correspond to the dihedral angels of the important Wyckoff sites, including the 24d sites in (a), (d), and (g) panels, the 48g sites in in (b), (e), and (h) panels, and the 8c sites in in (c), (f), and (i) panels (detail values in Table S5).

S6. Validation of the MSD and the Activation Energy via DFT-MD Calculations

Supporting Discussion

To identify computationally feasible settings for electronic structures calculations pertinent to the diffusivities of Li^+ and Na^+ ions, we systematically investigate how variations in electronic structure calculation parameters influenced the MSD for both ions. Our selection criteria encompassed several key factors related to Hamiltonian aspects, including spin polarization, the effective Hubbard on-site Coulomb repulsion correction for Fe atoms (U_{Fe}), and Grimme's D3 dispersion correction—as well as Self-Consistent Field (SCF) aspects —such as cutoff energy, energy convergence thresholds, FFT grid densities, and Projector-augmented wave (PAW).

Additionally, we evaluate alternative methods for integrating electron occupancies, specifically Gaussian and Fermi approaches. For each unique set of computational parameters, we perform MSD diagnostics over a 10 ps time frame at temperatures of 300, 500, and 700 K.

The conditions of our DFT-MD calculation are verified to follow the generated MSD values to follow Arrhenius equation. Specifically, we require MSDs at elevated temperatures to exceed those at lower temperatures, with self-diffusion coefficients (D^*) exhibiting a strong correlation to the Arrhenius equation ($R^2 > 0.9$). Given that Li^+ and Na^+ ions are anticipated to exhibit effective D^* near room temperature, we require that the MSD at 300 K show a positive slope to ensure accurate estimation of these D^*. Our findings are further contextualizing through a comparative analysis of the MSDs arising from our primary electronic structure calculation settings, in contrast with data derived from 15 additional parameter configurations, as detailed in Table S6.

Table S6. The comparison of the effect of the electronic structure calculation conditions on the mean square displacement (MSD) for Li^+ and Na^+ ions. We considered the effects from the aspect of the Hamiltonian and the SCF factors. As the Hamiltonian factors, we select the spin polarization, the on-site Coulombic repulsion for Fe atoms (U_{Fe}), Grimme's dispersion correction D3. As the SCF factors, we select the energy convergence (E_{diff}) threshold, the cutoff energy (E_{cutoff}), the FFT grid density (ρ_{FFT}), the partial occupancies for each electronic orbital ($f_{n\mathbf{k}}$) using Gaussian and Fermi smearing, and the PAW for Li^+, and Na^+ ions. For the PAW for Li^+, and Na^+ ions, we take the different number of electrons. We consider all electrons, and $2s^1$ electron for Li^+ ions and the $2s^22p^63s^1$, and the $2p^63s^1$ electrons for Na^+ ions. We require the MSD to exhibit (1) the trend that the values at low temperature do not exceed the one at high temperature, (2) a strong correlation to the Arrhenius equation (R^2 > 0.9), and (3) an effective self-diffusion coefficient at 300 K. We used the calculation condition in the first column as our main conditions. Using symbol of T. and F. standing for True and False, we show only different condition explicitly.

Fig.	D3	Spin	U_{Fe}	$f_{n\mathbf{k}}$	E_{cutoff}	E_{diff}	ρ_{FFT}	PAW (Li^+,Na^+)	(1)	(2)	(3)
-	**T.**	**T.**	**T.**	**Gauss**	**520**	**10^{-5}**	**Dense**	**($2s^1$, $2p^63s^1$)**	**T.**	**T.**	**T.**
S11	**F.**	-	-	-	-	-	-	-	T.	**T.**	**T.**
S12	-	**F.**	-	-	-	-	-	-	**T.**	F.	-
S13	-	-	**F.**	-	-	-	-	-	F.	-	-
S14	-	-	-	**Fermi**	-	-	-	-	F.	-	-
S15	-	-	-	-	**400**	-	-	-	F.	-	-
S16	-	-	-	-	-	**10^{-4}**	-	-	F.	-	-
S17	-	-	-	-	-	-	**Sparse**	-	F.	-	-
S18	-	-	-	-	-	-	-	**($1s^22s^1$, $2s^22p^63s^1$)**	**T.**	**-**	F.
S19	-	-	-	-	**400**	-	**Sparse**	-	**T.**	F.	-
S20	-	-	-	-	**400**	**10^{-4}**	-	-	**T.**	F.	-
S21	-	-	-	-	-	**10^{-4}**	**Sparse**	-	F.	-	-
S22	-	-	-	-	**400**	**10^{-4}**	**Sparse**	-	**T.**	F.	-
S23	-	-	-	-	**400**	**10^{-4}**	**Sparse**	**($1s^22s^1$, $2s^22p^63s^1$)**	F.	-	-
-	-	-	-	-	-	**10^{-6}**	**Highly dense**	-	F.	-	-
-	-	-	-	-	**650**	**10^{-6}**	**Highly dense**	-	F.	-	-

We compare the short MSD produced by different Hamiltonian setups (Figures S11-S13). Figure S12 presents the MSDs of Li^+ and Na^+ ions with and without Grimme's dispersion correction D3. Notably, for Na^+ ions, the MSD without D3 correction at 500 K exceeds that at 700 K (Figure S11b), indicating the necessity of the dispersion correction to generate MSDs at elevated temperatures exceeding those at lower temperatures. This is because the dispersion interactions significantly influence the accuracy of the Hellmann-Feynman forces acting on Na^+ ions on every MD step, due to their flat potential energy landscape. For all subsequent analyses, we consistently employ Grimme's dispersion correction D3 for our subsequent analyses.

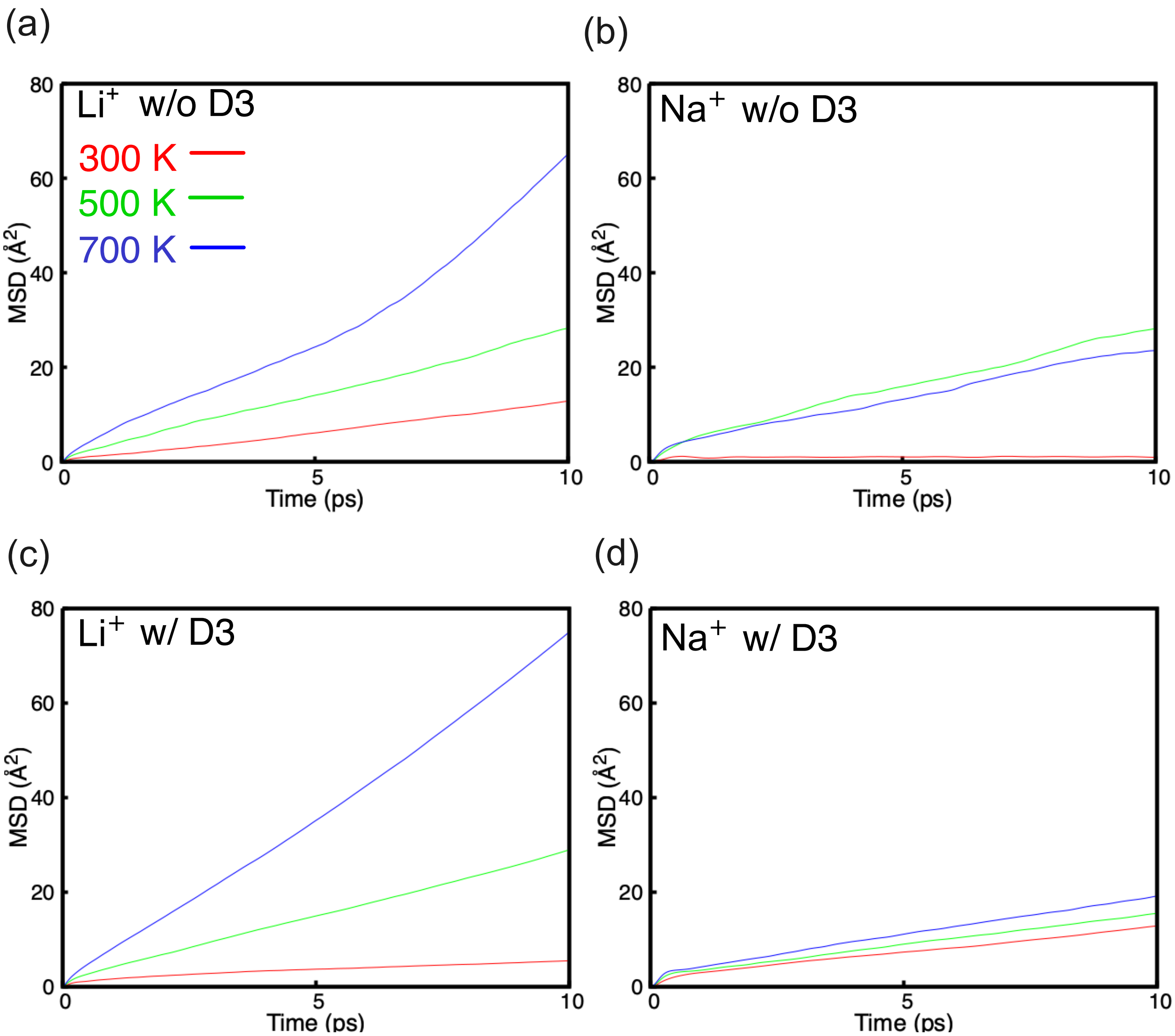

Figure S11 Comparison of the mean square displacement (MSD) for Li^+ and Na^+ ions at 300 (red lines), 500 (green lines), and 700 (blue lines). The MSDs obtained without Grimme's dispersion correction D3 for (a) Li^+ and (b) Na^+ ions. The MSDs obtained with Grimme's dispersion correction D3 for (c) Li^+ and (d) Na^+ ions. We exclude the first 10 ps of the MD simulations, for which the system is equilibrated, from our analysis.

Figure S12 illustrates the comparison of the MSDs with and without spin polarization. The MSD for Li^+ ions without spin polarization at 300 K is close to zero (red lines in Figure S12a), highlighting the importance of spin polarization in accurately modeling Li^+ ion diffusion at room temperature. In contrast, the MSD without spin polarization for Na^+ ions at 500 K and 700 K exhibit almost same (green and blue lines in Figure S12b), suggesting that spin polarization is crucial to generate MSDs at elevated temperatures exceeding those at lower temperatures. Therefore, spin polarization significantly impacts the accuracy of the Hellmann-Feynman forces acting on A^+-ions. For all subsequent analyses, we consistently employ the spin polarization for our subsequent analyses.

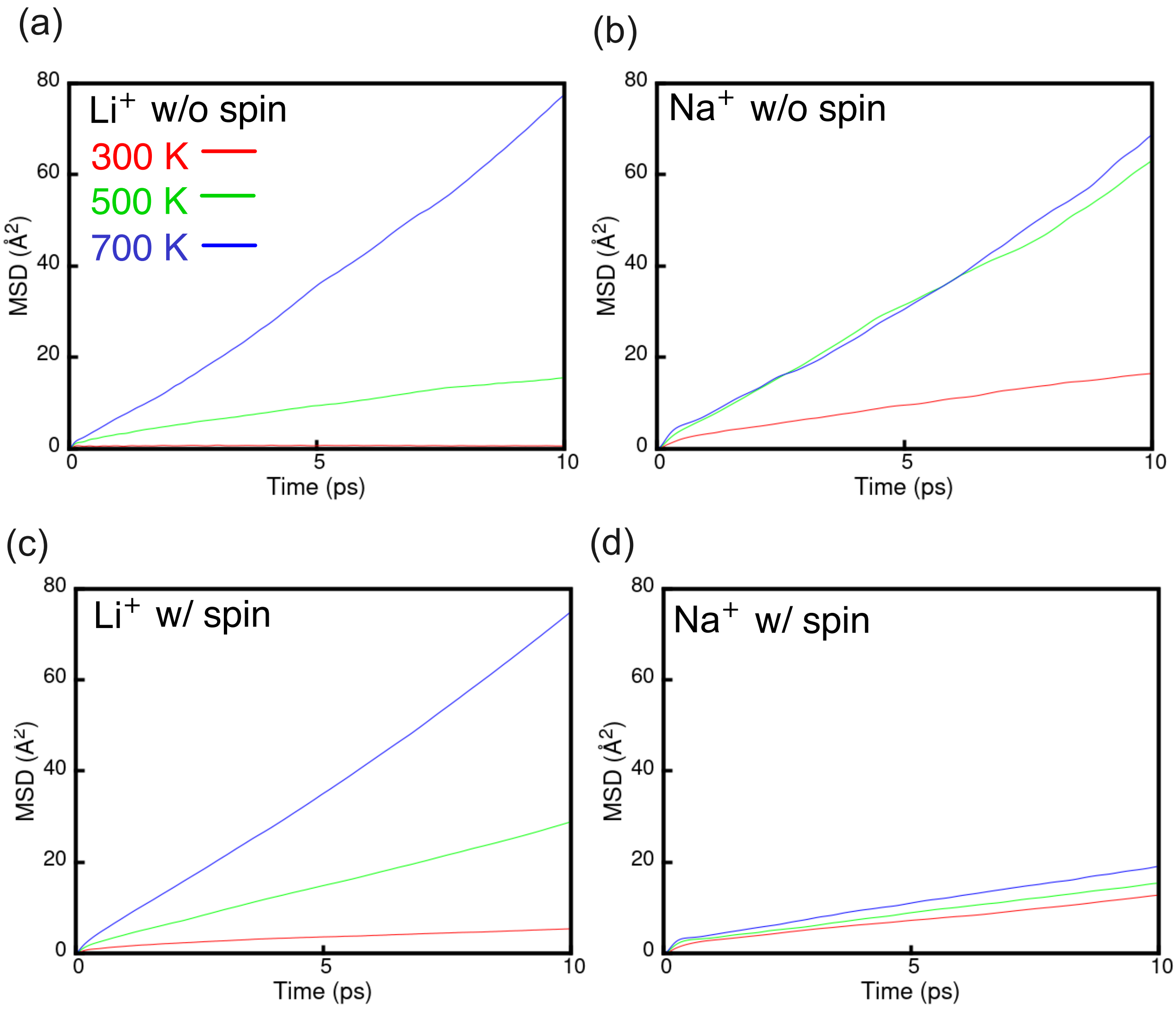

Figure S12. Comparison of the mean square displacement (MSD) for Li^+ and Na^+ ions at 300 (red lines), 500 (green lines), and 700 (blue lines). The MSDs obtained without spin polarization for (a) Li^+ and (b) Na^+ ions. The MSDs obtained with spin polarization for (c) Li^+ and (d) Na^+ ions. We excluded the first 10 ps of the MD simulations, for which the system is equilibrated, from our analysis.

Figure S13 presents the comparison of the MSDs with and without the U_{Fe}. For Li^+ ions, the MSD without the U_{Fe} at 300 K is close to zero (red line in Figure S13a), highlighting the critical role in accurately modeling Li^+ ions' diffusivities near room temperature. At 700 K, for Li^+ ions, the SCF calculations were not converged, therefore we excluded the MSD at 700 K. In contrast, Na^+ ions take the larger MSD than the MSD with U_{Fe}. Consequently, to compare the self-diffusivities of Li^+ and Na^+ ions, we consistently employ the U_{Fe} for our subsequent analyses.

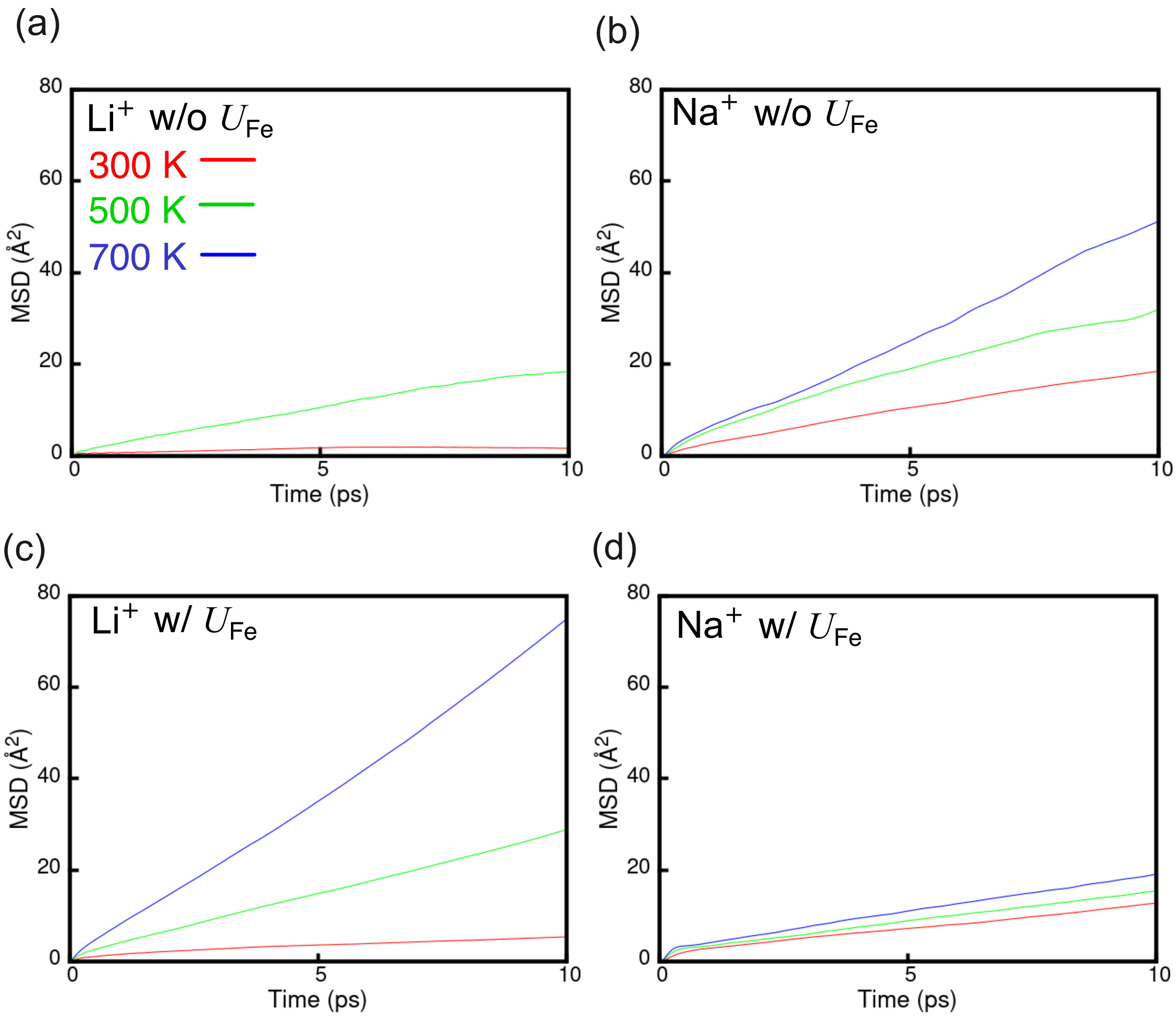

Figure S13. Comparison of the mean square displacement (MSD) for Li^+ and Na^+ ions at 300 (red lines), 500 (green lines), and 700 (blue lines). The MSDs obtained without the on-site Coulombic repulsion of the Fe ions for (a) Li^+ and (b) Na^+ ions. The MSDs obtained with the on-site Coulombic repulsion of the Fe ions for (c) Li^+ and (d) Na^+ ions. We exclude the first 10 ps of the MD simulations, for which the system is equilibrated, from our analysis. Note that we excluded the MSD at 700 K for Li^+ ions obtained without the on-site Coulombic repulsion of the Fe ions because the SCF calculation are not converged.

Figure S14 presents the comparison of the MSDs with Gaussian and Fermi approaches for integrating electron occupancies. For Li^+ ions, the MSD with the Fermi approach at 300 K takes almost zero (red line in Figure S14a), highlighting the critical role of the way of the treatment of the partial electron occupancies. Notably, for Na^+ ions, the MSD with the Fermi approaches at 300 and 500 K exceeds that at 700 K (green and blue lines in Figure S14b). This result indicates the necessity of the Gaussian approaches for integrating electron occupancies to generate the MSDs

for Na^+ ions at elevated temperatures exceed those at lower temperatures. For all subsequent analyses, we consistently employ Gaussian approach for integrating electron occupancies.

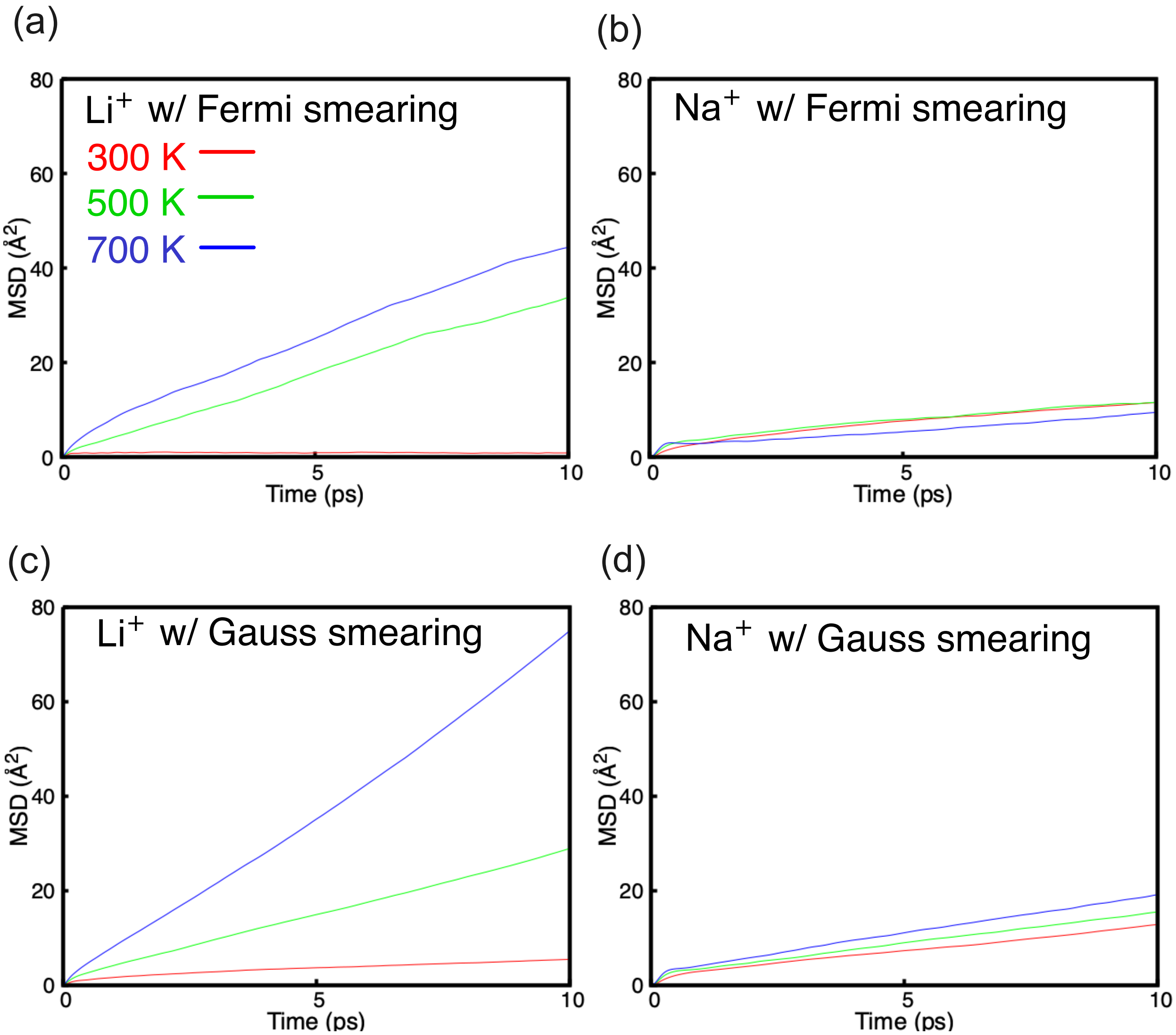

Figure S14. Comparison of the mean square displacement (MSD) for Li^+ and Na^+ ions at 300 (red lines), 500 (green lines), and 700 (blue lines). The MSDs obtained with the partial occupancies for the electronic orbital ($f_{n\mathbf{k}}$) using Fermi smearing for (a) Li^+ and (b) Na^+ ions. The MSDs obtained with the partial occupancies for each $f_{n\mathbf{k}}$ using Gaussian smearing for (c) Li^+ and (d) Na^+ ions. We excluded the first 10 ps of the MD simulations, for which the system is equilibrated, from our analysis.

Furthermore, we conducted a comparative analysis of the short MSD produced by different SCF conditions, specifically cutoff energy, energy convergence thresholds, FFT grid densities, and the PAW configurations of Li^+ and Na^+ ions. The interrelated nature of these SCF factors

necessitates a sequential discussion; we first examine the impact of each individual factor on the MSD, followed by an analysis of their combined effects on the resultant MSDs.

Figure S15 presents the MSDs with cutoff energies of 400 eV and 520 eV. At either temperature, the MSD of Li^+ ions with 400 eV cutoff energy takes the similar MSD with 520 eV cutoff energy. In contrast, the MSD of Na^+ ions at 300 K and 500 K exceeds that the one at 700 K (Figure S16b), highlighting the necessity of high cutoff energy for Na^+ ions.

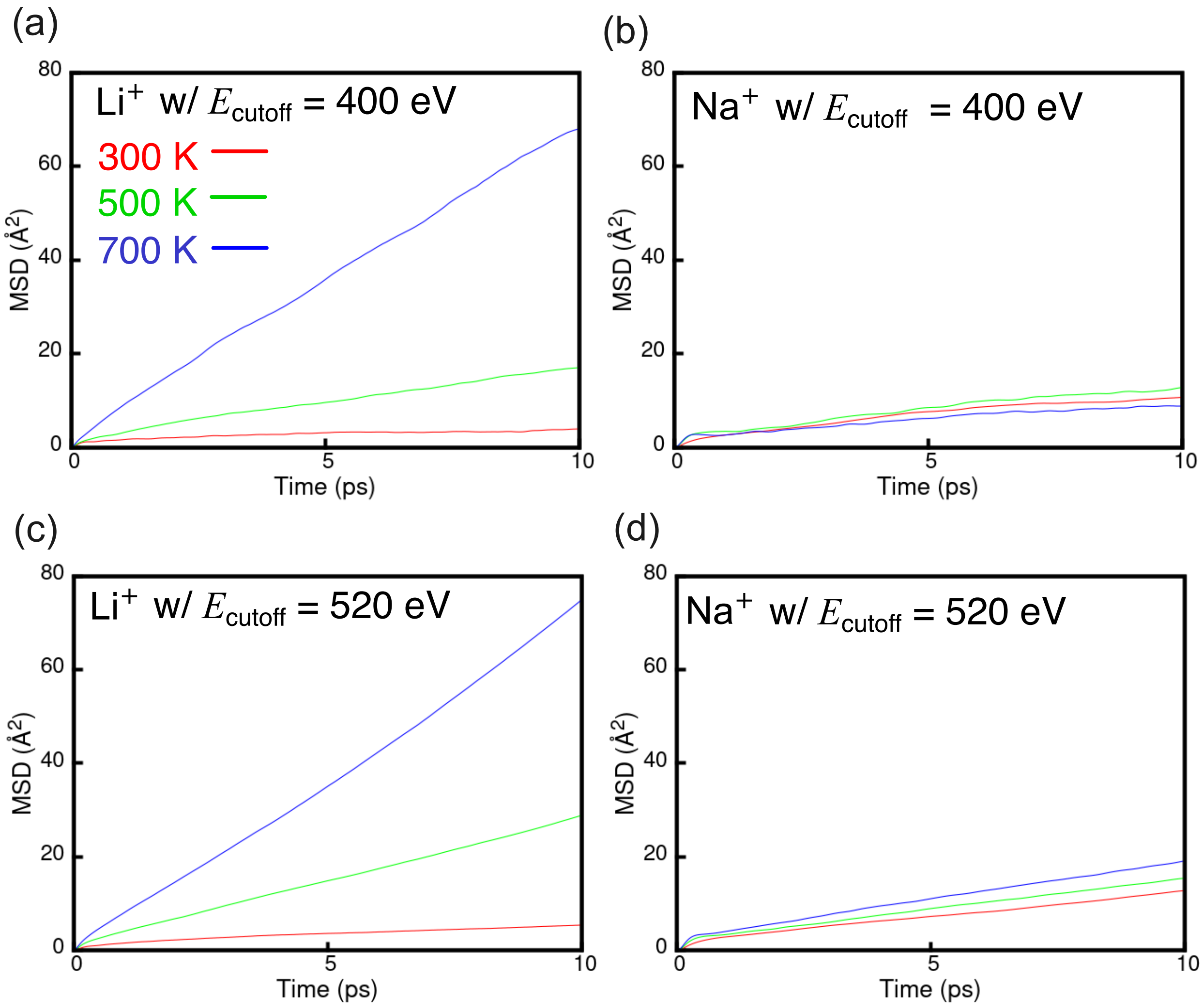

Figure S15. Comparison of the mean square displacement (MSD) for Li^+ and Na^+ ions at 300 (red lines), 500 (green lines), and 700 (blue lines). The MSDs obtained with 400 eV for (a) Li^+ and (b) Na^+ ions. The MSDs obtained with 520 eV for (c) Li^+ and (d) Na^+ ions. We excluded the first 10 ps of the MD simulations, for which the system is equilibrated, from our analysis.

Figure S16 illustrates the MSDs calculated with a 10^{-4} eV and 10^{-5} eV energy convergence threshold. For Li^+ and Na^+ ions, by using a 10^{-4} eV energy convergence threshold, the MSDs at elevated temperatures do not exceed those at lower temperatures (Figures S16a and S16b). These results highlight the necessity of strict energy convergence for Li^+ and Na^+ ions.

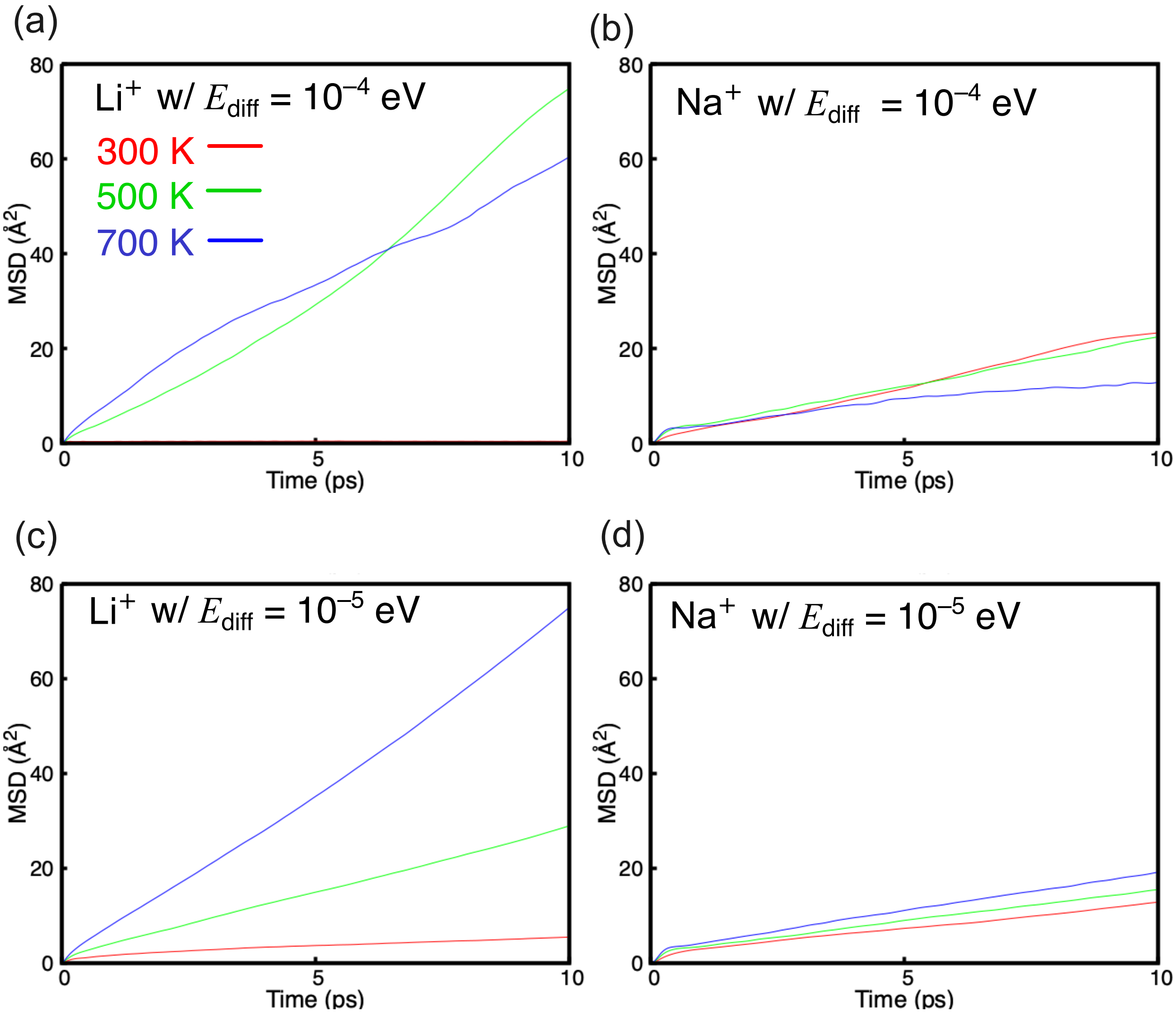

Figure S16. Comparison of the mean square displacement (MSD) for Li^+ and Na^+ ions at 300 (red lines), 500 (green lines), and 700 (blue lines). The MSDs obtained with 10^{-4} eV as the energy convergence for (a) Li^+ and (b) Na^+ ions. The MSDs obtained with 10^{-5} eV as the energy convergence for (c) Li^+ and (d) Na^+ ions. We excluded the first 10 ps of the MD simulations, for which the system is equilibrated, from our analysis.

Figure S17 shows the MSDs with the sparse and the dense FFT grid meshes. For Li^+ ions, the MSD with the sparse FFT densities at 300 K is close to zero, while, at 500 and 700 K, the MSDs take the similar value with the MSDs with the dense FFT densities (Figures S17a and S17c). For Na^+ ions, the MSDs with the sparse FFT densities at 300 and 700 K take the similar MSDs with the dense FFT densities (Figures S17b and S17d). In contrast, the MSD with the sparse FFT densities at 500 K is close to zero (blue line in Figure S17b). These results indicate the dense FFT density is critical to reproduce the effective self-diffusivities of Li^+ ions at room temperature and to generate the MSDs for Na^+ ions at elevated temperatures exceed those at lower temperatures.

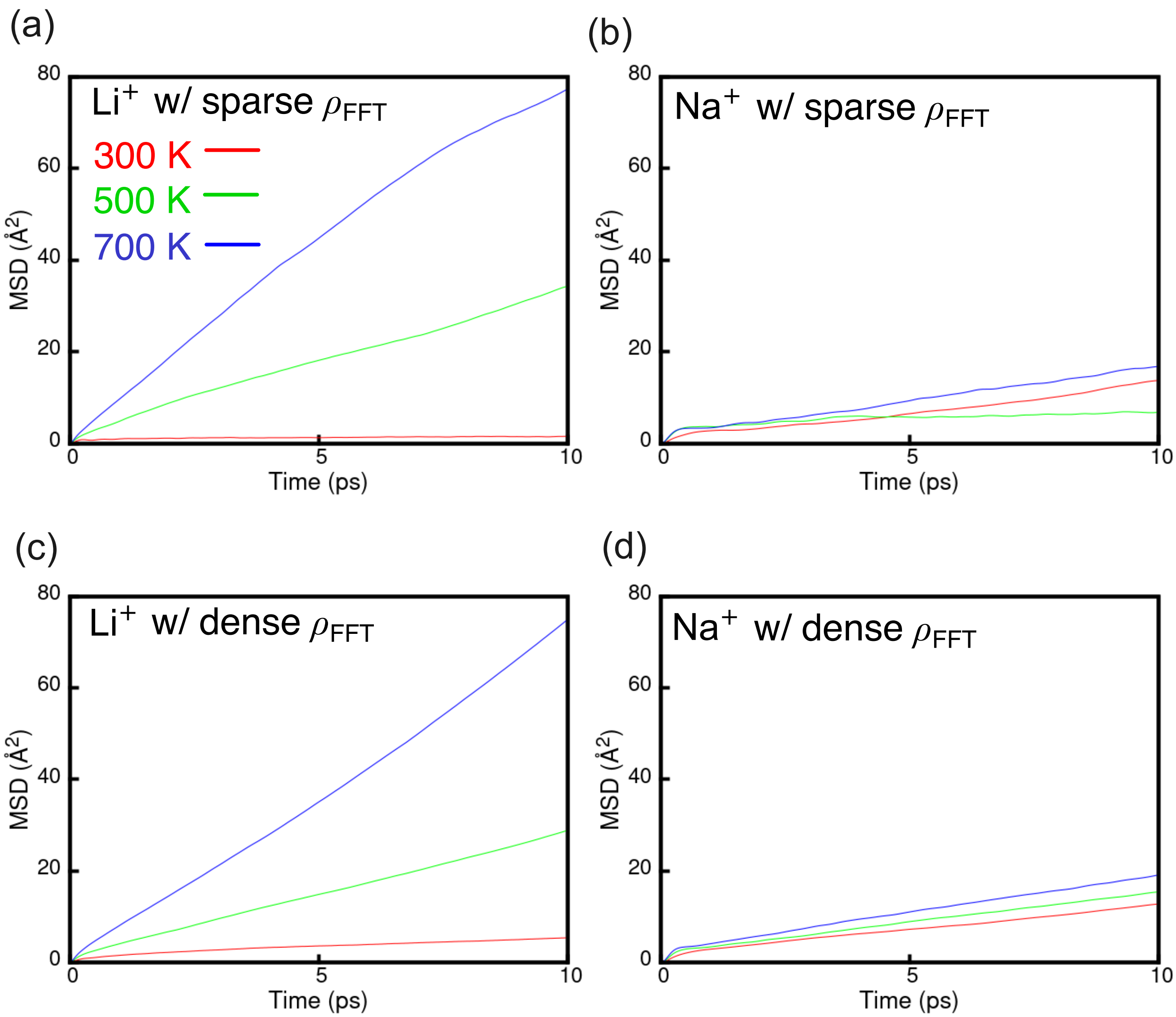

Figure S17. Comparison of the mean square displacement (MSD) for Li^+ and Na^+ ions at 300 (red lines), 500 (green lines), and 700 (blue lines). The MSDs obtained with sparse FFT grid densities for (a) Li^+ and (b) Na^+ ions. The MSDs obtained with dense FFT grid densities for (c) Li^+ and (d) Na^+ ions. We excluded the first 10 ps of the MD simulations, for which the system is equilibrated, from our analysis.

Figure S18 demonstrate the MSD with the different PAW of Li^+ and Na^+ ions. We considered the $1s^22s^1$ and $2s^1$ electrons for Li^+ ions and the $2s^22p^63s^1$ and $2p^63s^1$ electrons for Na^+ ions. The MSD with less and more considered electrons. For Li^+ ions with the PAW considering $1s^22s^1$ electrons, at either temperature, the MSDs take the similar MSD with the PAW considering $2s^1$ electrons (Figures S18a and S18c). For Na^+ ions with the $2s^22p^63s^1$ electrons, the MSD at 300 K is close to zero (Figure S18b). These results indicate that, to reproduce Na^+ ions' self-diffusivities

at room temperature, the using the PAW considering a more electrons of Na^+ ions is important. In contrast, for Li^+ ions, the effect of the using the PAW considering a more electrons of Li^+ ions on the MSD is limited.

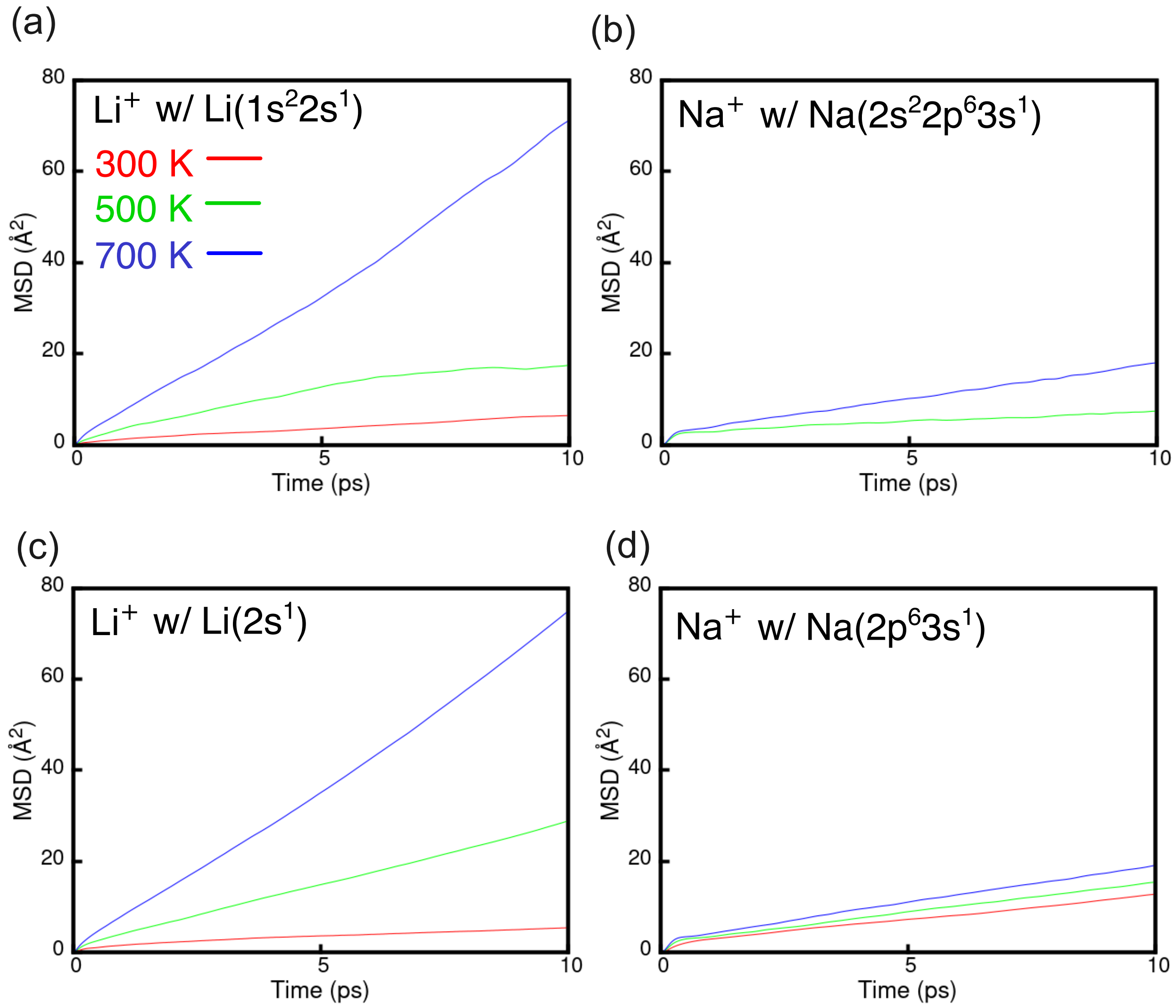

Figure S18. Comparison of the mean square displacement (MSD) for Li^+ and Na^+ ions at 300 (red lines), 500 (green lines), and 700 (blue lines). The MSDs obtained with the PAW with the larger number of considered electrons for (a) Li^+ and (b) Na^+ ions. The MSDs obtained with the PAW with the smaller number of considered electrons for (c) Li^+ and (d) Na^+ ions. We excluded the first 10 ps of the MD simulations, for which the system is equilibrated, from our analysis.

We investigated the interplay among cutoff energy, energy convergence thresholds, and FFT grid densities on MSDs as influenced by SCF parameters. By selecting two specific parameters,

we analyze three distinct configurations (Figures S19-S21). Among three SCF parameters (520 eV cutoff energy, 10^{-5} energy convergence threshold, and the FFT densities), we kept the single parmater and change two SCF conditions. Among three configurations, these MSDs of Na^+ ions exhibit a weak correlation ($R^2 < 0.9$). Therefore, we could not model Na^+ ions' self-diffusivities by changing two SCF parameters simultaneously.

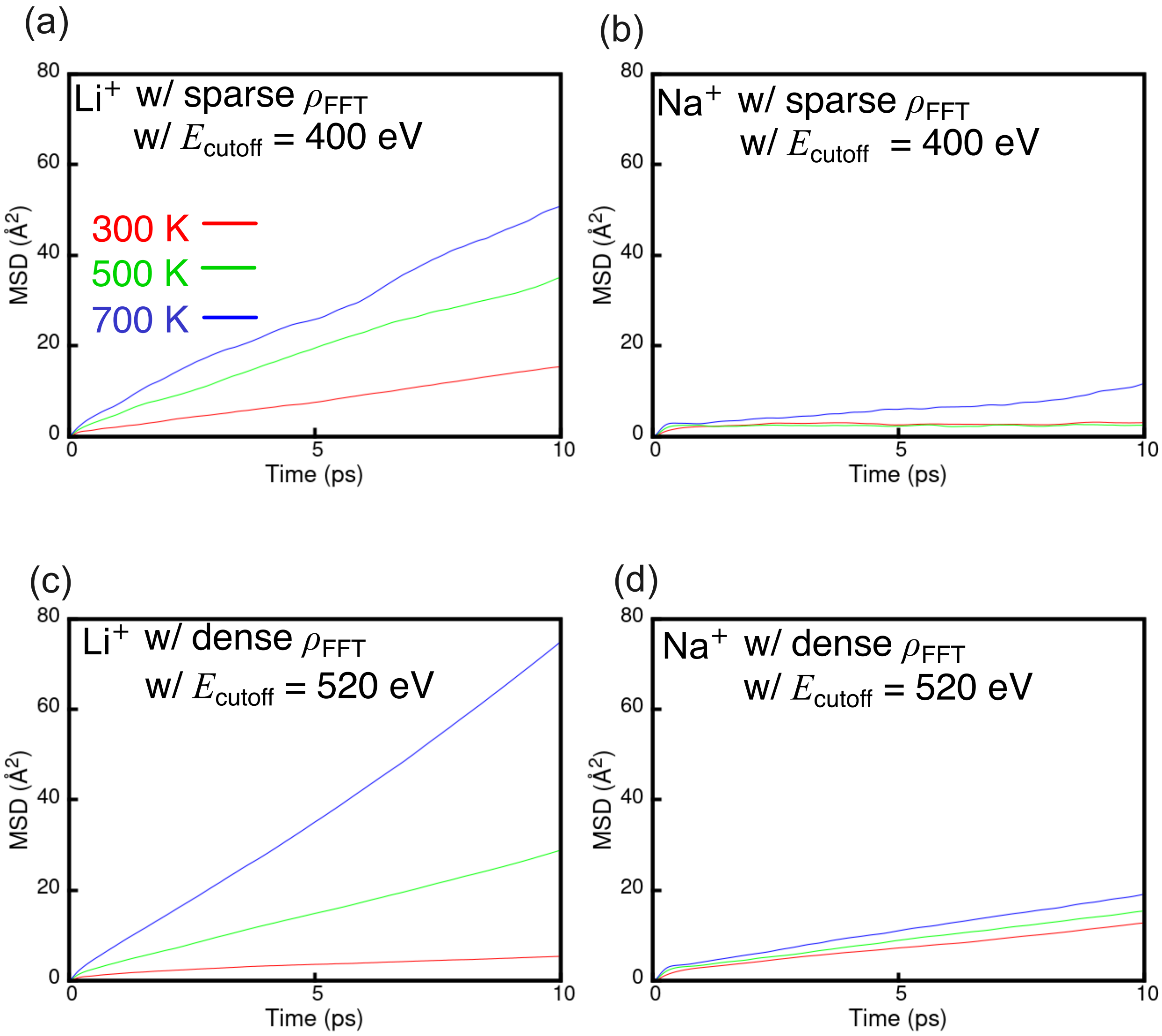

Figure S19. Comparison of the mean square displacement (MSD) for Li^+ and Na^+ ions at 300 (red lines), 500 (green lines), and 700 (blue lines). The MSDs obtained with 400 eV cutoff energy and the sparse meshes for FFT grid for (a) Li^+ and (b) Na^+ ions. The MSDs obtained with 520 eV cutoff energy and the dense meshes for FFT grid for (c) Li^+ and (d) Na^+ ions. We excluded the first 10 ps of the MD simulations, for which the system is equilibrated, from our analysis.

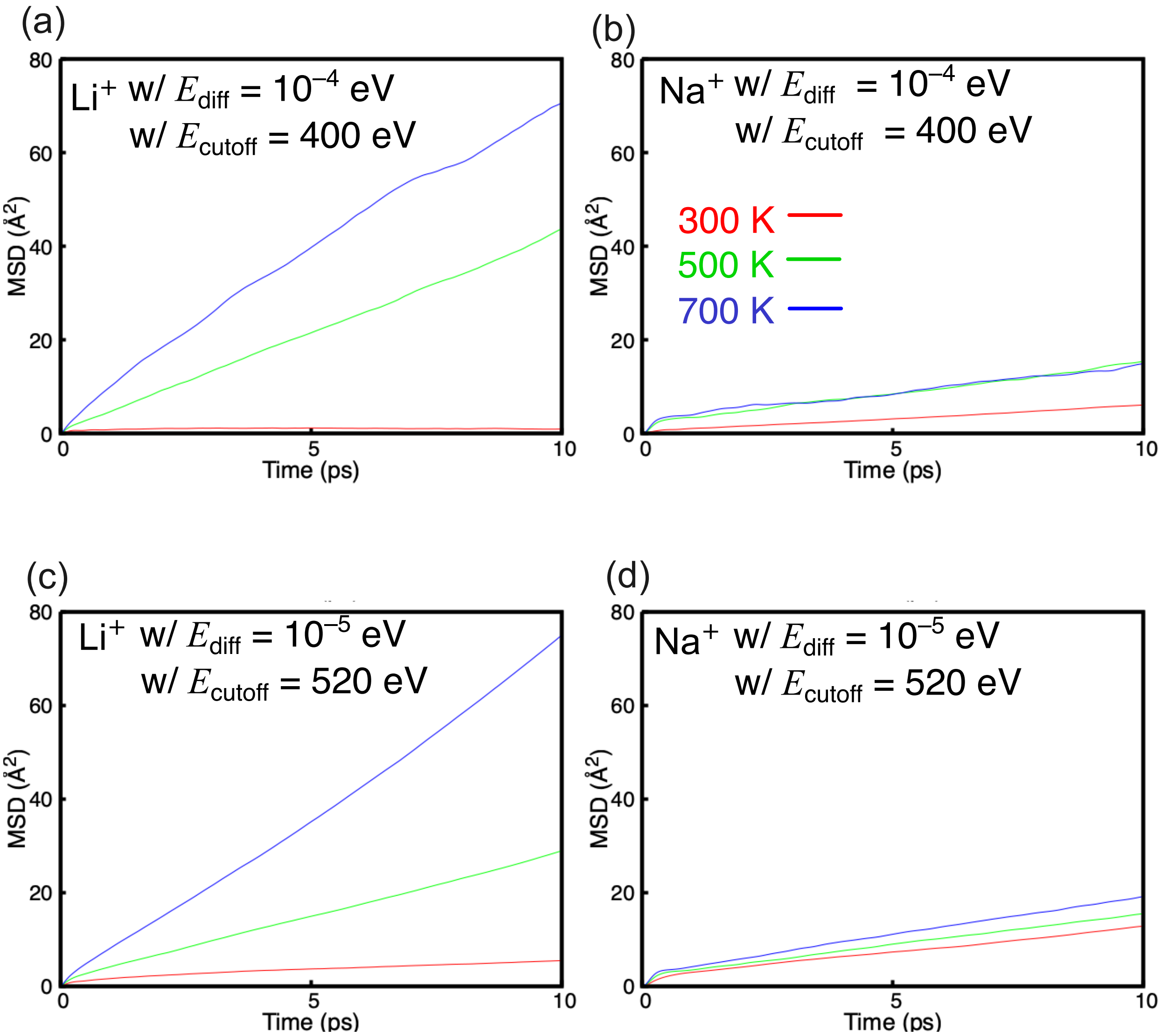

Figure S20. Comparison of the mean square displacement (MSD) for Li^+ and Na^+ ions at 300 (red lines), 500 (green lines), and 700 (blue lines). The MSDs obtained with 400 eV cutoff energy and the energy convergence less than 10^{-4} eV for (a) Li^+ and (b) Na^+ ions. The MSDs obtained with 520 eV cutoff energy and the energy convergence less than 10^{-5} eV for (c) Li^+ and (d) Na^+ ions. We exclude the first 10 ps of the MD simulations, for which the system is equilibrated, from our analysis.

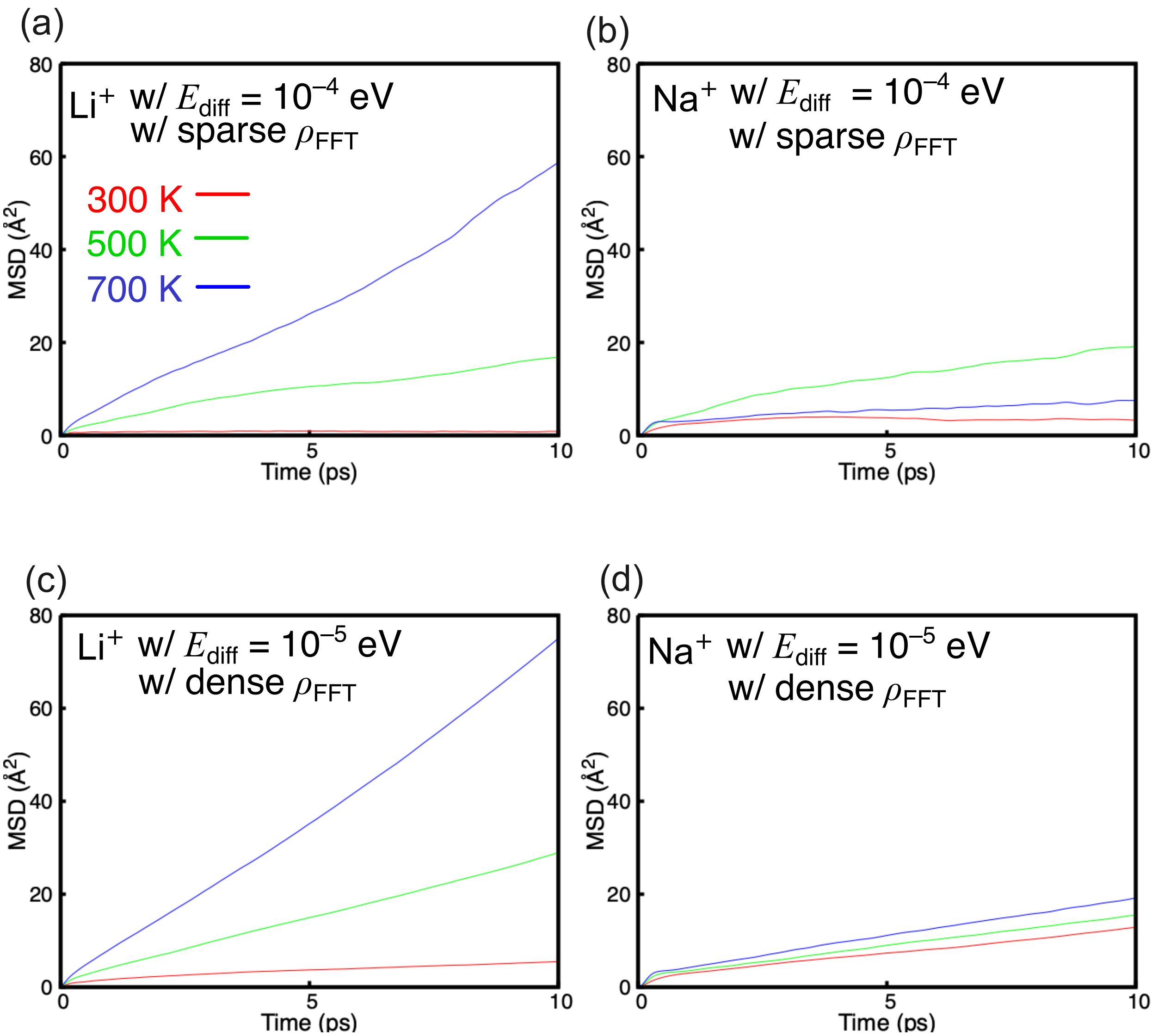

Figure S21. Comparison of the mean square displacement (MSD) for Li^+ and Na^+ ions at 300 (red lines), 500 (green lines), and 700 (blue lines). The MSDs obtained with the sparse meshes for FFT grid and the energy convergence less than 10^{-4} eV for (a) Li^+ and (b) Na^+ ions. The MSDs obtained with the dense meshes for FFT grid and the energy convergence less than 10^{-5} eV for (c) Li^+ and (d) Na^+ ions. We excluded the first 10 ps of the MD simulations, for which the system is equilibrated, from our analysis.

We investigate the interplay of the SCF parameters among cutoff energy, energy convergence thresholds, and FFT grid densities in relation to MSDs of Li^+ and Na^+ ions. We analyzed two distinct SCF parameter conditions. We simultaneously changed the three SCF parameters (*i.e.*, cutoff energies of 400 eV, 10^{-4} eV energy convergence, and sparse FFT in Figure S22).

Notably, careful attention must be given to the cutoff energy, FFT density, energy convergence, and PAW when modeling Li^+ and Na^+ ions. In fact, For Li^+ and Na^+ ions, the MSDs at elevated temperatures exceed those at lower temperatures during a short production run of 10 ps (Figure S22).

In addition, using 650 eV cutoff energy and much highly FFT density, we investigate whether stricter SCF conditions generate the MSDs at elevated temperatures exceeding those at lower temperatures. The SCF calculations of all production runs are not successfully converged, therefore the production runs, using much stricter SCF calculations are not suitable to discuss the diffusivities of Li^+ and Na^+ ions. Presumably, the fluctuation of DFT engines increase with increasing the temperature.

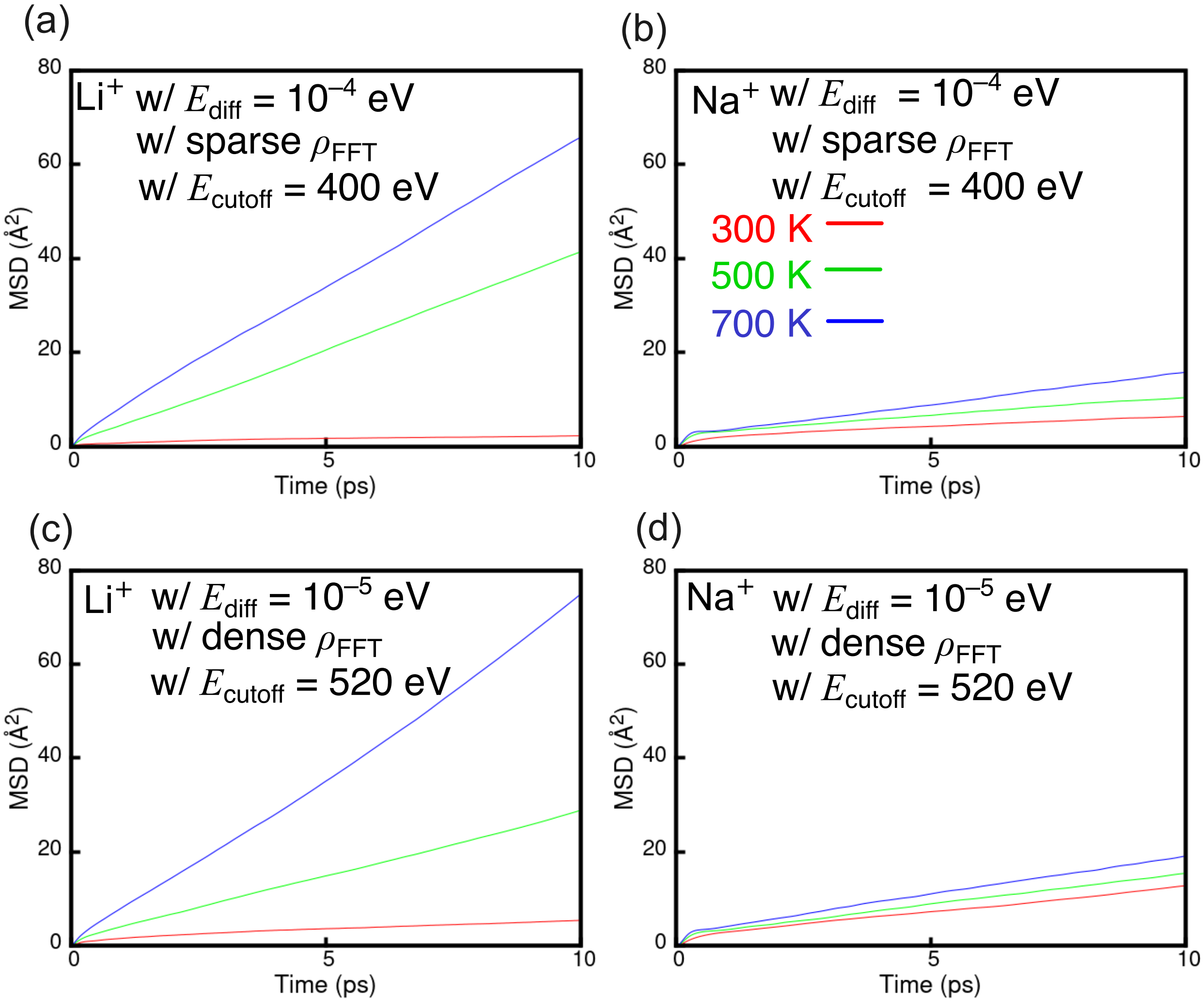

Figure S22. Comparison of the mean square displacement (MSD) for Li^+ and Na^+ ions at 300 (red lines), 500 (green lines), and 700 (blue lines). The MSDs obtained with 400 eV cutoff energy, the sparse meshes for FFT grid, and the energy convergence less than 10^{-4} eV for (a) Li^+ and (b) Na^+ ions. The MSDs obtained with 400 eV cutoff energy, the dense meshes for FFT grid, and the energy convergence less than 10^{-5} eV for (c) Li^+ and (d) Na^+ ions. We excluded the first 10 ps of the MD simulations, for which the system is equilibrated, from our analysis.

Furthermore, we examined the MSDs obtained from cutoff energies of 400 eV, 10^{-4} eV energy convergence, sparse FFT density, and the PAW with more considered electrons (Figure S23). For Li^+ ions, the MSD with at 300 K is close to zero (Figure S23a), while the MSDs for Na^+ ions

elevated temperatures do not exceed those at lower temperatures (Figure S23b). These results highlight the critical role of PAW in accurately modeling Li^+ self-diffusivities near room temperature and Na^+ ion self-diffusivities in wide range temperature.

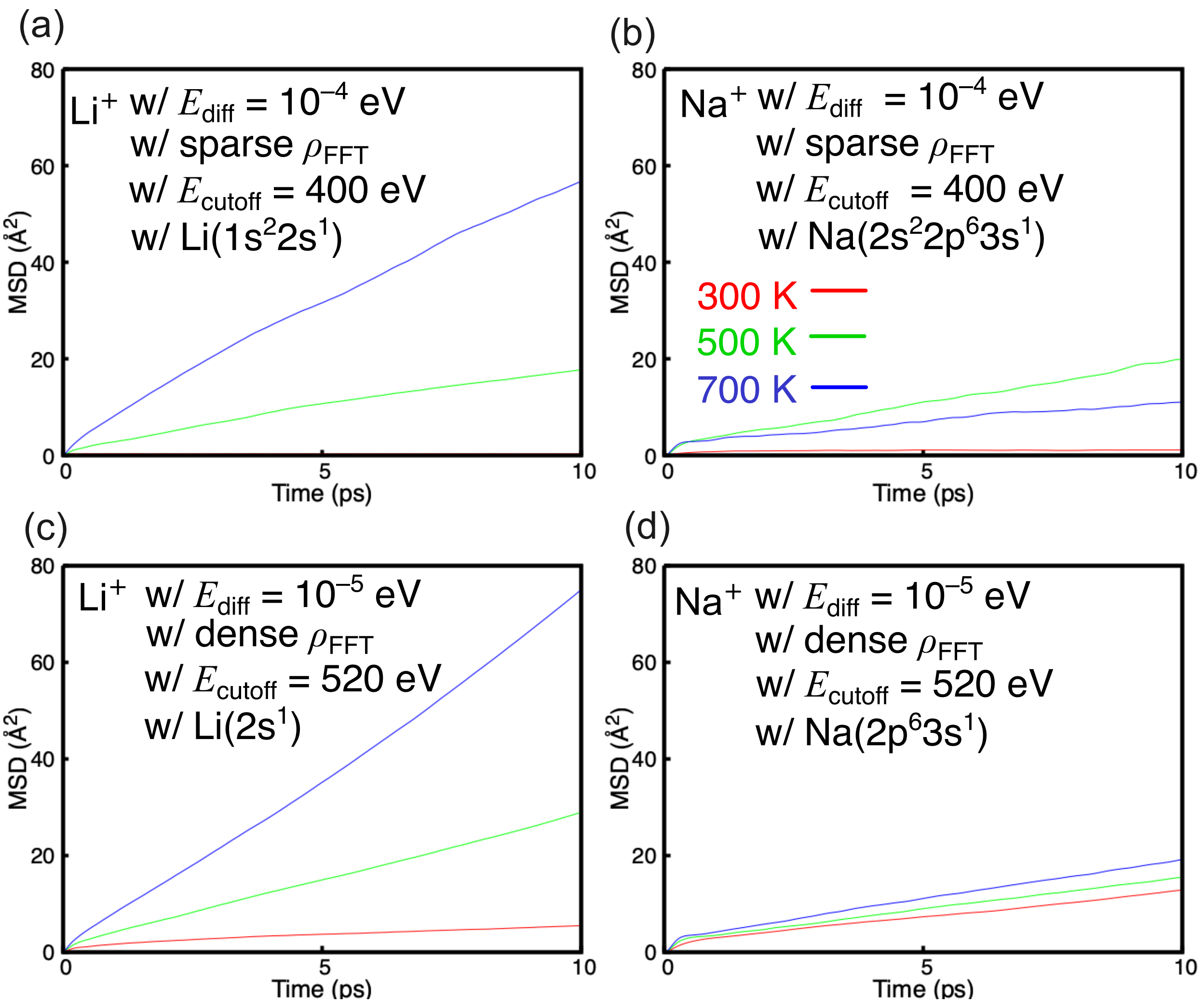

Figure S23. Comparison of the mean square displacement (MSD) for Li^+ and Na^+ ions at 300 (red lines), 500 (green lines), and 700 (blue lines). The MSDs obtained with 400 eV cutoff energy, the sparse meshes for FFT grid, the energy convergence less than 10^{-4} eV, and the PAW with the smaller number of considered electrons for (a) Li^+ and (b) Na^+ ions. The MSDs obtained with 400 eV cutoff energy, the dense meshes for FFT grid, the energy convergence less than 10^{-5} eV, and the PAW with the larger number of considered electrons for (c) Li^+ and (d) Na^+ ions. We excluded the first 10 ps of the MD simulations, for which the system is equilibrated, from our analysis.

To assess the correlation of both SCF configurations with the quality of MSDs, we performed a longer production run of 100 ps to derive D^* for Li^+ and Na^+ ions.

We selected three different calculation setups, which are without spin polarizations (Figures S12a and S12b), and the U_{Fe} (Figures S13a and S13b), and the combination of the three SCF conditions (400 eV, 10^{-4} eV energy convergence, sparse FFT density; Figure S22). The D^* for Li^+ and Na^+ ions produced by both without spin polarization and the effective Columbic repulsion U_{Fe} do not reproduce good quality of MSDs (Figures S22a and S22b). In contrast, the D^* calculated under the cutoff energies of 400 eV, 10^{-4} eV energy convergence, and sparse FFT density setup exhibit good quality of MSDs, despite a weaker correlation to the Arrhenius equation ($R^2 < 0.9$, Figure S24c). Therefore, we do not use 400 eV cutoff energies of, 10^{-4} eV energy convergence, and sparse FFT density setup.

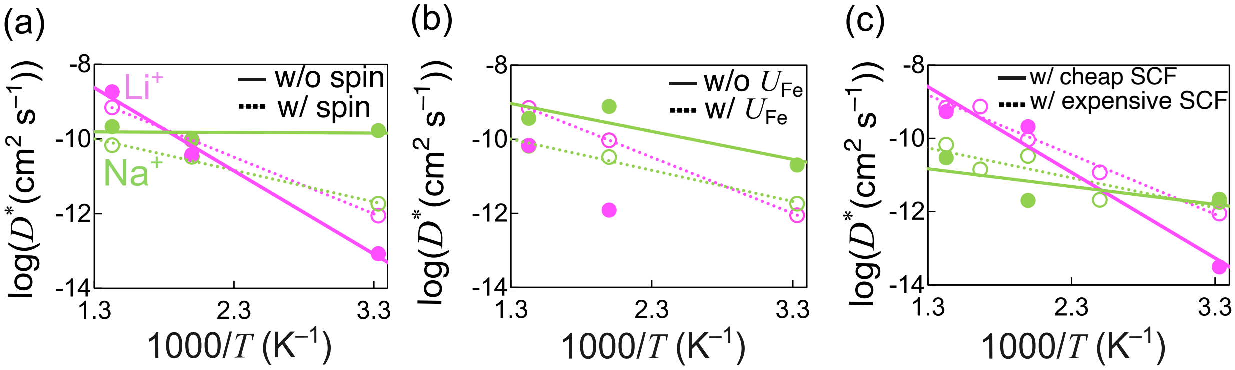

Figure S24. Comparison of the Arrhenius plot and its activation energy from the 100 ps MD (E_a^{MD}) with the different electronic structure calculation conditions for Li^+ (pink lines) and Na^+ (green lines) ions. The dash lines represent the results with our main condition (the first column in Table S6). (a) The self-diffusion coefficients (D^*) obtained without the spin polarization for Li^+ and Na^+ ions. (b) The D^*s obtained without the on-site Coulombic repulsion of the Fe ions for Li^+ and Na^+ ions. We discard the negligible D^* for Li^+ ions at 300 K. (c) The D^*s obtained with computational cheap SCF conditions (*i.e.,* 400 eV cutoff energy, the sparse meshes for FFT grid, and the energy convergence less than 10^{-4} eV) for Li^+ and Na^+ ions. We excluded the first 10 ps of the MD simulations, for which the system is equilibrated, from our analysis. The E_a^{MD} and the R^2 of these Arrhenius plot are shown in Table S7 and S8.

Table S7. Comparison of the effect of the electronic structure calculation conditions on the activation energy (E_a) and the R^2 of the Arrhenius plot (Figure S24) for Li^+ ions. We analyzed these E_a and the R^2, based on the long MD (100 ps) at 300, 500, and 700 K. We exclude the first 10 ps of the MD simulations, for which the system is equilibrated, from our analysis. The "NaN" means that we could not estimate the self-diffusion coefficients due to too small mean squares displacements.

MD production run ($E_{cutoff,}$ $E_{diff,}$ FFT density)	Computationally Cheap condition (400 eV, 10^{-4} eV, Sparce)	Computationally Heavy condition (520 eV, 10^{-5} eV, Dense)
Spin-polarized PBE+*U*+D3	$E_a^{MD} = 188$ meV<br>$R^2 = 0.96$	$E_a^{MD} = 131$ meV<br>$R^2 = 1.0$
Not spin-polarized PBE+*U*+D3	Not Calculated	$E_a^{MD} = 109$ meV<br>$R^2 = 1.0$
Spin-polarized PBE+D3	Not Calculated	NaN

Table S8. Comparison of the effect of the electronic structure calculation conditions on the activation energy (E_a) and the R^2 of the Arrhenius plot (Figure S24) for Na^+ ions. We analyzed these E_a and the R^2, based on the long MD (100 ps) at 300, 500, and 700 K. We exclude the first 10 ps of the MD simulations, for which the system is equilibrated, from our analysis.

MD production run ($E_{cutoff,}$ $E_{diff,}$ FFT density)	Computationally Cheap condition (400 eV, 10^{-4} eV, Sparce)	Computationally Heavy condition (520 eV, 10^{-5} eV, Dense)
Spin-polarized PBE+*U*+D3	$E_a^{MD} = 61$ meV<br>$R^2 = 0.51$	$E_a^{MD} = 73$ meV<br>$R^2 = 0.99$
Not spin-polarized PBE+*U*+D3	Not Calculated	$E_a^{MD} = 1$ meV<br>$R^2 = 0.007$
Spin-polarized PBE+D3	Not Calculated	$E_a^{MD} = 53$ meV<br>$R^2 = 0.51$

The author notes that future discourse on the dependence of the MSD on electronic structure calculations should be accompanied by a consideration of the statistical error. A preliminary conclusion is that the quality of the MSD can be improved under computationally expensive conditions. Furthermore, the anomalous behavior of the MSD at 600 K can be attributed to phase-space errors, potentially due to finite system size and finite duration of the production run (Figure 2b). This enhancement can be attributed to the accumulation of phase-space error resulting from the time-reversible integration of electronic degrees of freedom.[S7-S9]

S7. Probability Densities for the N–Fe$_N$–N Dihedral Angles for the A^+ Ions

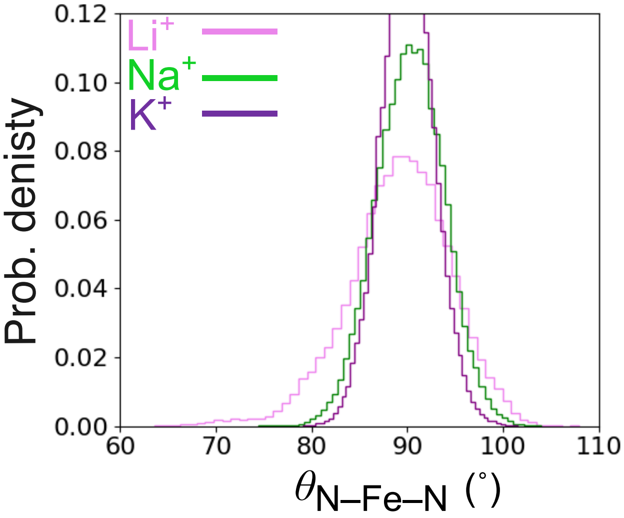

Figure S25. Probability densities for the averaged N–Fe$_N$–N dihedral angles ($\theta_{\mathrm{N-Fe-N}}$) for the A^+ ions at 300 K. We excluded the first 10 ps of the MD simulations for that the system is equilibrated from our analysis. We used all N–Fe$_N$–N angles with the two N–Fe$_N$ bonds joined vertically for these probability densities. We divided the probability densities with 1° bin width.

S8. Framework's Distortions for Occupation Positions via DFT Geometry Optimizations

When four Li^+ ions occupy the FC, the closest Fe–N bonds to Li^+ ions show significant distortions towards Li^+ ions ($min(\theta_{\mathrm{N-Fe-N}})$ = 84° the FC positions; Table S1). In addition, the $\varphi_{\mathrm{C-Fe-Fe-N}}$ angle in the *yz*-plane is a non-zero value, while the dihedral angles in the *xz*- and the *xy*-planes are zero. Consequently, when four Li^+ ions occupy the FC and the TH positions, the closest Fe–N bonds to the Li^+ ion distortions likely contribute to the positional stability, rather than the $a^-a^0a^0$ octahedral tilting. When four Na^+ ions occupy the (off-)FC, the closest Fe–N bonds to Na^+ ions exhibit small distortions toward Na^+ ion ($min(\theta_{\mathrm{N-Fe-N}})$ = 88°; see Table S1). These results can be rationalized by the steric repulsions caused by Na^+ ions.

Notably, when four Li^+ and Na^+ ions occupy the BC positions, the frameworks maintain $F\bar{m}3m$ symmetry (Table S1) and do not exhibit any framework distortions (Figures S3d and S4h). These results can be attributed to the BC positions, where the A^+ ions maximize their distances from both the framework and other A^+ ions within the cage. As a result, the Coulombic interactions between the A^+ ions and the framework are minimized among all occupation sites, leading to an undistorted framework unaffected by the A^+ ions. Based on the above results and discussions for Li^+ and Na^+ ions, the different types of framework distortions contribute to the stability of the occupation positions.

S9. Diffusion Pathway, Activation Energies and Framework Distortion via DFT-NEB Analysis.

For single-ion hopping mode, the Li^+ ion take the intra-cage pathway following a V-shaped pathway connecting the nearest neighbor FC positions between the *yz*-plane and the *xy*-plane (Figure S26a). Similarly, within a single cage, the Na^+ ion takes a V-shaped pathway connecting the nearest neighboring off-FC positions from close to the *yz*-plane to the *xy*-plane. The changes in $\theta_{\text{N-Fe-N,in }yz}$ and $\theta_{\text{N-Fe-N,in }xy}$ angles for Na^+ ions are also less pronounced (Figures S27b and S27d), compared to that for Li^+ ions.

For both diffusion modes, as the Li^+ and Na^+ ion approaches the *xy*-plane from the *yz*-plane, the $\theta_{\text{N-Fe-N,in }yz}$ increases while the $\theta_{\text{N-Fe-N,in }xy}$ decreases (Figures 6f and S27a-S27d). This trend is also found in the concerted mode. Particularly, for single ion hopping modes, the presence of A^+ ions lead to a rather reduction in the $\theta_{\text{N-Fe-N}}$ angle in the *xy*-plane (Figure S27d), contributing in higher E_a^{NEB}, than that of concerted modes (Figure 6f). Notably, when K^+ ions occupy the FC positions (red spheres in Figures 5c and S26c), the $\theta_{\text{N-Fe-N, in }yz}$ and $\theta_{\text{N-Fe-N, in }xy}$ angles exhibit the maximum deviation (red point in Figures S27a-S27d).

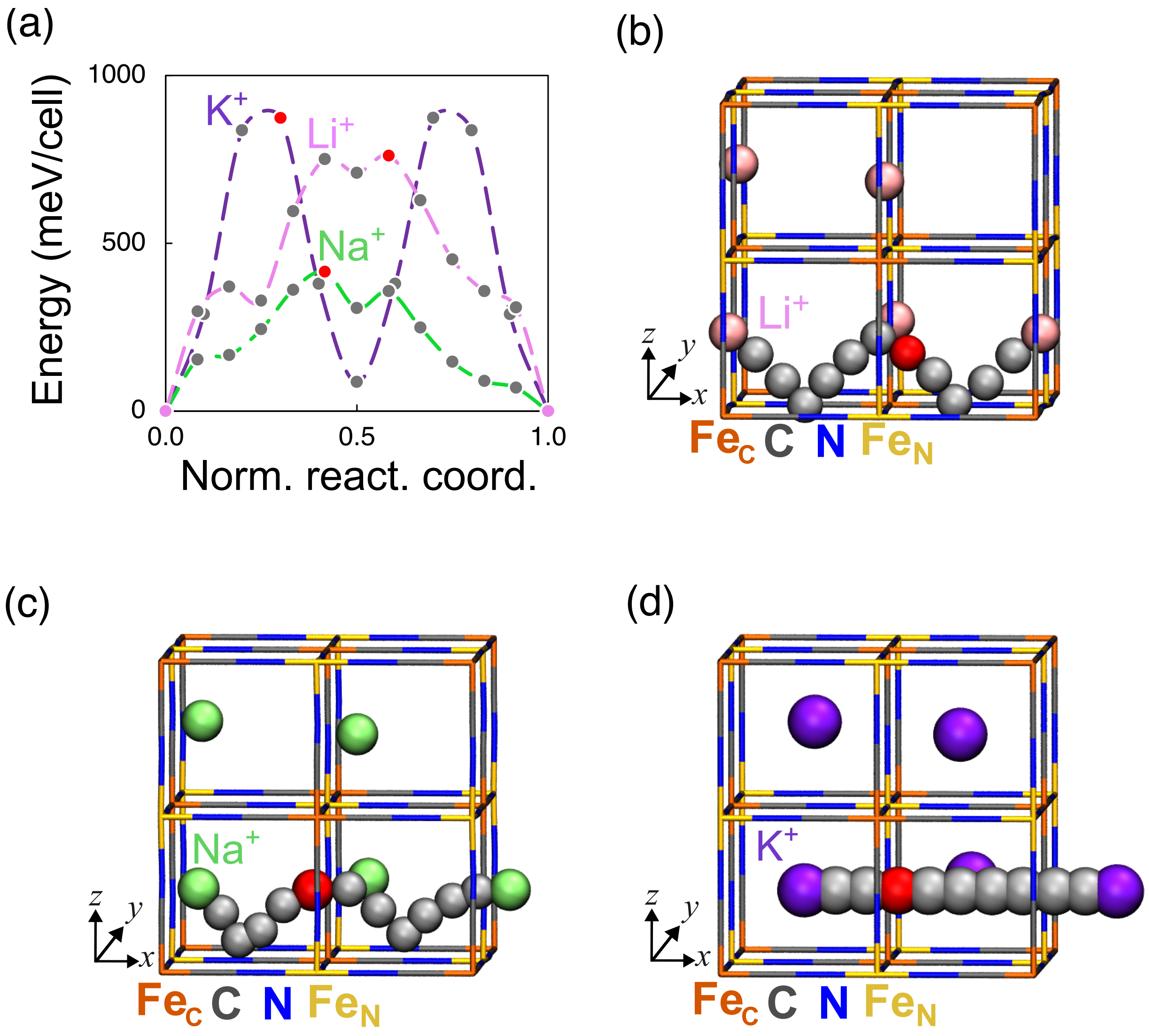

Figure S26. Single ion hopping mode for A^+ ions. (a) their corresponding potential profiles between most stable occupation positions and (b-d) The side views of the diffusion pathways. The Fe ions coordinating with N (C) atoms are with +3 (+2) valence in sextet (singlet) spin state. In panel (a-c), the pink, lime, and purple spheres for Li^+, Na^+, and K^+ ions correspond to the original FC, off-FC, and BC positions, respectively. The red spheres in the panels (b-d) represent to the barrier geometry of a single A^+ ion hopping mode, corresponding to the red cycle in panel (d). Note that in panels (b-d), we visualize the blue, gray, and yellow (orange) lines indicate the N atom, the C atom, and the Fe ion coordinating with the N (C) atoms to clearly display the pathways. We designated the label "Norm. react. coord." meaning normalized reaction coordinate.

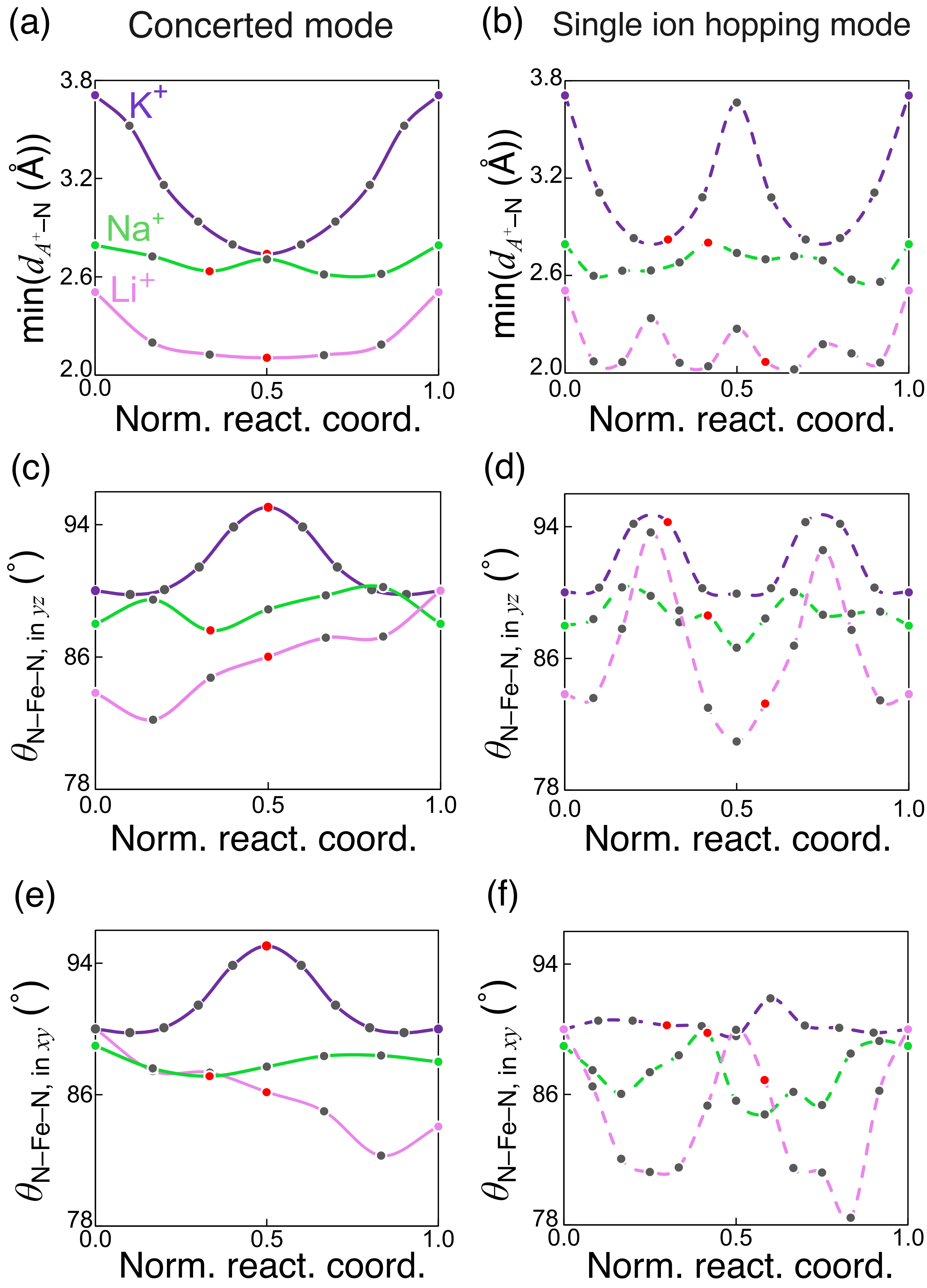
(a)
Concerted mode
(b)
Single ion hopping mode
K+
Na+
Li+
min(d_{A+–N} (Å))
3.8
3.2
2.6
2.0
0.0
0.5
1.0
Norm. react. coord.
(c)
(d)
θ_{N–Fe–N, in yz} (°)
94
86
78
(e)
(f)
θ_{N–Fe–N, in xy} (°)

Figure S27. (a-d) N–Fe_N–N angle (θ_{N-Fe-N}) and (e, f) the minimum A^+–N distance ((min (d_{A^+-N}))) for concerted and single ions hopping modes. We select the nearest neighbor Fe_N ion, and the first and second nearest neighbor N atoms from four A^+ ions for the θ_{N-Fe-N} angle in the *yz*- ($\theta_{N-Fe-N,\mathrm{in}\ yz}$) and the *xy*-plane ($\theta_{N-Fe-N,\mathrm{in}\ xy}$). The pink, lime, and purple lines correspond to the results of the PB with Li^+, Na^+, and K^+ ions, respectively. The solid and dashed lines represent the results of the concerted and the single A^+ ion hopping modes. The corresponding diffusion pathways and potential energy profiles are shown in Figure 6 and S26 for the concerted and single ion hopping modes, respectively. We designated the label "Norm. react. coord." meaning normalized reaction coordinate.

References

(S1) Matsuo, T.; Suga, H.; Seki, S. Thermodynamic Properties and Phase Transitions of Sodium Cyanide Crystal. *Bull. Chem. Soc. Jpn.* **1968**, *41*, 583–593.

(S2) Zhang, Z.; Avdeev, M.; Chen, H.; Yin, W.; Kan, W. H.; He, G. Lithiated Prussian Blue Analogues as Positive Electrode Active Materials for Stable Non-aqueous Lithium-ion Batteries. *Nat. Commun.* **2022**, *13*, 7790–7803.

(S3) You, Y.; Wu, X. L.; Yin, Y. X.; Guo, Y. G. High-Quality Prussian Blue Crystals as Superior Cathode Materials for Room-Temperature Sodium-Ion Batteries. *Energy Environ. Sci.* **2014**, *7*, 1643–1647.

(S4) Eftekhari, A. Potassium Secondary Cell Based on Prussian Blue Cathode. *J. Power Sources* **2004**, *126*, 221–228.

(S5) Buser, H. J.; Schwarzenbach, D.; Petter, W.; Ludi, A. The Crystal Structure of Prussian Blue: $Fe_4[Fe(CN)_6]_3 \cdot xH_2O$. *Inorg. Chem.* **1977**, *16*, 2704–2710.

(S6) Kumar, A.; Yusuf, S. M.; Keller, L. Structural and Magnetic Properties of $Fe[Fe(CN)_6] \cdot 4H_2O$. *Phys. Rev. B* **2005**, *71*, 1–7.

(S7) Herbert., J., M.; Head-Gordon, M. Accelerated, Energy-conserving Born–Oppenheimer Molecular Dynamics *via* Fock Matrix Extrapolation. Phys. *Chem. Chem. Phys.* **2005**, *7*, 3269–3275.

(S8) Pulay. P.; Fogarasi. G. Fock Matrix Dynamics. *Chem. Phys. Lett.* **2004**, *386*, 272–278.

(S9) Niklasson, A., M. N.; Tymczak C. J.; Challacombe, M. Time-reversible Born-Oppenheimer Molecular Dynamics. *Phy. Rev. Lett.* **2006**, *97*, 123001–123005.
